# Temperature-Dependent Structural and Optoelectronic Properties of the Layered Perovskite 2-Thiophenemethylammonium Lead Iodide

**DOI:** 10.1021/acs.jpcc.4c03221

**Published:** 2024-07-25

**Authors:** Justas Deveikis, Marcin Giza, David Walker, Jie Liu, Claire Wilson, Nathaniel P. Gallop, Pablo Docampo, James Lloyd-Hughes, Rebecca L. Milot

**Affiliations:** † Department of Physics, University of Warwick, Coventry CV4 7 AL, United Kingdom; ‡ School of Chemistry, University of Glasgow, Glasgow G12 8QQ, United Kingdom

## Abstract

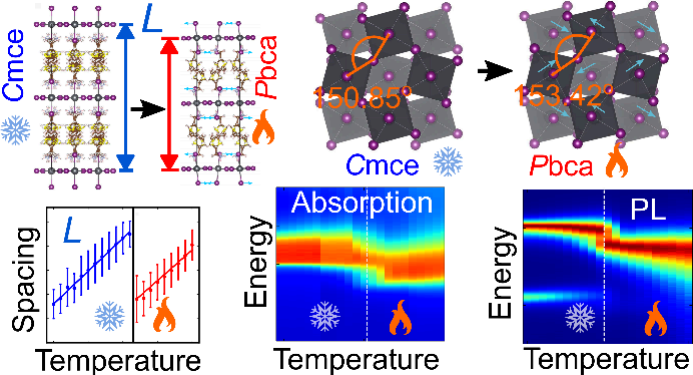

Improved knowledge of the influence of temperature upon layered perovskites is essential to enable perovskite-based devices to operate over a broad temperature range and to elucidate the impact of structural changes upon the optoelectronic properties. We examined the Ruddlesden–Popper layered perovskite 2-thiophenemethylammonium lead iodide (ThMA_2_PbI_4_) and observed a structural phase transition between a high- and a low-temperature phase at 220 K using temperature-dependent X-ray diffraction, UV–visible absorption, and photoluminescence (PL) spectroscopy. The structural phase transition altered the tilt pattern of the inorganic octahedra layer, modifying the absorption and PL spectra. Further, we found a narrow and intense additional PL peak in the low-temperature phase, which we assigned to radiative emission from a defect-bound exciton state. In both phases we determined the thermal expansion coefficient and found values similar to those of cubic 3D perovskites, i.e., larger than those of typical substrates such as glass. These results demonstrate that the organic spacer plays a critical role in controlling the temperature-dependent structural and optoelectronic properties of layered perovskites and suggests more widely that strain management strategies may be needed to fully utilize layered perovskites in device applications.

## Introduction

### Emergence of Layered Perovskites

Metal halide perovskites are known for their outstanding optoelectronical properties, such as long carrier lifetimes (>100 ns),^1^ efficient radiative interband processes (reaching 50% at charge carrier densities of 10^17^ cm^–3^), and reasonable charge carrier mobilities (10 to 35 cm^2^ V^–1^ s^–1^),^2,3^ which enable applications in photovoltaics, light-emitting diodes,^4^ and photodetectors.^5^ Layered perovskites are derivatives of their 3D counterparts and use long organic cations to separate adjacent metal halide octahedra sheets:^6,7^ this limits charge carrier motion between perovskite layers, increasing the degree of quantum confinement. Further, the high mismatch in dielectric constant between the organic spacer and metal halide layers boosts the exciton binding energy. The prominent excitons in layered perovskites exhibit high photoluminescence quantum yields of 80% or higher,^4,8,9^ offering potential in light-emitting applications.^4,10^ Layered perovskites are more stable under atmospheric environment than their 3D counterparts: the organic spacers hinder detrimental effects such as decomposition and ion migration, leading to solar cells with improved longevity.^11,12^ Furthermore, the optoelectronic and structural properties of these materials are related, as manifested by the interaction of an organic spacer and inorganic lattice,^13^ which results in the distortion of the metal halide octahedra.^14,15^ Understanding the relationship between structural and optoelectronic properties is important to enable the targeted design of layered perovskites for device applications.

### Effect of Temperature on Functional Properties

Devices, and in particular solar cells, are required to operate over a range of temperatures, and hence knowledge of how the optoelectronic properties vary with temperature can guide the design and operation of efficient devices. The temperature has been shown to influence fundamental properties of both layered and 3D metal halide perovskites, such as the bandgap and PL emission energies,^16−19^ and charge-carrier dynamics.^20−24^ Furthermore, a variation in temperature can cause thermal expansion/contraction of the crystal lattice and induce structural phase transitions, altering the electronic bandstructure and changing the optoelectronic properties. For example, methylammonium lead iodide (CH_3_NH_3_PbI_3_ or MAPI) transitions between orthorhombic and tetragonal at 160 K and from tetragonal to cubic at 315 K, and both of these phase transitions are marked by changes in the bandgap and photoluminescence (PL) energies.^20^

In addition to modifying the optoelectronic parameters, additional temperature-induced changes to the physical properties need to be considered in operating devices. For example, the thermal expansion coefficient of the perovskite can impact the film’s mechanical stability.^25^ Cracks can form in perovskite thin films at elevated temperatures, which reduces the efficiency of double- and triple-cation perovskite solar cells.^26^ The formation of cracks can be explained by the grain size increasing with temperature, which further increases the strain across grain boundaries and eventually causes cracks to form in the cell.^27^ Furthermore, elevated temperatures up to 100 °C are often used in the fabrication of a perovskite thin film, either during crystallization or in a postgrowth annealing step.^28^ This can result in the perovskite film exhibiting a residual strain, as the thermal expansion coefficients of the substrate the film is grown on and the perovskite layer can differ substantially.^29−32^ Residual strain plays an important role in the stability of devices, affects the lattice parameters, and can alter the optoelectronic properties^29−32^ as well as enhance degradation under illumination due to a reduced activation energy for ion migration.^28^ These effects need to be considered when designing perovskite-based devices with optimum performance and longevity.

### Motivation to Study ThMA_2_PbI_4_

Despite the continued development of layered perovskite materials and their accelerating deployment in applications, their temperature-dependent properties have not been studied extensively, beyond a handful of the more commonly used compounds. Among a large number of organic spacers available, we decided to investigate the Ruddlesden–Popper layered perovskite (ThMA_2_PbI_4_) with a 2-thiophenemethylammonium (ThMA) molecule as an organic spacer due to its excellent performance in solar cells.^33−36^ ThMA_2_PbI_4_ is a promising material since it is anticipated that the electron-rich thiophene in the ThMA improves charge transport properties between the inorganic layers.^34,37^

To gain a full picture of this technologically applicable layered perovskite we investigated its temperature-dependent structural and optoelectronic properties in the range 100–300 K using X-ray diffraction (XRD), UV–visible absorption spectroscopy, and PL emission spectroscopy. We observed a phase transition at around 220 K from differential scanning calorimetry (DSC), accompanied by a significant change in the tilt patterns in the inorganic framework, according to structural changes derived from XRD. The linear thermal expansion coefficient along the longest crystal axis is reported for the first time: it does not change between the phases and is similar in magnitude to that of 3D perovskites.^25^ Additionally, a prominent redshift was found in the exciton energy on heating across the structural phase transition, although only a small change in the exciton binding energy between the low- and high-temperature phase (LT and HT, respectively) was observed. A surprisingly strong additional PL peak, emitting below the excitonic resonance, emerged at temperatures below 220 K, which we suggest was caused by radiative emission from defect-bound excitons. Our results highlight the link between structure and optoelectronic properties and are thus important for the development of devices based on layered perovskites.

## Experimental Methods

### Sample Preparation

Single crystals of ThMA_2_PbI_4_ were grown via a slow cooling process. A supersaturated precursor solution (2.12 M) was prepared by dissolving the precursor powders in 100 μL of gamma-butyrolactone at 150 °C. The solution was deposited in between two glass slides at 150 °C, which were cooled to room temperature at a rate of 1 °C/hour. This resulted in the growth of large, plate-like crystals between the glass.

A ThMA_2_PbI_4_ solution (0.4 M) was prepared by dissolving the precursor powders in 1000 μL of a 9:1 DMF:DMSO solvent system at 100 °C. After filtering, half of the resulting stock was diluted in an equivalent volume of 9:1 DMF:DMSO solvent to create a stock of 0.2 M. This solution was deposited onto either glass or FTO-coated glass substrates during a one-step spin-coating process, using 50 μL (glass/FTO) of perovskite solution during an initial 1000 rpm, 10 s loading process, followed by a 5000 rpm, 30 s process. After spin-coating, all samples were left to stand for 20 min at room temperature before a 20 min anneal step at 100 °C. Finally, 30 μL of PMMA (20 mg/mL in chlorobenzene) was deposited on top of the ThMA_2_PbI_4_ film at 1000 rpm for 30 s, which was followed by a 5 s, 2000 rpm process. Thickness measurements of the bare perovskite layers (before PMMA coating) were carried out on a Bruker Dektak XT Stylus Profiler, which determined the thickness to be 100 nm for the 0.2 M films and 200 nm for the 0.4 M films.

Samples on FTO-coated glass were used for temperature-dependent XRD and UV–vis absorption measurements. The sample used for temperature-dependent steady-state PL emission spectroscopy was deposited on uncoated glass instead.

### X-ray Diffraction and Optical Spectroscopies

Single-crystal XRD data were obtained by mounting a suitable crystal on a Rigaku Oxford Diffraction Synergy-S diffractometer with a dual source and equipped with a HyPix-Arc 100 pixel hybrid photon counting X-ray detector. Data were measured using ω scans with Cu K-α radiation (λ = 1.54184 Å). The temperature of the crystal was changed from 100 to 300 K with a step of 25 K. The diffraction pattern was indexed, and the total number of runs and images was based on the strategy calculation from the program *CrysAlisPro*1.171.43.91a.^38^ The structure was solved with the *ShelXT*2018/22^39^ solution program using dual methods and by using *Olex*2–1.53^40^ as the graphical interface. The model was refined with *ShelXL*2019/34^41^ using full matrix least-squares minimization on *F*^2^.

Temperature-dependent thin-film XRD was performed using a Malvern Panalytical Empyrean equipped with a Bragg–Brentano HD mirror giving Mo K-α radiation (0.70932 Å). On the diffracted beam side, the instrument was equipped with a GaliPix 3D detector. An Oxford Cryosystems Phenix stage was used to control the temperature in the range 100–300 K. The sample was mounted on a Monel Alloy 400 holder which had a low thermal expansion over the temperature range. Apiezon N grease was used to create a good thermal contact between the holder and the sample. The sample surface was aligned in the half-cut direct beam at room temperature so that the sample was at the center of rotation of the goniometer. At each temperature, 30 min scans were made in a range of 2–20°. The temperature was ramped at 10 °C/min with a wait time of 30 min to allow for the sample to reach equilibrium. XRD (both thin film and single crystal), UV–vis absorbance, and PL emission temperature-dependent measurements were made on heating.

UV–visible absorbance spectroscopy was measured using a custom-built dual-beam transmission spectrometer scheme that used a quartz tungsten–halogen lamp as a source and an Avantes AvaSpec dual-channel spectrometer to record transmission spectra. Steady-state PL was obtained using a Renishaw Invia spectrometer equipped with a CW 442 nm laser. A Linkam THMS600 stage was used for both temperature-dependent absorption and steady-state PL emission measurements in the 100–300 K range. RS Pro Conductive Lacquer was used to create a good thermal contact between the Linkam stage holder and the sample. The temperature was ramped at 10 °C/min with a wait time of 5 min to allow for the sample to reach equilibrium.

Time-resolved PL spectroscopy was measured using a Horiba DeltaFlex time-correlated single photon counting (TCSPC) lifetime fluorometer, using a fs laser excitation from a Ti:sapphire oscillator (Spectra Physics MaiTai HP, 100 fs pulse width, 80 MHz repetition rate) frequency doubled to 400 nm. The spectral bandpass width was selected to be 9 nm. An Oxford Instruments cryostat (OptistatDN-V) was used to cool the sample to 100 K for the time-resolved PL measurement.

## Results and Discussion

To establish the temperature-dependent structural properties of the layered perovskite ThMA_2_PbI_4_, we performed single-crystal and thin-film XRD measurements, which yielded comprehensive information on the crystal structure and thermal expansion coefficient of the lattice. UV–visible absorption and PL emission spectroscopy at varying temperatures allowed us to quantify the changes in the 1s exciton resonance and other optoelectronic properties across a structural phase transition. A combination of these methods allowed us to study the relationship between the crystal structure and the optoelectronic properties, both within each phase and across the phase transition.

### Structure from XRD on Single Crystals

**Figure 1:**
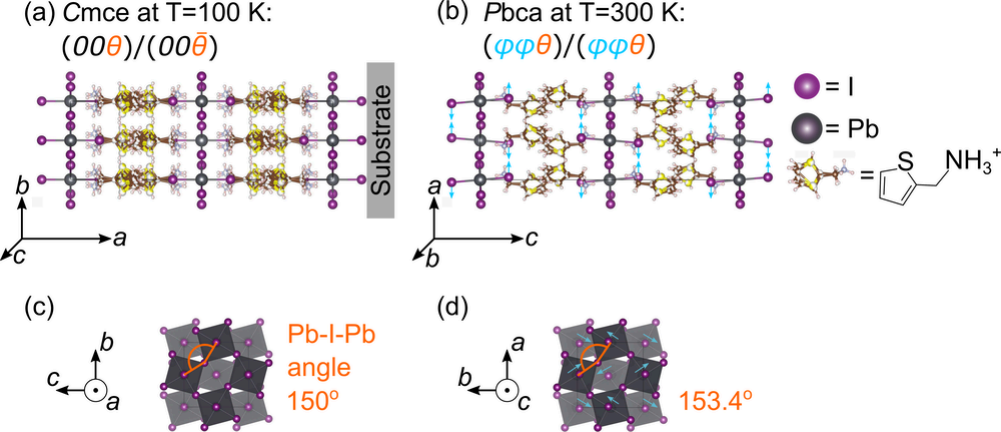
(a), (b) Crystal structure of ThMA_2_PbI_4_ perovskite single crystal at 100 and 300 K, respectively. The thiophene rings portrayed in (a) have two equally likely orientations of the ring. (c), (d) The same crystal structures as viewed along the longest crystallographic axis perpendicular to the inorganic plane, respectively (organic spacers are omitted for clarity in this case). The Pb–I–Pb angle (highlighted by orange) was determined in the metal halide plane and is given at both temperatures. The presence of θ-tilt is manifested by the Pb–I–Pb angle being less than 180° in both phases. The blue arrows in (d) indicate the emergence of the ϕ-tilt pattern in the room temperature phase. The substrate is portrayed in (a), which is relatable to the thin-film samples.

To characterize the structural properties of ThMA_2_PbI_4_, we first performed X-ray diffraction measurements on single-crystal samples at a temperature range from 100 to 300 K in 25 K steps. At 300 K, we found that ThMA_2_PbI_4_ adopts a primitive unit cell with space group *Pbca* (see Figure 1b) and lattice constants *a* = 8.830(3) Å, *b* = 8.763(18) Å, and *c* = 29.08(3) Å, consistent with the literature^37^ and similar to the structure of the RP perovskite phenylmethylammonium lead iodide (PMA_2_PbI_4_).^42^ In this structure, the ThMA cations adopt a conformation where the N–C–C–S torsion angle is approximately 90°. Although not reported previously, we also observed disorder of the ThMA cation with respect to the orientation of the thiophene ring (see Figure S1 in the Supporting Information, SI). To pack between the inorganic layers, the ammonium group of ThMA points toward the acute angle of a parallelogram formed by the bridging iodides in the lead-iodide layer as seen previously for many other Ruddlesden–Popper (RP) structures.^43−46^ Along the *b*-axis, the ammonium cations within a particular organic layer always point to the same direction, and strong hydrogen-bonding interactions promote distortions to the Pb–I lattice (see SI Figure S2). Due to this bonding configuration, neighboring thiophene rings stack in both side-to-face and edge-to-face configurations, forming a zigzag pattern when viewed along the *c*-axis (see SI Figure S2).

Differential scanning calorimetry (DSC) established the presence of a phase transition near 220 K (see SI Figure S3). Accordingly, the LT structure is different from the room temperature structure. At 100 K, the structure from single-crystal XRD had a face-centered orthorhombic unit cell, with space group *Cmce* and lattice constants *a* = 29.0447(8) AA, *b* = 8.6706(2) Å, and *c* = 8.6835(2) Å. A cross-section of the unit cell is shown in Figure 1a. As compared to the high-temperature phase, this structure exhibits increased disorder in the position of the organic cation. Primarily, the amide group can adopt two different positions relative to the lead-iodide octahedra network and to neighboring cations. In both of these positions, the ammonium group is pointed toward the acute angle of the parallelogram formed by the bridging iodides in the lead-iodide layer, as in the high-temperature structure (see SI Figure S1). However, there is no preference in the orientation of the N–C bonds relative to neighboring cations. The N–C–C–S torsion angle can be either negative or positive in the HT phase. As a result, the sulfur atom can adopt either of 4 possible positions. More detailed diagrams of the disorder are provided in the Supporting Information (Figure S1).

Structures that display similar disorder in the orientation of the ammonium group have been reported for both linear and cyclic RP perovskites.^47−49^ For linear cations, it was observed that the interlayer distance in the disordered phase is increased relative to more ordered phases with increased temperature as would be expected.^50^ Additionally, the ammonium group is located farther from the inorganic lattice, which weakens the hydrogen bonding and results in the disordered structure. Although the total volume of the ThMA_2_PbI_4_ unit cell increases as temperature is increased, the length of the longest lattice axis (corresponding to the Pb–I interlayer distance) decreases across the phase change (see SI Figure S4). As a result, the lower-temperature phase has a larger interlayer spacing. Accordingly, we observed a similar increase in the distance between the ammonium group and the inorganic lattice (see SI Figure S5), suggesting similar mechanisms leading to the formation of the more disordered structures. It is also likely that the elongation along the longest lattice axis disrupts weak interactions between the thiophene rings, which further encourages disorder. Interestingly, the structure at low temperature of ThMA_2_PbI_4_ is very similar to structures at higher temperature of Pb–Br and Pb–Cl RP perovskites with similarly sized cyclic cations such as phenylmethylammonium lead bromide, suggesting this structure could be a common feature to smaller unit cells.^48^

Comparing the low-temperature single-crystal data and the room-temperature structure, we also note that the tilting of the metal halide octahedra differs between the phases. The octahedral tilt patterns for *n* = 1 RP layered perovskites have been tabulated recently.^49,51^ To characterize the tilt pattern, the following notation is used: ϕ denotes out-of-phase rotations of adjacent octahedra within the inorganic lattice plane, while θ describes the rotations about the longest lattice axis. According to the previous study,^49^ the *Cmce* space group can adopt a tilting scheme of either  or (*ϕϕ*0)/(*ϕϕ*0), while for *Pbca* it is uniquely (*ϕϕθ*)/(*ϕϕθ*). The two distinct layers in the RP structure can have different tilt patterns, specified within the first and second pair of brackets. We compared these expected tilt patterns to the experimental data for both crystal structures and found that the structure at low temperature *Cmce* exhibits only rotations of the metal halide octahedra about the longest lattice axis and therefore is consistent with the  tilt pattern, as shown in Figure 1c. In contrast, the tilting scheme for the high temperature *Pbca* structure shows both θ-tilts in the metal halide plane and rotations along the longest axis, as highlighted in Figure 1b; therefore, it matches the tilting scheme of (*ϕϕθ*)/(*ϕϕθ*). To quantify the θ-tilt in both phases, we determined the in-plane Pb–I–Pb bond angle of ThMA_2_PbI_4_ to be 150.3° at 100 K (Figure 1c) and 153° at room temperature (Figure 1d). A more detailed analysis of lattice parameters as a function of temperature is provided in the SI (Table S1), as well as enlarged views of the structure at 100 and 300 K (see SI Figure S6).

While changes in octahedral tilt across a phase transition have been reported in other layered perovskites,^52,53^ the different changes occur for the various organic cations. For example, butylammonium lead iodide (BA_2_PbI_4_) and octaethylammonium lead iodide (OA_2_PbI_4_) both exhibit *Pbca* structures at room temperature like ThMA_2_PbI_4_. In BA_2_PbI_4_ single crystals, an order-to-disorder phase transition temperature at 274 K was identified, which was not accompanied by a change in space group.^52,53^ However, this structural change is characterized by increased octahedral tilting in the lower temperature phase as the unit cell size decreases.^52,53^ In contrast to both BA_2_PbI_4_ and ThMA_2_PbI_4_, OA_2_PbI_4_ has a monoclinic *P*2_1_/*a* space group at 100 K, changing to orthorhombic *Pbca* by 298 K.^54^ The *Cmce* structure with  tilt pattern adopted by ThMA_2_PbI_4_ at 100 K is more rarely reported and more often observed in high-temperature phases.^49^ For ThMA_2_PbI_4_, the disorder in the organic cation in the low-temperature phase results in decreased tilting in the Pb–I plane due to the lack of a preferential position for the ammonium group. Therefore, this study and previous work suggest that the chemical nature of the organic spacer controls the crystal lattice and the structures adopted in phases at different temperatures. Previous studies on a range of *n* = 1 layered perovskites have suggested that crystal structures are adopted and structural phase transitions governed by the strength of the hydrogen bonds between the amide groups and halides and the packing of organic cations.^45,52^

The change in octahedral tilt angle has been suggested to be driven by the movement or the reorientation of the organic spacer,^19,55^ and this idea was supported by molecular dynamics simulations showing the increasing penetration depth of the ammonium group into the Pb–I plane above a critical temperature.^56^ The observation that layered perovskites with different ligands exhibit phase transitions at different temperatures (70 K for PEA;^18^ 270 K for BA;^19,53^ and 260 K for octylammonium (OA);^54^ 350 K for hexylammonium (HA);^57^ 320 K for pentylammonium (PA) with an inorganic lattice made of Pb and I) implies this process is influenced by the chemical nature of the cation and its interaction with the inorganic lattice.

### Thermal Expansion from XRD on thin films

**Figure 2:**
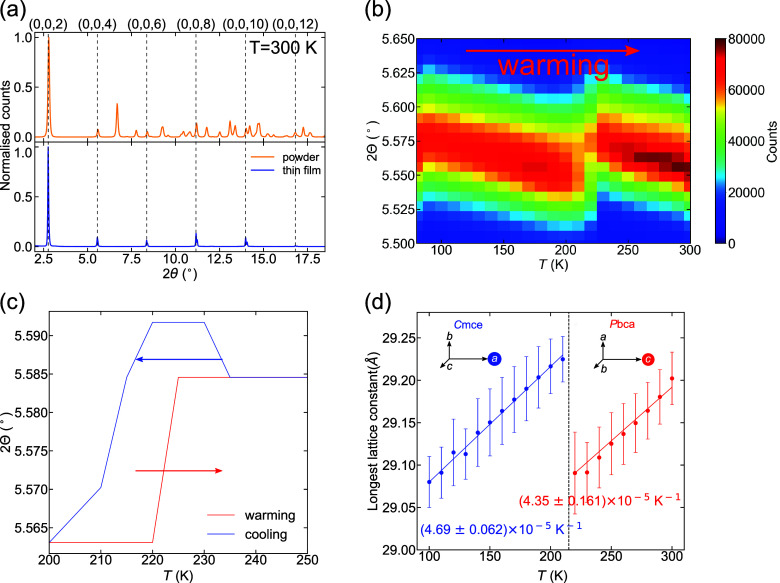
(a) X-ray diffraction pattern of ThMA_2_PbI_4_ powder (modeled from single-crystal measurement) and thin film at *T* = 300 K (as measured). The Bragg peaks corresponding to the respective crystal planes are labeled by dashed lines. (b) Temperature-dependent XRD data of the Bragg peak around the 2θ angle of 5.5° in the 100–300 K temperature range. (c) Temperature dependence of the Bragg peak’s 2θ angle close to the structural phase transition in the temperature range 200–250 K, obtained on cooling and warming (highlighted by arrows). (d) Calculated longest lattice parameter values (*a* for *Cmce* and *c* for *Pbca* space groups, respectively) for each measured temperature of thin film. Solid lines are fits using eq 1, and numbers on the plot show the value of the linear expansion coefficient α, with the dashed line indicating the phase transition temperature.

Although valuable structural information can be deduced from single-crystal XRD, layered perovskites are most often incorporated into devices as thin films. To better approximate device conditions, we therefore used 200 nm thick films of ThMA_2_PbI_4_ deposited on FTO-coated glass and measured their diffractogram in a range of temperatures from 100 to 300 K. The XRD pattern of a thin film at *T* = 300 K is shown in Figure 2a. Six strong Bragg peaks are evident, with a period in 2θ of about 2.8° and with no additional peaks emerging with different periodicity in 2θ. The Bragg peaks were split into two at higher 2θ angles because of the K-α_1_ and K-α_2_ doublet, which became more noticeable at higher angles due to Bragg’s law. Additionally, the intensity ratio of the K-α_2_ and K-α_1_ emission peak was 1:2.^58^ To confirm that the measured Bragg peaks of the thin-film sample belong to the (0, 0, *l*) crystal planes, we used the single-crystal XRD data to model its powder diffraction pattern under the same X-ray wavelength. We used the EXPO2013 toolkit^59^ to model the powder data and matched the Bragg peaks in both data sets that correspond to the (0, 0, *l*) crystal planes at 300 K, as indicated by dashed lines in Figure 2a.

The spacing between lattice planes, *d*_*hkl*_, was calculated from Bragg’s Law, λ = 2*d*_*hkl*_ sin θ_*hkl*_, where θ_*hkl*_ is the diffraction angle for a peak with Miller indices (*h*, *k*, *l*), and λ is the wavelength of the X-ray beam. The following description is valid for the *c* lattice parameter of the *Pbca* room-temperature phase, the long axis of the unit cell. For a Ruddlesden–Popper crystal structure systematic absences mean that for the (0, 0, *l*) family peaks are allowed only when *l* is an even integer. For the low-temperature phase, space group *Cmce*, *a* is the long axis of the unit cell, and hence the peaks are (*h*, 0, 0).

To calculate the *c* lattice constant for ThMA_2_PbI_4_, we first extracted the 2θ angles for each Bragg peak present in the diffractogram by fitting every peak to two Voigt line shape curves with their amplitude ratio being 1:2 to account for the coexistence of K-α_1_ and K-α_2_ emission lines. Then we selected the central position of the Voigt curve corresponding to the K-α_1_ emission line to obtain the 2θ angle. The 2θ angle obtained from the fit in combination with the K-α_1_ wavelength (0.70932 Å) was used in Bragg’s law to get *d*_*hkl*_ for each Bragg peak, with the assignment (0, 0, *l*) for even *l* as in the figure. We then calculated *c* = *ld*_*hkl*_ using the equation for an orthorhombic lattice ( with *h*, *k* = 0). The mean value of the lattice constant was obtained by averaging over the values for the *l* = 2 to *l* = 12 peaks, while error bars show the standard error of the mean. The out-of-plane lattice constant at 300 K for the ThMA_2_PbI_4_ thin films was thus calculated to be *c* = 29.2(2) Å, which matched well with the value of *c* = 29.08(3) Å determined from our single-crystal measurements at 300 K and the value of 29.04 Å reported previously for a single crystal at room temperature.^37^ The different value of *c* of the thin film in comparison to that of the single crystal may indicate residual strain in the thin-film sample due to a mismatch between the thermal expansion coefficient of the substrate and perovskite,^28,30^ as discussed in more detail later in this section.

We recorded diffractograms on heating a ThMA_2_PbI_4_ thin film from 100 to 300 K in 10 K steps (see SI Figure S7). In Figure 2b, we report the behavior of the Bragg peak around 2θ = 5.5° as temperature increases. A rapid, discontinuous increase in the angle of the Bragg peak is evident at *T* = 220 K, which we assign to a structural phase transition from the low-temperature *Cmce* structure (phase I) to the room-temperature *Pbca* (phase II) structure that we observed in single-crystal XRD measurement. Across the structural phase transition (from 210 to 220 K), *c* decreased from 29.22 down to 29.08 Å, which is consistent with an octahedral tilting change from  to a (*ϕϕθ*)/(*ϕϕθ*) tilting scheme, in which the in-plane octahedra tilt angle increases from 150.85° to 153.42° (see SI Table S1), to increase the packing efficiency of the organic cations. The phase transition temperature was found to be hysteretic, i.e., to occur at different temperatures depending on whether the samples were cooled or heated. The Bragg peak around a 2θ angle of 5.5° is again shown at temperatures close to the phase transition in Figure 2c for data obtained on cooling and warming. The phase transition was observed at a slightly higher temperature on heating (220 K) than on cooling (210 K). This temperature-induced phase transition is therefore classified as first-order due to this hysteresis.^60^ Further evidence for a first-order phase transition is the coexistence of phases I and II in the vicinity of the phase transition temperature, as evidenced later in the manuscript using temperature-dependent PL emission spectroscopy. For consistency, we therefore report temperature-dependent data using other characterization methods only on heating the sample.

Within each phase, the Bragg peak’s 2θ angle decreased with increasing temperature. This trend was attributed to the longest unit cell parameter increasing due to thermal expansion, as the Bragg peaks originating from the substrate (FTO peaks) did not shift throughout the temperature-dependent measurement. The thermal expansion coefficient of the perovskite is of substantial interest, as it can be used to evaluate the likelihood of residual stress between the perovskite layer and the substrate^30^ and gives insight into the mechanical stability of perovskite-based devices.^25^ To investigate the thermal expansion coefficient, we analyzed the temperature-induced changes in the longest lattice parameter within each phase, as reported in Figure 2d. The longest lattice parameter is of interest, as it corresponds to the spacing between the inorganic Pb–I layers, and it can be used to quantify the linear thermal expansion coefficient. The longest lattice parameter has a different notation in the two phases: it is *a* in *Cmce*, while it is *c* in the *Pbca* phase. A more detailed explanation on the selection of lattice parameters is given in the SI (Section 3). From now on, we will refer to the longest lattice parameter as *L*. As temperature increased from 100 to 210 K, the parameter *L* increased from 29.07 to 29.22 Å, which can be explained by thermal lattice expansion. In the temperature range from 230 to 300 K, the parameter *L* increased from 29.08 to 29.2 Å, following the lattice expansion trend again.

To quantify the linear thermal expansion coefficient along the interlayer direction, , we examined *L*(*T*) data in each phase (low temperature: *T* < 210 K and high temperature: *T* ≥ 210 K) and fit *L*(*T*) via
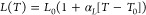
1where *L*_0_ and *T*_0_ are initial values of the lattice constant and temperature, measured at the lowest temperature in each phase, e.g., *L*_0_ = 29.07 Å and *T*_0_ = 100 K in phase I. The temperature-dependent XRD study on thin films was only sensitive to *L*, the long axis of the unit cell, due to the long axis being aligned parallel to the normal of the substrate, and we concentrate on α_*L*_ here. Analysis of the single-crystal data suggests that the linear expansion coefficients in the Pb–I plane are of a similar order of magnitude (see SI Figure S4). Additionally, the linear expansion coefficient is distinct from the volumetric expansion coefficient , where *V* is the volume of the primitive cell.

We found that the linear expansion coefficient was similar in both phases: α_*L*_ = (4.69 ± 0.06) × 10^–5^ K^–1^ for the low-temperature phase and α_*L*_ = (4.35 ± 0.16) × 10^–5^ K^–1^ for the high-temperature phase. While the thermal expansion coefficients of other layered perovskites, to the best of our knowledge, have not been reported yet, a comparison can be made to α for different phases of the 3D perovskites, which were recently reviewed.^61^ For example, the linear expansion coefficients for thin MAPI films were reported to be α_*c*_ = −1.06 × 10^–4^ K^–1^ and α_*a*_ = 1.32 × 10^–4^ K^–1^ for the tetragonal phase and α_*a*_ = 4.77 × 10^–5^ K^–1^ in the cubic phase.^25^ Linear thermal expansion coefficients for other 3D perovskites are reported to be of a similar order of magnitude between 1 × 10^–5^ K^–1^ and 1 × 10^–4^ K^–1^,^61^ which are also consistent with our measurements for ThMA_2_PbI_4_.

We suggest that the α_*L*_ coefficient is of significant importance when layered perovskites are incorporated in multilayered devices, as different layers expanding at a different rate may lead to fractures. The thermal expansion coefficient of the layered perovskite ThMA_2_PbI_4_ is similar to that of MAPI, which allows it to be used as a capping layer for a 3D perovskite to enhance moisture stability^62,63^ without the risk of fracture or mechanical instabilities. However, the typical linear thermal expansion coefficients of substrate materials are much lower, for example 0.37 × 10^–5^ K^–1^, for ITO-coated glass.^31,64^ Therefore, the higher mismatch in thermal expansion coefficients may lead to higher residual strain for layered perovskite films deposited directly onto substrates, as for 3D perovskites.^29,30,32^ Consideration of α is thus vital when optimizing layered perovskites for devices.

### Excitonic Absorption and Exciton Binding Energy

**Figure 3:**
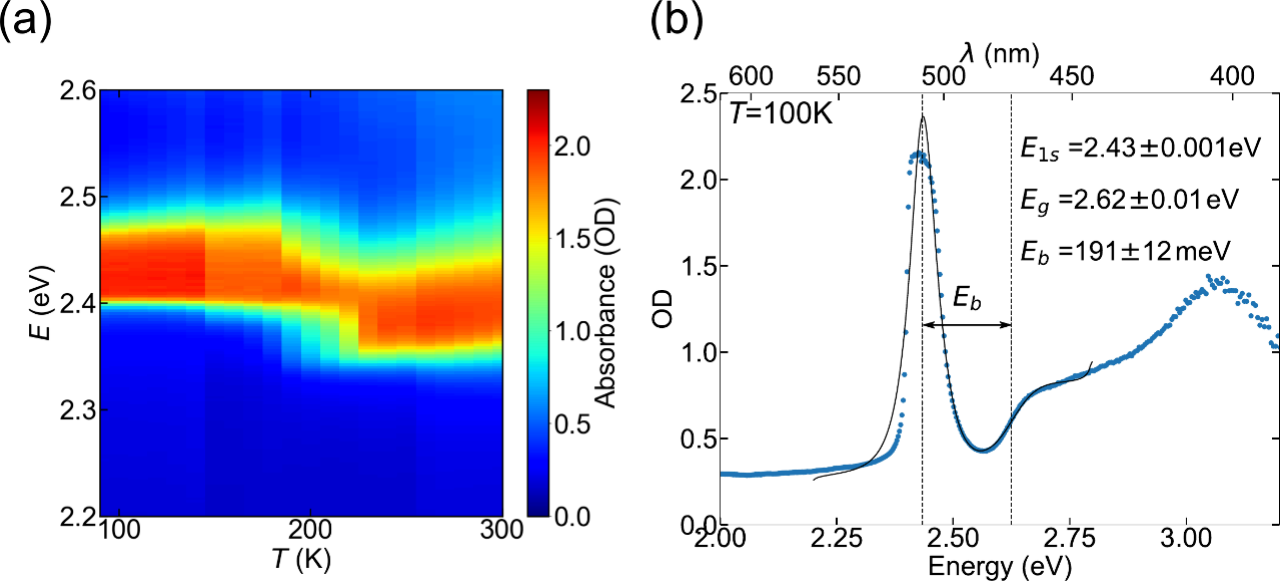
(a) Temperature-dependent absorption spectra near the excitonic ground state *E*_1*s*_ in the 100–300 K temperature range. (b) Absorbance spectrum at 100 K. The dots indicate experimental data, and the solid line shows a fit to the Elliot model.

Besides altering the thermal expansion coefficient, structural phase transitions can also alter the absorption spectrum. The absorption spectrum yields information on parameters that are important for device performance, including the excitonic peak’s position and line width and the exciton binding energy. To investigate how these parameters evolve with temperature and across the structural phase transition, we performed temperature-dependent absorption spectroscopy, with the results shown in the color map in Figure 3a for energies close to the excitonic absorption line and on heating the sample. Temperature-dependent absorption data in a broader range of energies are presented in the SI (Figure S8).

The central energy of the excitonic absorption was 2.44 eV at 100 K and remained relatively constant with temperature in the low-temperature phase. On crossing the structural phase transition at 220 K the excitonic absorption red-shifted to 2.39 eV and then blue-shifted weakly as temperature increased further through the high-temperature phase. To explain this behavior, we recall that the in-plane Pb–I–Pb angle plane is smaller in phase I (150.0° at 100 K) than in phase II (153.4° at RT), indicating a greater θ-tilt angle (Figure 1). A reduction of this Pb–I–Pb in-plane angle increases the single-particle bandgap energy due to a reduced overlap of Pb s- and I p-orbitals^55,65,66^ and hence would blue-shift the PL peak (ignoring any change in the exciton binding energy). Following this argument, we anticipate that the red-shifted excitonic absorption across the structural phase transition (from low to high temperature) results from the in-plane Pb–I–Pb bond angle increasing (octahedral tilt angle decreasing).

To find the exciton binding energy, *E*_*b*_, of the two different phases, we analyzed the absorbance spectra at the lowest (100 K) and highest (300 K) temperatures used in the measurement. An Elliot fit^67^ was used to model the absorption coefficient via α = α_*X*_ + α_*cont*_, where α_*X*_ and α_*cont*_ are the contribution of the excitonic resonances and electron–hole absorption continuum accordingly. The modeling procedure is described in more detail in the SI (Section 5.2) along with the absorption coefficient and complex refractive index at *T* = 100 and 300 K (Figure S9). A representative fit is illustrated in Figure 3b for the absorption spectrum obtained at 100 K, where the exciton binding energy *E*_*b*_ = *E*_*g*_ – *E*_1*s*_ was determined from the difference in the bandgap energy, *E*_*g*_, and the energy of the excitonic peak, *E*_1*s*_. From the absorption spectra we estimated *E*_*b*_ = 191 ± 12 meV at 100 K and *E*_*b*_ = 228 ± 12 meV at 300 K, and thus *E*_*b*_ was marginally larger for phase II. We acknowledge that the absorption spectrum of excitons in layered perovskites can deviate from the conventional hydrogenic Rydberg series of excitonic states and that these deviations may be caused by enhanced dielectric screening.^68,69^ Therefore, we use the estimated exciton binding energy only for comparison with other layered perovskites and do not place much emphasis on the absolute values of the binding energy.

For other *n* = 1 RP layered perovskites, *E*_*b*_ is in the range 200–400 meV at room temperature,^69−71^ consistent with our *E*_*b*_ for ThMA_2_PbI_4_. *E*_*b*_ was found to increase with temperature for BA_2_PbI_4_ from 420 meV at 100 K to 490 meV at 300 K using temperature-dependent two-photon PL excitation spectroscopy,^19^ although no substantial shift in *E*_*b*_ across a phase transition at 265 K was found despite a substantial change in *E*_1*s*_. Similarly, a study on PEA_2_PbI_4_ reported that the exciton binding energy did not show any significant change with temperature.^72^ However, the exciton binding energy in 3D perovskites has been studied using magneto-transmission at high magnetic fields, and it has been shown to continuously decrease as temperature increases.^73^ This is in contrast to our result for ThMA_2_PbI_4_, where *E*_*b*_ marginally increases with temperature. At this juncture there is no consensus on whether or not *E*_*b*_ should vary substantially with temperature or across a structural phase transition for layered perovskites: likely, it depends on the host of factors that influence the single-particle bandstructure and the strength of carrier–carrier interactions.

### Photoluminescence

**Figure 4:**
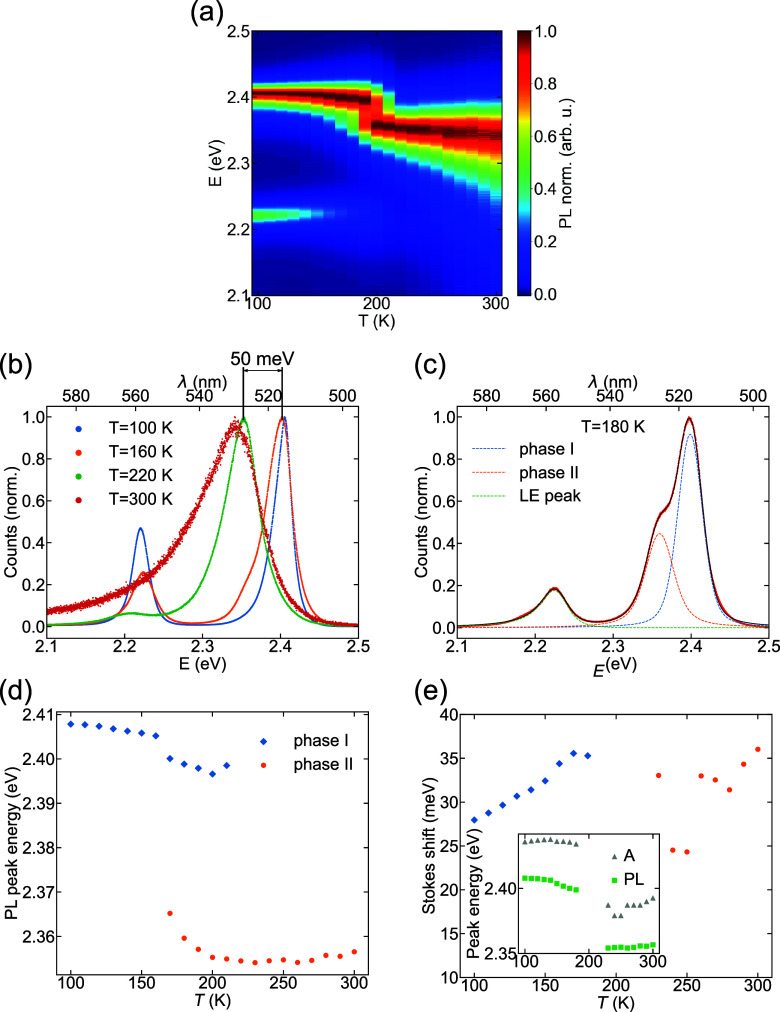
(a) Temperature-dependent PL data presented as a contour map (normalized). (b) Photoluminescence (PL) spectra at 100, 160, 220, and 300 K (normalized). (c) Three peak model used to fit PL spectrum at 180 K. (d) Temperature-dependent peak position of the corresponding peaks in PL emission spectra, extracted from fit. (e) Temperature-dependent Stokes shift energy, obtained by subtracting the peak energy of absorption and PL emission spectrum. The inset shows peak energies obtained from both absorption (A) and PL measurements in both phases (temperature range where two phases coexist is not included).

PL emission analysis is a widely used characterization tool for semiconductor devices, and it is applicable for low-dimensional semiconductors to analyze processes such as carrier recombination mechanisms^74^ and the luminescence of impurities.^75^ PL spectra measured at different temperatures allow one to investigate the defects linked to nonradiative processes^76,77^ and localized states,^78^ which are critical for the understanding of optoelectronic properties across the phase transition. To study the impact of the structural phase transition on the PL emission properties, we performed temperature-dependent photoluminescence (PL) measurements in the 100–300 K temperature range on heating the sample. Normalized PL emission spectra are shown in the contour map in Figure 4a, while lineshapes at 100, 160, 220, and 300 K are shown in Figure 4b. The PL spectrum peaked at 2.4 eV at 100 K, and it rapidly red-shifted by about 50 meV across the structural phase transition. The PL peak energy did not change rapidly with temperature in either the low- or the high-temperature phases, consistent with the absorption spectra. However, the PL line shape broadened asymmetrically with increasing temperature, developing a tail at lower energies. A similar asymmetric PL emission line shape has been reported previously in other layered perovskites, and its origin is still under debate. Possible explanations include strong exciton–phonon coupling,^79^ self-trapped excitons,^80−82^ or self-absorption effects in the perovskite film.^83^ Further, we observed an additional PL peak at 2.22 eV, which emerged at low temperatures *T* < 220 K and which was red-shifted by about 200 meV with respect to the main peak in the PL emission spectrum. Below, we refer to this peak as the low energy (LE) peak and the main peaks from each phase, caused by the radiative recombination of excitons, as the X peaks.

The LE peak is evident in Figure 4a–c at around 2.22 eV and occurs most noticeably in phase I simultaneously with the X peak. We note that the occurrence of strong dual PL peaks has previously been reported in the Ruddlesden–Popper layered perovskites BA_2_PbI_4_, OA_2_PbI_4_, and PEA_2_PbI_4_^53,54,84^ and has been suggested to originate from the simultaneous presence of a surface phase with a different structure or composition.^53,54^ However, here we can rule out the presence of any such additional parasitic phase: no extra phase was observed in XRD, and the UV–visible absorption does not show any excitonic absorption features at 2.2 eV. We rule out the possibility that the LE feature is PL from self-trapped excitons because a high Stokes shift (several times larger than the binding energy *E*_*b*_) and broad PL resonance are characteristics of the self-trapped exciton,^85^ and these features are not evident here. Emission from biexcitons is a possible mechanism for the LE PL peak, as it produces a peak red-shifted from the excitonic PL peak. PL emission from the radiative recombination of biexcitons can be identified via its quadratic dependence on excitation power.^86,87^ As seen in the SI (Figure S10), the intensity dependence of the LE PL peak has a sublinear trend, suggesting that its origin is not biexcitonic.

The emergence of additional PL peaks below the bandgap has also been observed in two-dimensional inorganic semiconductors enriched with defects, such as MoS_2_^88,89^ and WSe_2_,^90^ and were assigned to radiative emission from excitons bound to chalcogen vacancies. In MoS_2_ for example, the defect-bound exciton peaks are shifted by 200 meV from the main PL peak and are more prominent at low temperature.^88^ We therefore suggest that the LE peak corresponds to a defect-bound exciton: PL emission can occur from excitons bound to defects, without producing prominent absorption at that energy. This assignment is further supported by intensity dependent measurements in which the trend for the LE peak exhibits a sublinear dependence at higher powers (see SI Figure S10), consistent with the power dependence for defect-bound excitons.^91^ It has been suggested that in-plane iodine vacancies in layered perovskites produce electron traps, creating emission peaks below bandgap, as obtained from first-principles DFT calculations.^82,92^ The iodine vacancy is thus a potential candidate for the defect emission observed in this study, but further work is needed to reach a conclusive assignment.

Two peaks were also observed in the PL spectra closely spaced around 2.4 eV for a temperature range of 150–220 K (see spectra at 180 K in Figure 4c). In contrast to the observation of the defect-bound exciton peak at low temperature, the two peaks here indicate the coexistence of phases around the phase transition temperature, as is expected for a first-order phase transition.^60^ The first-order phase transition in layered perovskites was demonstrated previously by showing the hysteresis in PL emission spectroscopy for BA_2_PbI_4_ and OA_2_PbI_4_,^53,54^ as well as for 3D perovskites, e.g., MAPI.^93^ To accurately fit the PL spectra at different temperatures, including the phase coexistence range (150–220 K) and LE peak, three Voigt functions were used. The PL spectrum around 2.4 eV, caused by exciton recombination, was fitted using two peaks to account for coexistence of phases I and II, and an additional Voigt function was used to model the LE peak. Voigt functions were used as they account for homogeneous and inhomogeneous broadening and could match the line shape more accurately than either Lorentzian or Gaussian resonances. Only one skewed Voigt function was sufficient to fit the data at *T* > 230 K, as the LE peak and the X peak corresponding to phase I in Figure 4b became absent; using a skewed Voigt function to account for the PL line shape becoming increasingly asymmetrical.

Fitting the PL data allowed the accurate extraction of the temperature dependence of the central energy, as shown for the X peaks in both structural phases in Figure 4d. A rapid red-shift of PL peak energy by around 50 meV is evident when transitioning from phase I to phase II, matching the results from UV–visible absorption spectroscopy (Figure 3). We therefore similarly attribute the red-shift in the PL peak across the structural phase transition (from low to high temperature) to the increase in the in-plane Pb–I–Pb bond angle (decrease in octahedral tilt angle). A similar redshift in the PL peak energy was observed for the order-to-disorder phase transition at 275 K in BA_2_PbI_4_ and was also assigned to an increase in the Pb–I–Pb bond angle.^53,79^

To gain further insight into the temperature-dependent optoelectronic properties, we analyzed the temperature-dependent Stokes shift Δ = *E*_1*s*,abs_ – *E*_1*s*,PL_. The Stokes shift is caused by fluctuations in the exciton energy, such as the presence of structural disorder (e.g., from surface states or defects), which allows exciton localization into lower energy states before recombining.^94^ Δ was reliably extracted from the 1s exciton peak energies measured in the absorbance and PL spectra (Figure 4e) at temperatures outside the phase coexistence temperature range (180–220 K). The Stokes shift Δ showed a steady increase from 28 meV at 100 K to 36 meV at 180 K in the low-temperature phase, while no clear trend was seen in the high-temperature phase. This was due to the difficulty in distinguishing the interband absorption when the excitonic absorption peak is broader, as was especially noticeable at 230–250 K (inset of Figure 4e). Similar temperature-dependent Stokes shifts to that of phase I have been observed in 3D perovskites CsPbBr_3_ and MAPbBr_3_: increasing from 10 to 15 meV at 100 K up to 40 meV at 200 K.^95^ Layered perovskite PEA_2_PbI_4_ thin films studied previously showed a Stokes shift Δ = 25 meV at 100 K that increased weakly with temperature.^96^ Hence the observed change in the Stokes shift of ThMA_2_PbI_4_ with temperature was similar to that of other perovskites, in the range of a few tens of meV.

**Figure 5:**
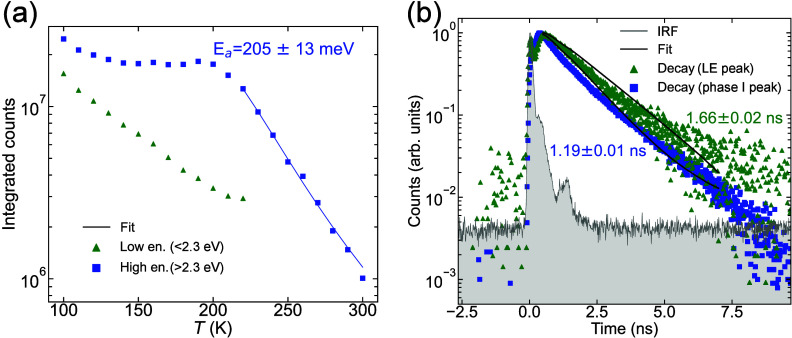
(a) Integrated PL counts in both spectral regions (<2.3 eV, green and >2.3 eV, blue) with Arrhenius fit for the X peak (solid line). (b) TCSPC decay curves of the LE (blue) and the X (green) peaks measured at low temperature (100 K). Solid lines show single-exponential fits. The gray line is the instrument response function (IRF).

Alternatively, we analyzed the PL spectra by dividing the spectra into two parts: low (*E* < 2.3 eV) and high energy (*E* > 2.3 eV) regions, to separate the LE peak from the X peaks. We integrated the PL counts in these two spectral regions at each temperature, as reported in Figure 5a. The integrated intensity in phase II was compared with the predictions of the Arrhenius model:^97^
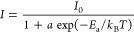
2where *I*_0_ is the anticipated PL intensity at 0 K, where nonradiative decay is expected to be negligible; *a* is the ratio of radiative lifetime τ_R_ and total lifetime τ_0_; *E*_a_ is the activation energy; *k*_B_ is the Boltzmann constant; and *T* is the temperature. With this method the activation energy of the X peak was estimated to be about 200 meV in the 230–300 K temperature range, close to the exciton binding energy *E*_*b*_ = 228 ± 12 meV obtained from the Elliott fit. While several studies have used the Arrhenius method to estimate the exciton binding energy in 3D^98,99^ and layered perovskites,^100^ there are factors that limit its validity, and it should only be used with caution. The Arrhenius analysis of the temperature-dependent PL data assumes that the quenching of the PL is caused by thermally activated exciton recombination only,^101^ where the nonradiative lifetime is expressed as  and the radiative lifetime τ_R_ is considered to be independent of temperature in eq 2.^97^ This assumption should be carefully considered, as it has been shown that the radiative recombination rate in 3D perovskites varies with temperature.^20^ In our specific case with multiple PL peaks in phase I, there are two possible recombination channels that compete with each other at temperatures below 230 K, and eq 2 cannot be used.

Finally, we investigated the transient behavior of the X and LE PL peaks seen in phase I to gain further insight into their origin. We measured the lifetime of the LE and X peaks at 100 K using time-correlated single photon counting (TCSPC), as presented in Figure 5b. A slower decay dynamic can be observed for the LE PL peak in comparison to the main PL peak. The apparent features near time zero are within the instrument response time and are therefore artifacts of the spectrometer. The higher background signal for the LE peak in comparison to the X peak is a result of the longer acquisition time required for this measurement due to its lower count rate. The lifetime (obtained using a single-exponential fit) of the X peak was 1.19 ± 0.01 ns, while for the LE peak it was 1.66 ± 0.02 ns. The LE peak thus exhibited a 40% longer lifetime than the X peak, consistent with expectations for defect-bound states. For example, defect-bound excitons in monolayers of MoS_2_^102^ and WSe_2_^90^ have been shown to have increased lifetime in comparison with the interband exciton. The longer lifetime of the LE feature and its absence in the absorption spectrum suggest that it originates from radiative emission from defect-bound excitons.

## Conclusions

In conclusion, we discovered and characterized a first-order structural phase transition at 220 K in the layered perovskite ThMA_2_PbI_4_, which we studied using temperature-dependent X-ray diffraction, absorbance, and photoluminescence spectroscopy. In the room-temperature phase, the structure had space group *Pbca*, with tilt pattern (*ϕϕθ*)/(*ϕϕθ*), while the lower-temperature phase had *Cmce* and . We observed a rapid decrease in *c* lattice parameter on heating across the phase transition, and we determined the thermal expansion coefficient of the layered perovskite ThMA_2_PbI_4_ for the first time, which did not change significantly across the phase transition. The thermal expansion coefficient was similar to that of the 3D metal halide perovskite, cubic MAPI, which enables ThMA_2_PbI_4_ to be used as a capping layer for 3D perovskites.

The structural phase transition affected the optoelectronic properties: the excitonic resonance red-shifted on crossing the structural phase transition from lower to higher temperature. The mechanism of the structural phase transition was attributed to the reorientation of organic spacers, which changed the tilt angle of the inorganic octahedra and the interlayer spacing simultaneously. The altered tilt angle modified the overlap of Pb and I atomic orbitals and changed the electronic bandstructure, which modified the aforementioned optoelectronic properties. Furthermore, we observed an additional, narrow photoluminescence peak emerging at temperatures below 220 K, which was red-shifted with respect to the interband excitonic peak by 200 meV in its emission spectrum and attributed its origin to radiative emission from defect-bound excitons.

These findings contribute to a better understanding of the structure and optoelectronic properties related to the interaction between the organic cation and lead halide layer, helping to develop strategies that alter functional properties in a desirable way. Future studies could focus on the quantitative analysis of the influence of film thickness and/or strain on the temperature of the structural phase transition and the hysteresis window. Strain management in perovskite thin films is of substantial interest, as it might control the formation of defects, in turn enhancing or preventing the radiative emission from defect-bound states. Further analysis to better understand the nature of the light-active defect present in this layered perovskite may be beneficial, as the PL emission is relatively strong in comparison to the main interband excitonic peak and hence could be used in two-color light-emitting applications. The ability to change PL emission properties by altering temperature could be applied in perovskite lasers^103,104^ and light emitters.^8,9^ Finally, the methodology established in this study will be an invaluable starting point to further advance our knowledge of the optoelectronic properties and thermal stability of layered perovskites with different organic ligands.

## Supporting Information Available

Crystal structures for ThMA_2_PbI_4_ in the temperature range 100–300 K are also available (ZIP)
Thin-film XRD data in the temperature range 80–300 K; optical density of ThMA_2_PbI_4_ thin films in the temperature range 90–300 K; and details of the Elliott fit model and fit parameters at 100 and 300 K temperatures (PDF)
