# Supplementary material for: Temperature-Dependent Structural and Optoelectronic Properties of the Layered Perovskite 2-Thiophenemethylammonium Lead Iodide
Source: J Phys Chem C Nanomater Interfaces. 2024 Jul 25;128(31):13108–20. doi: 10.1021/acs.jpcc.4c03221 (PMC11317984; doi:10.1021/acs.jpcc.4c03221)
Supplement: Supplementary file 1 — jp4c03221_si_001.zip [file jp4c03221_si_001.zip › ThMA2PbI4_Temp-depSCXRD/datareport_100k.docx]

**ThMA2PbI4_1_100**

| **Table 1 Crystal data and structure refinement for ThMA2PbI4_1_100.** | |
| --- | --- |
| Identification code | ThMA2PbI4_1_100 |
| Empirical formula | C_20_H_64_I_8_N_4_Pb_2_S_4_ |
| Formula weight | 1918.57 |
| Temperature/K | 99.99(10) |
| Crystal system | orthorhombic |
| Space group | Cmce |
| a/Å | 29.0447(8) |
| b/Å | 8.6706(2) |
| c/Å | 8.6835(2) |
| α/° | 90 |
| β/° | 90 |
| γ/° | 90 |
| Volume/Å^3^ | 2186.81(9) |
| Z | 2 |
| ρ_calc_g/cm^3^ | 2.914 |
| μ/mm^‑1^ | 13.546 |
| F(000) | 1728.0 |
| Crystal size/mm^3^ | 0.1 × 0.08 × 0.02 |
| Radiation | Mo Kα (λ = 0.71073) |
| 2Θ range for data collection/° | 5.61 to 58.216 |
| Index ranges | -39 ≤ h ≤ 39, -11 ≤ k ≤ 11, -11 ≤ l ≤ 11 |
| Reflections collected | 10426 |
| Independent reflections | 1500 [R_int_ = 0.0385, R_sigma_ = 0.0239] |
| Data/restraints/parameters | 1500/336/134 |
| Goodness-of-fit on F^2^ | 1.363 |
| Final R indexes [I>=2σ (I)] | R_1_ = 0.0402, wR_2_ = 0.0881 |
| Final R indexes [all data] | R_1_ = 0.0418, wR_2_ = 0.0885 |
| Largest diff. peak/hole / e Å^-3^ | 2.50/-2.89 |

| **Table 2 Fractional Atomic Coordinates (×10^4^) and Equivalent Isotropic Displacement Parameters (Å^2^×10^3^) for ThMA2PbI4_1_100. U_eq_ is defined as 1/3 of the trace of the orthogonalised U_IJ_ tensor.** | | | | |
| --- | --- | --- | --- | --- |
| **Atom** | ***x*** | ***y*** | ***z*** | **U(eq)** |
| Pb01 | 5000 | 5000 | -5000 | 14.26(16) |
| I002 | 5000 | 6828.2(9) | -1830.9(9) | 17.67(19) |
| I003 | 6100.4(3) | 5000 | -5000 | 28.5(2) |
| N7 | 5940(30) | 5750(80) | 880(80) | 23(5) |
| C5 | 6669(9) | 5040(30) | -160(40) | 36(5) |
| C2 | 7496(9) | 4330(40) | -20(70) | 23(6) |
| C6A | 6172(11) | 5040(70) | -410(50) | 31(4) |
| C4 | 6961(9) | 6250(50) | -340(70) | 33(7) |
| C3 | 7411(10) | 5870(40) | -160(60) | 27(6) |
| S1 | 6977(4) | 3375(13) | 230(20) | 40(4) |
| C5A | 6681(9) | 5040(50) | 80(30) | 37(5) |
| C2A | 7486(9) | 5250(60) | -680(40) | 25(7) |
| C4A | 6980(9) | 4930(60) | 1270(40) | 34(6) |
| C3A | 7430(10) | 5030(50) | 860(40) | 20(6) |
| S1A | 6965(4) | 5350(20) | -1652(13) | 40(3) |
| C6 | 6159(11) | 4590(50) | -150(70) | 31(4) |
| N7A | 5920(30) | 5900(90) | 800(90) | 23(5) |

| **Table 3 Anisotropic Displacement Parameters (Å^2^×10^3^) for ThMA2PbI4_1_100. The Anisotropic displacement factor exponent takes the form: -2π^2^[h^2^a*^2^U_11_+2hka*b*U_12_+…].** | | | | | | |
| --- | --- | --- | --- | --- | --- | --- |
| **Atom** | **U_11_** | **U_22_** | **U_33_** | **U_23_** | **U_13_** | **U_12_** |
| Pb01 | 16.7(3) | 13.7(3) | 12.3(3) | -0.2(2) | 0 | 0 |
| I002 | 23.8(4) | 15.4(3) | 13.8(3) | -4.1(3) | 0 | 0 |
| I003 | 16.9(4) | 35.6(5) | 32.9(5) | 1.0(4) | 0 | 0 |
| N7 | 17(7) | 25(11) | 25(9) | 6(7) | -3(7) | -1(7) |
| C5 | 18(6) | 52(10) | 38(11) | 8(9) | -3(9) | 5(8) |
| C2 | 10(9) | 34(11) | 25(15) | 2(11) | 4(12) | 0(9) |
| C6A | 20(5) | 41(9) | 30(9) | 0(8) | -2(6) | 3(7) |
| C4 | 18(9) | 47(12) | 34(15) | -3(12) | 1(11) | 7(8) |
| C3 | 18(8) | 35(11) | 27(15) | 4(10) | 3(11) | 3(9) |
| S1 | 24(5) | 24(5) | 71(9) | 0(6) | 2(5) | -1(4) |
| C5A | 19(5) | 54(10) | 37(10) | 8(9) | 0(8) | 3(10) |
| C2A | 15(10) | 39(19) | 21(11) | -5(11) | -5(9) | -5(12) |
| C4A | 19(8) | 49(15) | 33(11) | 1(12) | 2(8) | 5(11) |
| C3A | 18(8) | 21(15) | 22(11) | -5(11) | -2(9) | 7(11) |
| S1A | 23(5) | 75(9) | 21(5) | 5(5) | -3(4) | 5(5) |
| C6 | 20(5) | 41(9) | 30(9) | 0(8) | -2(6) | 3(7) |
| N7A | 17(7) | 25(11) | 25(9) | 6(7) | -3(7) | -1(7) |

| **Table 4 Bond Lengths for ThMA2PbI4_1_100.** | | | | | | |
| --- | --- | --- | --- | --- | --- | --- |
| **Atom** | **Atom** | **Length/Å** |  | **Atom** | **Atom** | **Length/Å** |
| Pb01 | I002 | 3.1758(7) |  | C2 | C3 | 1.359(17) |
| Pb01 | I002^1^ | 3.1758(7) |  | C2 | S1 | 1.73(2) |
| Pb01 | I002^2^ | 3.1766(7) |  | C6A | C5A | 1.534(15) |
| Pb01 | I002^3^ | 3.1766(7) |  | C6A | N7A | 1.484(16) |
| Pb01 | I003^1^ | 3.1961(8) |  | C4 | C3 | 1.360(17) |
| Pb01 | I003 | 3.1961(8) |  | C5A | C4A | 1.359(17) |
| N7 | C6 | 1.485(15) |  | C5A | S1A | 1.73(2) |
| C5 | C4 | 1.359(17) |  | C2A | C3A | 1.359(17) |
| C5 | S1 | 1.73(2) |  | C2A | S1A | 1.73(2) |
| C5 | C6 | 1.533(15) |  | C4A | C3A | 1.360(17) |

^1^1-X,1-Y,-1-Z; ^2^+X,-1/2+Y,-1/2-Z; ^3^1-X,3/2-Y,-1/2+Z

| **Table 5 Bond Angles for ThMA2PbI4_1_100.** | | | | | | | | |
| --- | --- | --- | --- | --- | --- | --- | --- | --- |
| **Atom** | **Atom** | **Atom** | **Angle/˚** |  | **Atom** | **Atom** | **Atom** | **Angle/˚** |
| I002 | Pb01 | I002^1^ | 180.0 |  | C4 | C5 | S1 | 110(2) |
| I002^1^ | Pb01 | I002^2^ | 90.089(7) |  | C4 | C5 | C6 | 143(3) |
| I002 | Pb01 | I002^2^ | 89.911(7) |  | C6 | C5 | S1 | 107(3) |
| I002^1^ | Pb01 | I002^3^ | 89.911(7) |  | C3 | C2 | S1 | 109(3) |
| I002 | Pb01 | I002^3^ | 90.089(7) |  | N7A | C6A | C5A | 106(4) |
| I002^2^ | Pb01 | I002^3^ | 180.000(14) |  | C5 | C4 | C3 | 113(4) |
| I002 | Pb01 | I003^1^ | 90.0 |  | C2 | C3 | C4 | 115(4) |
| I002^2^ | Pb01 | I003^1^ | 90.0 |  | C5 | S1 | C2 | 91.4(17) |
| I002^1^ | Pb01 | I003 | 90.0 |  | C6A | C5A | S1A | 103(2) |
| I002^3^ | Pb01 | I003^1^ | 90.0 |  | C4A | C5A | C6A | 145(3) |
| I002^3^ | Pb01 | I003 | 90.0 |  | C4A | C5A | S1A | 112(2) |
| I002^1^ | Pb01 | I003^1^ | 90.0 |  | C3A | C2A | S1A | 112(3) |
| I002^2^ | Pb01 | I003 | 90.0 |  | C3A | C4A | C5A | 114(3) |
| I002 | Pb01 | I003 | 90.0 |  | C2A | C3A | C4A | 113(4) |
| I003^1^ | Pb01 | I003 | 180.0 |  | C2A | S1A | C5A | 89.3(17) |
| Pb01 | I002 | Pb01^4^ | 149.98(3) |  | N7 | C6 | C5 | 104(4) |

^1^1-X,1-Y,-1-Z; ^2^+X,-1/2+Y,-1/2-Z; ^3^1-X,3/2-Y,-1/2+Z; ^4^1-X,3/2-Y,1/2+Z

| **Table 6 Torsion Angles for ThMA2PbI4_1_100.** | | | | | | | | | | |
| --- | --- | --- | --- | --- | --- | --- | --- | --- | --- | --- |
| **A** | **B** | **C** | **D** | **Angle/˚** |  | **A** | **B** | **C** | **D** | **Angle/˚** |
| C5 | C4 | C3 | C2 | 7(5) |  | C5A | C4A | C3A | C2A | 0(4) |
| C6A | C5A | C4A | C3A | 179.7(16) |  | C4A | C5A | S1A | C2A | 2(2) |
| C6A | C5A | S1A | C2A | -178.9(11) |  | C3A | C2A | S1A | C5A | -2(2) |
| C4 | C5 | S1 | C2 | -5(3) |  | S1A | C5A | C4A | C3A | -2(3) |
| C4 | C5 | C6 | N7 | -48(5) |  | S1A | C2A | C3A | C4A | 1(4) |
| C3 | C2 | S1 | C5 | 9(3) |  | C6 | C5 | C4 | C3 | 178(3) |
| S1 | C5 | C4 | C3 | 0(4) |  | C6 | C5 | S1 | C2 | 176.4(19) |
| S1 | C5 | C6 | N7 | 130(5) |  | N7A | C6A | C5A | C4A | 39(5) |
| S1 | C2 | C3 | C4 | -10(4) |  | N7A | C6A | C5A | S1A | -139(5) |

| **Table 7 Hydrogen Atom Coordinates (Å×10^4^) and Isotropic Displacement Parameters (Å^2^×10^3^) for ThMA2PbI4_1_100.** | | | | |
| --- | --- | --- | --- | --- |
| **Atom** | ***x*** | ***y*** | ***z*** | **U(eq)** |
| H7A | 6091.17 | 6666.94 | 791.96 | 27 |
| H7B | 5951.95 | 5418.92 | 1868.46 | 27 |
| H7C | 5639.97 | 5883.58 | 593.91 | 27 |
| H2 | 7790.85 | 3862.15 | -46.78 | 28 |
| H6AA | 6133.81 | 5545.83 | -1419.32 | 37 |
| H6AB | 6056.1 | 3966.7 | -482.76 | 37 |
| H4 | 6859.49 | 7271.38 | -563.36 | 40 |
| H3 | 7650.14 | 6617.5 | -145.8 | 32 |
| H2A | 7777.38 | 5330.97 | -1171.45 | 30 |
| H4A | 6882.86 | 4784.47 | 2309.46 | 40 |
| H3A | 7678.1 | 4967.52 | 1565.25 | 24 |
| H6A | 6026.65 | 4640.99 | -1196.71 | 37 |
| H6B | 6116.65 | 3538.43 | 265.87 | 37 |
| H7AA | 6084.73 | 5882.58 | 1694.08 | 27 |
| H7AB | 5640.59 | 5446.09 | 964.89 | 27 |
| H7AC | 5877.57 | 6890.61 | 496 | 27 |

| **Table 8 Atomic Occupancy for ThMA2PbI4_1_100.** | | | | | | | |
| --- | --- | --- | --- | --- | --- | --- | --- |
| **Atom** | ***Occupancy*** |  | **Atom** | ***Occupancy*** |  | **Atom** | ***Occupancy*** |
| N7 | 0.252(6) |  | H7A | 0.503(11) |  | H7B | 0.503(11) |
| H7C | 0.503(11) |  | C5 | 0.252(6) |  | C2 | 0.252(6) |
| H2 | 0.503(11) |  | C6A | 0.248(6) |  | H6AA | 0.497(11) |
| H6AB | 0.497(11) |  | C4 | 0.252(6) |  | H4 | 0.503(11) |
| C3 | 0.252(6) |  | H3 | 0.503(11) |  | S1 | 0.252(6) |
| C5A | 0.248(6) |  | C2A | 0.248(6) |  | H2A | 0.497(11) |
| C4A | 0.248(6) |  | H4A | 0.497(11) |  | C3A | 0.248(6) |
| H3A | 0.497(11) |  | S1A | 0.248(6) |  | C6 | 0.252(6) |
| H6A | 0.503(11) |  | H6B | 0.503(11) |  | N7A | 0.248(6) |
| H7AA | 0.497(11) |  | H7AB | 0.497(11) |  | H7AC | 0.497(11) |

**Experimental**

Single crystals of C_20_H_64_I_8_N_4_Pb_2_S_4_ **[ThMA2PbI4_1_100]** were **[]**. A suitable crystal was selected and **[]** on a **XtaLAB Synergy, Dualflex, HyPix-Arc 100** diffractometer. The crystal was kept at 99.99(10) K during data collection. Using Olex2 [1], the structure was solved with the SHELXT [2] structure solution program using Intrinsic Phasing and refined with the SHELXL [3] refinement package using Least Squares minimisation.

1. Dolomanov, O.V., Bourhis, L.J., Gildea, R.J, Howard, J.A.K. & Puschmann, H. (2009), J. Appl. Cryst. 42, 339-341.
2. Sheldrick, G.M. (2015). Acta Cryst. A71, 3-8.
3. Sheldrick, G.M. (2015). Acta Cryst. C71, 3-8.

**Crystal structure determination of [ThMA2PbI4_1_100]**

**Crystal Data** for C_20_H_64_I_8_N_4_Pb_2_S_4_ (*M*=1918.57 g/mol): orthorhombic, space group Cmce (no. 64), *a* = 29.0447(8) Å, *b* = 8.6706(2) Å, *c* = 8.6835(2) Å, *V*= 2186.81(9) Å^3^, *Z* = 2, *T* = 99.99(10) K, μ(Mo Kα) = 13.546 mm^-1^, *Dcalc* = 2.914 g/cm^3^, 10426 reflections measured (5.61° ≤ 2Θ ≤ 58.216°), 1500 unique (*R*_int_ = 0.0385, R_sigma_ = 0.0239) which were used in all calculations. The final *R*_1_ was 0.0402 (I > 2σ(I)) and *wR*_2_ was 0.0885 (all data).

**Refinement model description**

Number of restraints - 336, number of constraints - unknown.

Details:

1. Fixed Uiso
 At 1.2 times of:
 All C(H) groups, All C(H,H) groups, All N(H,H,H) groups
2. Restrained distances
 C6A-C5A = C6-C5
 1.54 with sigma of 0.02
 N7-C6 = N7A-C6A
 1.48 with sigma of 0.02
 N7A-C6A ≈ N7-C6
 with sigma of 0.02
 C6A-C5A ≈ C6-C5
 with sigma of 0.02
 S1-C2 ≈ S1-C5 ≈ S1A-C2A ≈ S1A-C5A
 with sigma of 0.002
 C5A-C4A ≈ C4A-C3A ≈ C3A-C2A ≈ C3-C2 ≈ C4-C3 ≈ C5-C4
 with sigma of 0.002
3. Restrained planarity
 C6A, C5A, S1A, C2A, C3A, C4A
 with sigma of 0.1
 C6, C5, C4, C3, C2, S1
 with sigma of 0.1
4. Uiso/Uaniso restraints and constraints
All non-hydrogen atoms have similar U: within 2A with sigma of 0.04 and sigma
for terminal atoms of 0.08 within 2A
C6A ≈ C6 ≈ C5A ≈ C5: within 2A with sigma of 0.02 and sigma for
terminal atoms of 0.04 within 2A
N7 ≈ N7A ≈ C5A ≈ C5 ≈ C6A ≈ C6: within 2A with sigma of
0.02 and sigma for terminal atoms of 0.04 within 2A
Uanis(C6A) = Uanis(C6)
Uanis(N7A) = Uanis(N7)
5. Rigid body (RIGU) restrains
 All non-hydrogen atoms
 with sigma for 1-2 distances of 0.004 and sigma for 1-3 distances of 0.004
6. Others
 Sof(H6AA)=Sof(H6AB)=Sof(H2A)=Sof(H4A)=Sof(H3A)=Sof(H7AA)=Sof(H7AB)=Sof(H7AC)=
 1-FVAR(1)
 Sof(C6A)=Sof(C5A)=Sof(C2A)=Sof(C4A)=Sof(C3A)=Sof(S1A)=Sof(N7A)=0.5*(1-FVAR(2))
 Sof(N7)=Sof(C5)=Sof(C2)=Sof(C4)=Sof(C3)=Sof(S1)=Sof(C6)=0.5*FVAR(2)
 Sof(H7A)=Sof(H7B)=Sof(H7C)=Sof(H2)=Sof(H4)=Sof(H3)=Sof(H6A)=Sof(H6B)=FVAR(1)
7.a Secondary CH2 refined with riding coordinates:
 C6A(H6AA,H6AB), C6(H6A,H6B)
7.b Aromatic/amide H refined with riding coordinates:
 C2(H2), C4(H4), C3(H3), C2A(H2A), C4A(H4A), C3A(H3A)
7.c Idealised Me refined as rotating group:
 N7(H7A,H7B,H7C), N7A(H7AA,H7AB,H7AC)

This report has been created with Olex2, compiled on 2024.02.16 svn.r378c4104 for OlexSys. Please [let us know](mailto:support@olex2.org?subject=Olex2%20Report) if there are any errors or if you would like to have additional features.
