# Supplementary material for: Temperature-Dependent Structural and Optoelectronic Properties of the Layered Perovskite 2-Thiophenemethylammonium Lead Iodide
Source: J Phys Chem C Nanomater Interfaces. 2024 Jul 25;128(31):13108–20. doi: 10.1021/acs.jpcc.4c03221 (PMC11317984; doi:10.1021/acs.jpcc.4c03221)
Supplement: Supplementary file 1 — jp4c03221_si_001.zip [file jp4c03221_si_001.zip › ThMA2PbI4_Temp-depSCXRD/datareport_125k.docx]

**ThMA2PbI4_2_125**

| **Table 1 Crystal data and structure refinement for ThMA2PbI4_2_125.** | |
| --- | --- |
| Identification code | ThMA2PbI4_2_125 |
| Empirical formula | C_20_H_64_I_8_N_4_Pb_2_S_4_ |
| Formula weight | 1918.57 |
| Temperature/K | 125.02(10) |
| Crystal system | orthorhombic |
| Space group | Cmce |
| a/Å | 29.0528(8) |
| b/Å | 8.6802(2) |
| c/Å | 8.6921(2) |
| α/° | 90 |
| β/° | 90 |
| γ/° | 90 |
| Volume/Å^3^ | 2192.01(9) |
| Z | 2 |
| ρ_calc_g/cm^3^ | 2.907 |
| μ/mm^‑1^ | 13.514 |
| F(000) | 1728.0 |
| Crystal size/mm^3^ | 1.0 × 0.08 × 0.02 |
| Radiation | Mo Kα (λ = 0.71073) |
| 2Θ range for data collection/° | 5.608 to 58.248 |
| Index ranges | -39 ≤ h ≤ 39, -11 ≤ k ≤ 11, -11 ≤ l ≤ 11 |
| Reflections collected | 10487 |
| Independent reflections | 1502 [R_int_ = 0.0389, R_sigma_ = 0.0247] |
| Data/restraints/parameters | 1502/337/134 |
| Goodness-of-fit on F^2^ | 1.368 |
| Final R indexes [I>=2σ (I)] | R_1_ = 0.0370, wR_2_ = 0.0813 |
| Final R indexes [all data] | R_1_ = 0.0390, wR_2_ = 0.0818 |
| Largest diff. peak/hole / e Å^-3^ | 2.28/-2.39 |

| **Table 2 Fractional Atomic Coordinates (×10^4^) and Equivalent Isotropic Displacement Parameters (Å^2^×10^3^) for ThMA2PbI4_2_125. U_eq_ is defined as 1/3 of the trace of the orthogonalised U_IJ_ tensor.** | | | | |
| --- | --- | --- | --- | --- |
| **Atom** | ***x*** | ***y*** | ***z*** | **U(eq)** |
| Pb01 | 5000 | 5000 | -5000 | 17.32(15) |
| I002 | 5000 | 6831.8(8) | -1834.1(8) | 21.44(18) |
| I003 | 6099.9(3) | 5000 | -5000 | 33.1(2) |
| N7 | 5938(19) | 5760(60) | 800(60) | 27(4) |
| C5 | 6672(8) | 5010(30) | -200(40) | 41(5) |
| C2 | 7486(9) | 4330(40) | 10(70) | 33(5) |
| C6A | 6177(10) | 5160(60) | -390(40) | 36(4) |
| C4 | 6961(9) | 6250(40) | -380(60) | 40(7) |
| C3 | 7415(10) | 5880(40) | -170(60) | 33(5) |
| S1 | 6975(4) | 3370(13) | 210(20) | 48(4) |
| C5A | 6685(8) | 5120(40) | 80(30) | 41(5) |
| C2A | 7480(9) | 5230(60) | -670(40) | 32(7) |
| C4A | 6974(9) | 4990(60) | 1320(40) | 40(7) |
| C3A | 7427(10) | 5050(60) | 880(40) | 28(7) |
| S1A | 6967(4) | 5362(19) | -1636(13) | 45(4) |
| C6 | 6159(10) | 4610(40) | -210(60) | 36(4) |
| N7A | 5920(20) | 5910(70) | 870(60) | 27(4) |

| **Table 3 Anisotropic Displacement Parameters (Å^2^×10^3^) for ThMA2PbI4_2_125. The Anisotropic displacement factor exponent takes the form: -2π^2^[h^2^a*^2^U_11_+2hka*b*U_12_+…].** | | | | | | |
| --- | --- | --- | --- | --- | --- | --- |
| **Atom** | **U_11_** | **U_22_** | **U_33_** | **U_23_** | **U_13_** | **U_12_** |
| Pb01 | 20.5(3) | 16.0(3) | 15.5(3) | -0.1(2) | 0 | 0 |
| I002 | 28.5(4) | 18.4(3) | 17.5(3) | -5.3(3) | 0 | 0 |
| I003 | 20.4(4) | 40.2(5) | 38.6(5) | 0.4(4) | 0 | 0 |
| N7 | 21(7) | 30(9) | 29(7) | 5(6) | -2(6) | -3(6) |
| C5 | 22(6) | 57(10) | 42(11) | 3(9) | -3(9) | 2(8) |
| C2 | 19(6) | 43(10) | 35(11) | -1(9) | 6(9) | -1(8) |
| C6A | 24(5) | 47(9) | 36(9) | -2(8) | -1(6) | 1(7) |
| C4 | 22(8) | 55(12) | 44(15) | -3(13) | 4(11) | 4(8) |
| C3 | 19(6) | 43(10) | 35(11) | -1(9) | 6(9) | -1(8) |
| S1 | 30(5) | 33(6) | 80(9) | -3(6) | 2(5) | 2(4) |
| C5A | 23(5) | 57(11) | 43(10) | 3(9) | 1(8) | 2(9) |
| C2A | 20(10) | 48(19) | 28(11) | -5(12) | -2(9) | -7(13) |
| C4A | 22(9) | 56(15) | 41(11) | 1(12) | 3(8) | 0(11) |
| C3A | 21(8) | 36(17) | 28(11) | -4(11) | -1(9) | 5(12) |
| S1A | 27(5) | 81(10) | 28(6) | 6(5) | -1(4) | 1(5) |
| C6 | 24(5) | 47(9) | 36(9) | -2(8) | -1(6) | 1(7) |
| N7A | 21(7) | 30(9) | 29(7) | 5(6) | -2(6) | -3(6) |

| **Table 4 Bond Lengths for ThMA2PbI4_2_125.** | | | | | | |
| --- | --- | --- | --- | --- | --- | --- |
| **Atom** | **Atom** | **Length/Å** |  | **Atom** | **Atom** | **Length/Å** |
| Pb01 | I002 | 3.1782(7) |  | C2 | C3 | 1.369(17) |
| Pb01 | I002^1^ | 3.1782(7) |  | C2 | S1 | 1.71(2) |
| Pb01 | I002^2^ | 3.1787(7) |  | C6A | C5A | 1.531(15) |
| Pb01 | I002^3^ | 3.1787(7) |  | C6A | N7A | 1.482(13) |
| Pb01 | I003 | 3.1955(8) |  | C4 | C3 | 1.369(17) |
| Pb01 | I003^1^ | 3.1956(8) |  | C5A | C4A | 1.369(17) |
| N7 | C6 | 1.482(13) |  | C5A | S1A | 1.71(2) |
| C5 | C4 | 1.369(17) |  | C2A | C3A | 1.369(17) |
| C5 | S1 | 1.71(2) |  | C2A | S1A | 1.71(2) |
| C5 | C6 | 1.530(15) |  | C4A | C3A | 1.369(17) |

^1^1-X,1-Y,-1-Z; ^2^+X,-1/2+Y,-1/2-Z; ^3^1-X,3/2-Y,-1/2+Z

| **Table 5 Bond Angles for ThMA2PbI4_2_125.** | | | | | | | | |
| --- | --- | --- | --- | --- | --- | --- | --- | --- |
| **Atom** | **Atom** | **Atom** | **Angle/˚** |  | **Atom** | **Atom** | **Atom** | **Angle/˚** |
| I002 | Pb01 | I002^1^ | 180.0 |  | C4 | C5 | S1 | 111(2) |
| I002^1^ | Pb01 | I002^2^ | 90.081(7) |  | C4 | C5 | C6 | 141(3) |
| I002 | Pb01 | I002^2^ | 89.919(7) |  | C6 | C5 | S1 | 108(2) |
| I002^1^ | Pb01 | I002^3^ | 89.919(7) |  | C3 | C2 | S1 | 111(3) |
| I002 | Pb01 | I002^3^ | 90.081(7) |  | N7A | C6A | C5A | 108(3) |
| I002^2^ | Pb01 | I002^3^ | 180.000(13) |  | C3 | C4 | C5 | 113(3) |
| I002 | Pb01 | I003 | 90.0 |  | C4 | C3 | C2 | 113(4) |
| I002^2^ | Pb01 | I003 | 90.0 |  | C5 | S1 | C2 | 91.0(17) |
| I002^1^ | Pb01 | I003^1^ | 90.0 |  | C6A | C5A | S1A | 103(2) |
| I002^3^ | Pb01 | I003 | 90.0 |  | C4A | C5A | C6A | 143(3) |
| I002^3^ | Pb01 | I003^1^ | 90.0 |  | C4A | C5A | S1A | 113(2) |
| I002^1^ | Pb01 | I003 | 90.0 |  | C3A | C2A | S1A | 113(3) |
| I002^2^ | Pb01 | I003^1^ | 90.0 |  | C5A | C4A | C3A | 112(3) |
| I002 | Pb01 | I003^1^ | 90.0 |  | C2A | C3A | C4A | 113(4) |
| I003 | Pb01 | I003^1^ | 180.0 |  | C5A | S1A | C2A | 89.0(17) |
| Pb01 | I002 | Pb01^4^ | 150.12(3) |  | N7 | C6 | C5 | 105(3) |

^1^1-X,1-Y,-1-Z; ^2^+X,-1/2+Y,-1/2-Z; ^3^1-X,3/2-Y,-1/2+Z; ^4^1-X,3/2-Y,1/2+Z

| **Table 6 Torsion Angles for ThMA2PbI4_2_125.** | | | | | | | | | | |
| --- | --- | --- | --- | --- | --- | --- | --- | --- | --- | --- |
| **A** | **B** | **C** | **D** | **Angle/˚** |  | **A** | **B** | **C** | **D** | **Angle/˚** |
| C5 | C4 | C3 | C2 | 7(4) |  | C5A | C4A | C3A | C2A | 0(4) |
| C6A | C5A | C4A | C3A | 179.6(16) |  | C4A | C5A | S1A | C2A | 2(2) |
| C6A | C5A | S1A | C2A | -179.1(12) |  | C3A | C2A | S1A | C5A | -1(2) |
| C4 | C5 | S1 | C2 | -4(3) |  | S1A | C5A | C4A | C3A | -1(3) |
| C4 | C5 | C6 | N7 | -49(4) |  | S1A | C2A | C3A | C4A | 1(4) |
| C3 | C2 | S1 | C5 | 8(3) |  | C6 | C5 | C4 | C3 | 178(3) |
| S1 | C5 | C4 | C3 | -1(4) |  | C6 | C5 | S1 | C2 | 177.1(18) |
| S1 | C5 | C6 | N7 | 129(4) |  | N7A | C6A | C5A | C4A | 34(4) |
| S1 | C2 | C3 | C4 | -9(4) |  | N7A | C6A | C5A | S1A | -145(4) |

| **Table 7 Hydrogen Atom Coordinates (Å×10^4^) and Isotropic Displacement Parameters (Å^2^×10^3^) for ThMA2PbI4_2_125.** | | | | |
| --- | --- | --- | --- | --- |
| **Atom** | ***x*** | ***y*** | ***z*** | **U(eq)** |
| H7A | 6074.91 | 6696.05 | 663.53 | 32 |
| H7B | 5970.68 | 5467.39 | 1801.94 | 32 |
| H7C | 5633.94 | 5833.89 | 568.24 | 32 |
| H2 | 7780.24 | 3853.88 | 13.79 | 39 |
| H6AA | 6139.22 | 5740.76 | -1360.49 | 43 |
| H6AB | 6062.33 | 4096.02 | -552.92 | 43 |
| H4 | 6856.77 | 7255.03 | -621.06 | 49 |
| H3 | 7655.4 | 6623.04 | -154.67 | 39 |
| H2A | 7771.55 | 5278.67 | -1165.75 | 38 |
| H4A | 6873.43 | 4879.4 | 2348.51 | 47 |
| H3A | 7676.85 | 4968.66 | 1582.87 | 34 |
| H6A | 6033.54 | 4674.54 | -1268.99 | 43 |
| H6B | 6109.2 | 3553.44 | 184.83 | 43 |
| H7AA | 6103.94 | 6053.44 | 1691.19 | 32 |
| H7AB | 5674.22 | 5305.85 | 1141.19 | 32 |
| H7AC | 5809.63 | 6841.16 | 535.19 | 32 |

| **Table 8 Atomic Occupancy for ThMA2PbI4_2_125.** | | | | | | | |
| --- | --- | --- | --- | --- | --- | --- | --- |
| **Atom** | ***Occupancy*** |  | **Atom** | ***Occupancy*** |  | **Atom** | ***Occupancy*** |
| N7 | 0.255(5) |  | H7A | 0.510(11) |  | H7B | 0.510(11) |
| H7C | 0.510(11) |  | C5 | 0.255(5) |  | C2 | 0.255(5) |
| H2 | 0.510(11) |  | C6A | 0.245(5) |  | H6AA | 0.490(11) |
| H6AB | 0.490(11) |  | C4 | 0.255(5) |  | H4 | 0.510(11) |
| C3 | 0.255(5) |  | H3 | 0.510(11) |  | S1 | 0.255(5) |
| C5A | 0.245(5) |  | C2A | 0.245(5) |  | H2A | 0.490(11) |
| C4A | 0.245(5) |  | H4A | 0.490(11) |  | C3A | 0.245(5) |
| H3A | 0.490(11) |  | S1A | 0.245(5) |  | C6 | 0.255(5) |
| H6A | 0.510(11) |  | H6B | 0.510(11) |  | N7A | 0.245(5) |
| H7AA | 0.490(11) |  | H7AB | 0.490(11) |  | H7AC | 0.490(11) |

**Experimental**

Single crystals of C_20_H_64_I_8_N_4_Pb_2_S_4_ **[ThMA2PbI4_2_125]** were **[]**. A suitable crystal was selected and **[]** on a **XtaLAB Synergy, Dualflex, HyPix-Arc 100** diffractometer. The crystal was kept at 125.02(10) K during data collection. Using Olex2 [1], the structure was solved with the SHELXT [2] structure solution program using Intrinsic Phasing and refined with the SHELXL [3] refinement package using Least Squares minimisation.

1. Dolomanov, O.V., Bourhis, L.J., Gildea, R.J, Howard, J.A.K. & Puschmann, H. (2009), J. Appl. Cryst. 42, 339-341.
2. Sheldrick, G.M. (2015). Acta Cryst. A71, 3-8.
3. Sheldrick, G.M. (2015). Acta Cryst. C71, 3-8.

**Crystal structure determination of [ThMA2PbI4_2_125]**

**Crystal Data** for C_20_H_64_I_8_N_4_Pb_2_S_4_ (*M*=1918.57 g/mol): orthorhombic, space group Cmce (no. 64), *a* = 29.0528(8) Å, *b* = 8.6802(2) Å, *c* = 8.6921(2) Å, *V*= 2192.01(9) Å^3^, *Z* = 2, *T* = 125.02(10) K, μ(Mo Kα) = 13.514 mm^-1^, *Dcalc* = 2.907 g/cm^3^, 10487 reflections measured (5.608° ≤ 2Θ ≤ 58.248°), 1502 unique (*R*_int_ = 0.0389, R_sigma_ = 0.0247) which were used in all calculations. The final *R*_1_ was 0.0370 (I > 2σ(I)) and *wR*_2_ was 0.0818 (all data).

**Refinement model description**

Number of restraints - 337, number of constraints - unknown.

Details:

1. Fixed Uiso
 At 1.2 times of:
 All C(H) groups, All C(H,H) groups, All N(H,H,H) groups
2. Restrained distances
 C6A-C5A = C6-C5
 1.54 with sigma of 0.02
 N7-C6 = N7A-C6A
 1.48 with sigma of 0.02
 N7A-C6A ≈ N7-C6
 with sigma of 0.02
 C6A-C5A ≈ C6-C5
 with sigma of 0.02
 S1-C2 ≈ S1-C5 ≈ S1A-C2A ≈ S1A-C5A
 with sigma of 0.002
 C5A-C4A ≈ C4A-C3A ≈ C3A-C2A ≈ C3-C2 ≈ C4-C3 ≈ C5-C4
 with sigma of 0.002
 N7-C6 ≈ N7A-C6A
 with sigma of 0.002
3. Restrained planarity
 C6A, C5A, S1A, C2A, C3A, C4A
 with sigma of 0.1
 C6, C5, C4, C3, C2, S1
 with sigma of 0.1
4. Uiso/Uaniso restraints and constraints
All non-hydrogen atoms have similar U: within 2A with sigma of 0.04 and sigma
for terminal atoms of 0.08 within 2A
C6A ≈ C6 ≈ C5A ≈ C5: within 2A with sigma of 0.02 and sigma for
terminal atoms of 0.04 within 2A
N7 ≈ N7A ≈ C5A ≈ C5 ≈ C6A ≈ C6: within 2A with sigma of
0.02 and sigma for terminal atoms of 0.04 within 2A
C2 ≈ C3: within 2A with sigma of 0.002 and sigma for terminal atoms of
0.004 within 2A
Uanis(C6A) = Uanis(C6)
Uanis(N7A) = Uanis(N7)
5. Rigid body (RIGU) restrains
 All non-hydrogen atoms
 with sigma for 1-2 distances of 0.004 and sigma for 1-3 distances of 0.004
6. Others
 Sof(H6AA)=Sof(H6AB)=Sof(H2A)=Sof(H4A)=Sof(H3A)=Sof(H7AA)=Sof(H7AB)=Sof(H7AC)=
 1-FVAR(1)
 Sof(C6A)=Sof(C5A)=Sof(C2A)=Sof(C4A)=Sof(C3A)=Sof(S1A)=Sof(N7A)=0.5*(1-FVAR(2))
 Sof(N7)=Sof(C5)=Sof(C2)=Sof(C4)=Sof(C3)=Sof(S1)=Sof(C6)=0.5*FVAR(2)
 Sof(H7A)=Sof(H7B)=Sof(H7C)=Sof(H2)=Sof(H4)=Sof(H3)=Sof(H6A)=Sof(H6B)=FVAR(1)
7.a Secondary CH2 refined with riding coordinates:
 C6A(H6AA,H6AB), C6(H6A,H6B)
7.b Aromatic/amide H refined with riding coordinates:
 C2(H2), C4(H4), C3(H3), C2A(H2A), C4A(H4A), C3A(H3A)
7.c Idealised Me refined as rotating group:
 N7(H7A,H7B,H7C), N7A(H7AA,H7AB,H7AC)

This report has been created with Olex2, compiled on 2024.02.16 svn.r378c4104 for OlexSys. Please [let us know](mailto:support@olex2.org?subject=Olex2%20Report) if there are any errors or if you would like to have additional features.
