# Supplementary material for: Temperature-Dependent Structural and Optoelectronic Properties of the Layered Perovskite 2-Thiophenemethylammonium Lead Iodide
Source: J Phys Chem C Nanomater Interfaces. 2024 Jul 25;128(31):13108–20. doi: 10.1021/acs.jpcc.4c03221 (PMC11317984; doi:10.1021/acs.jpcc.4c03221)
Supplement: Supplementary file 1 — jp4c03221_si_001.zip [file jp4c03221_si_001.zip › ThMA2PbI4_Temp-depSCXRD/datareport_150k.docx]

**ThMA2PbI4_3_150**

| **Table 1 Crystal data and structure refinement for ThMA2PbI4_3_150.** | |
| --- | --- |
| Identification code | ThMA2PbI4_3_150 |
| Empirical formula | C_24_H_48_I_8_N_4_Pb_2_S_8_ |
| Formula weight | 2078.72 |
| Temperature/K | 150.00(10) |
| Crystal system | orthorhombic |
| Space group | Cmce |
| a/Å | 29.0629(8) |
| b/Å | 8.6884(2) |
| c/Å | 8.6993(2) |
| α/° | 90 |
| β/° | 90 |
| γ/° | 90 |
| Volume/Å^3^ | 2196.66(9) |
| Z | 2 |
| ρ_calc_g/cm^3^ | 3.143 |
| μ/mm^‑1^ | 13.683 |
| F(000) | 1872.0 |
| Crystal size/mm^3^ | 1 × 0.08 × 0.02 |
| Radiation | Mo Kα (λ = 0.71073) |
| 2Θ range for data collection/° | 5.606 to 54.948 |
| Index ranges | -37 ≤ h ≤ 37, -11 ≤ k ≤ 11, -11 ≤ l ≤ 11 |
| Reflections collected | 9209 |
| Independent reflections | 1280 [R_int_ = 0.0391, R_sigma_ = 0.0233] |
| Data/restraints/parameters | 1280/2/72 |
| Goodness-of-fit on F^2^ | 1.307 |
| Final R indexes [I>=2σ (I)] | R_1_ = 0.0440, wR_2_ = 0.1108 |
| Final R indexes [all data] | R_1_ = 0.0458, wR_2_ = 0.1117 |
| Largest diff. peak/hole / e Å^-3^ | 1.99/-1.79 |

| **Table 2 Fractional Atomic Coordinates (×10^4^) and Equivalent Isotropic Displacement Parameters (Å^2^×10^3^) for ThMA2PbI4_3_150. U_eq_ is defined as 1/3 of the trace of the orthogonalised U_IJ_ tensor.** | | | | |
| --- | --- | --- | --- | --- |
| **Atom** | ***x*** | ***y*** | ***z*** | **U(eq)** |
| Pb01 | 5000 | 5000 | 0 | 20.1(2) |
| I002 | 5000 | 3164.1(10) | 3161.8(10) | 25.4(3) |
| I003 | 3900.9(4) | 5000 | 0 | 37.9(3) |
| S004 | 3030(4) | 3420(20) | 4783(17) | 135(3) |
| C005 | 3034(4) | 4762(18) | 3400(20) | 135(3) |
| C2 | 3325(5) | 5000 | 5000 | 57(6) |
| C3 | 2550(10) | 4250(40) | 4900(50) | 41(6) |
| C6 | 2545(11) | 4950(50) | 4240(40) | 46(8) |
| C004 | 3030(4) | 3420(20) | 4783(17) | 135(3) |
| S005 | 3034(4) | 4762(18) | 3400(20) | 135(3) |
| N1 | 4070(6) | 4171(18) | 5822(19) | 29(4) |
| C1 | 3844(4) | 5230(30) | 4720(30) | 47(8) |

| **Table 3 Anisotropic Displacement Parameters (Å^2^×10^3^) for ThMA2PbI4_3_150. The Anisotropic displacement factor exponent takes the form: -2π^2^[h^2^a*^2^U_11_+2hka*b*U_12_+…].** | | | | | | |
| --- | --- | --- | --- | --- | --- | --- |
| **Atom** | **U_11_** | **U_22_** | **U_33_** | **U_23_** | **U_13_** | **U_12_** |
| Pb01 | 24.0(4) | 17.6(4) | 18.7(4) | 0.0(3) | 0 | 0 |
| I002 | 33.9(5) | 20.6(4) | 21.6(4) | 6.3(4) | 0 | 0 |
| I003 | 24.5(5) | 44.5(6) | 44.7(7) | -0.6(5) | 0 | 0 |
| S004 | 81(5) | 171(10) | 153(9) | 13(6) | 0(5) | 3(6) |
| C005 | 81(5) | 171(10) | 153(9) | 13(6) | 0(5) | 3(6) |
| C2 | 22(8) | 77(16) | 70(15) | -12(11) | 0 | 0 |
| C3 | 34(13) | 50(18) | 39(15) | -8(17) | -2(12) | 0(13) |
| C6 | 45(15) | 46(17) | 50(20) | 20(20) | -7(14) | -5(13) |
| C004 | 81(5) | 171(10) | 153(9) | 13(6) | 0(5) | 3(6) |
| S005 | 81(5) | 171(10) | 153(9) | 13(6) | 0(5) | 3(6) |
| N1 | 24(9) | 27(9) | 38(10) | 7(8) | -1(8) | -2(7) |
| C1 | 28(11) | 60(20) | 50(20) | 44(14) | -6(11) | -3(12) |

| **Table 4 Bond Lengths for ThMA2PbI4_3_150.** | | | | | | |
| --- | --- | --- | --- | --- | --- | --- |
| **Atom** | **Atom** | **Length/Å** |  | **Atom** | **Atom** | **Length/Å** |
| Pb01 | I002^1^ | 3.1796(9) |  | S004 | C3 | 1.58(3) |
| Pb01 | I002^2^ | 3.1803(9) |  | C005 | C2 | 1.641(17) |
| Pb01 | I002^3^ | 3.1803(9) |  | C005 | C6 | 1.61(4) |
| Pb01 | I002 | 3.1796(9) |  | C2 | C1 | 1.5399(10) |
| Pb01 | I003 | 3.1944(10) |  | C3 | C3^4^ | 1.31(6) |
| Pb01 | I003^1^ | 3.1944(10) |  | C6 | C6^4^ | 1.32(6) |
| S004 | C2 | 1.630(18) |  | N1 | C1 | 1.4801(10) |

^1^1-X,1-Y,-Z; ^2^+X,1/2+Y,1/2-Z; ^3^1-X,1/2-Y,-1/2+Z; ^4^+X,1-Y,1-Z

| **Table 5 Bond Angles for ThMA2PbI4_3_150.** | | | | | | | | |
| --- | --- | --- | --- | --- | --- | --- | --- | --- |
| **Atom** | **Atom** | **Atom** | **Angle/˚** |  | **Atom** | **Atom** | **Atom** | **Angle/˚** |
| I002^1^ | Pb01 | I002 | 180.0 |  | I003^1^ | Pb01 | I003 | 180.0 |
| I002 | Pb01 | I002^2^ | 89.924(8) |  | Pb01 | I002 | Pb01^4^ | 150.29(3) |
| I002^1^ | Pb01 | I002^2^ | 90.075(8) |  | C3 | S004 | C2 | 94.0(15) |
| I002 | Pb01 | I002^3^ | 90.076(8) |  | C6 | C005 | C2 | 93.3(15) |
| I002^1^ | Pb01 | I002^3^ | 89.925(8) |  | S004 | C2 | S004^5^ | 116.6(12) |
| I002^2^ | Pb01 | I002^3^ | 180.0 |  | S004^5^ | C2 | C005^5^ | 61.6(7) |
| I002^1^ | Pb01 | I003^1^ | 90.0 |  | S004 | C2 | C005^5^ | 86.2(8) |
| I002 | Pb01 | I003 | 90.0 |  | C1 | C2 | S004^5^ | 115.0(12) |
| I002 | Pb01 | I003^1^ | 90.0 |  | C1 | C2 | S004 | 127.3(13) |
| I002^1^ | Pb01 | I003 | 90.0 |  | C1 | C2 | C005^5^ | 128.4(13) |
| I002^2^ | Pb01 | I003^1^ | 90.0 |  | C3^5^ | C3 | S004 | 117.7(13) |
| I002^3^ | Pb01 | I003^1^ | 90.0 |  | C6^5^ | C6 | C005 | 117.5(13) |
| I002^3^ | Pb01 | I003 | 90.0 |  | N1 | C1 | C2 | 104.6(11) |
| I002^2^ | Pb01 | I003 | 90.0 |  |  |  |  |  |

^1^1-X,1-Y,-Z; ^2^+X,1/2+Y,1/2-Z; ^3^1-X,1/2-Y,-1/2+Z; ^4^1-X,1/2-Y,1/2+Z; ^5^+X,1-Y,1-Z

| **Table 6 Torsion Angles for ThMA2PbI4_3_150.** | | | | | | | | | | |
| --- | --- | --- | --- | --- | --- | --- | --- | --- | --- | --- |
| **A** | **B** | **C** | **D** | **Angle/˚** |  | **A** | **B** | **C** | **D** | **Angle/˚** |
| S004 | C2 | C1 | N1 | -61(2) |  | C3 | S004 | C2 | S004^1^ | -0.1(15) |
| S004^1^ | C2 | C1 | N1 | 131.0(17) |  | C3 | S004 | C2 | C1 | -167.6(18) |
| C2 | S004 | C3 | C3^1^ | 0(5) |  | C6 | C005 | C2 | C005^1^ | 1.9(15) |
| C2 | C005 | C6 | C6^1^ | -6(5) |  |  |  |  |  |  |

^1^+X,1-Y,1-Z

| **Table 7 Hydrogen Atom Coordinates (Å×10^4^) and Isotropic Displacement Parameters (Å^2^×10^3^) for ThMA2PbI4_3_150.** | | | | |
| --- | --- | --- | --- | --- |
| **Atom** | ***x*** | ***y*** | ***z*** | **U(eq)** |
| H00C | 3093.43 | 5577.37 | 2632.47 | 162 |
| H00D | 3077.89 | 3731.54 | 2938.52 | 162 |
| H2B | 3150.47 | 5583.45 | 4232.24 | 85 |
| H2A | 3244.88 | 5365.65 | 6031.51 | 85 |
| H2C | 3249.53 | 3904.23 | 4911.89 | 85 |
| H00A | 3081.05 | 2924.94 | 3771.87 | 162 |
| H00B | 3081.94 | 2668.87 | 5622.64 | 162 |
| H1A | 4379.4 | 4190.64 | 5670.36 | 35 |
| H1B | 3962.97 | 3198.2 | 5671.2 | 35 |
| H1C | 4005.46 | 4473.49 | 6800.61 | 35 |
| H1D | 3926.94 | 4966.18 | 3652.39 | 56 |
| H1E | 3934.39 | 6309.8 | 4927.92 | 56 |

| **Table 8 Atomic Occupancy for ThMA2PbI4_3_150.** | | | | | | | |
| --- | --- | --- | --- | --- | --- | --- | --- |
| **Atom** | ***Occupancy*** |  | **Atom** | ***Occupancy*** |  | **Atom** | ***Occupancy*** |
| S004 | 0.5 |  | C005 | 0.5 |  | H00C | 0.5 |
| H00D | 0.5 |  | H2B | 0.5 |  | H2A | 0.5 |
| H2C | 0.5 |  | C3 | 0.5 |  | C6 | 0.5 |
| C004 | 0.5 |  | H00A | 0.5 |  | H00B | 0.5 |
| S005 | 0.5 |  | N1 | 0.5 |  | H1A | 0.5 |
| H1B | 0.5 |  | H1C | 0.5 |  | C1 | 0.5 |
| H1D | 0.5 |  | H1E | 0.5 |  |  |  |

**Experimental**

Single crystals of C_24_H_48_I_8_N_4_Pb_2_S_8_ **[ThMA2PbI4_3_150]** were **[]**. A suitable crystal was selected and **[]** on a **XtaLAB Synergy, Dualflex, HyPix-Arc 100** diffractometer. The crystal was kept at 150.00(10) K during data collection. Using Olex2 [1], the structure was solved with the SHELXT [2] structure solution program using Intrinsic Phasing and refined with the SHELXL [3] refinement package using Least Squares minimisation.

1. Dolomanov, O.V., Bourhis, L.J., Gildea, R.J, Howard, J.A.K. & Puschmann, H. (2009), J. Appl. Cryst. 42, 339-341.
2. Sheldrick, G.M. (2015). Acta Cryst. A71, 3-8.
3. Sheldrick, G.M. (2015). Acta Cryst. C71, 3-8.

**Crystal structure determination of [ThMA2PbI4_3_150]**

**Crystal Data** for C_24_H_48_I_8_N_4_Pb_2_S_8_ (*M*=2078.72 g/mol): orthorhombic, space group Cmce (no. 64), *a* = 29.0629(8) Å, *b* = 8.6884(2) Å, *c* = 8.6993(2) Å, *V*= 2196.66(9) Å^3^, *Z* = 2, *T* = 150.00(10) K, μ(Mo Kα) = 13.683 mm^-1^, *Dcalc* = 3.143 g/cm^3^, 9209 reflections measured (5.606° ≤ 2Θ ≤ 54.948°), 1280 unique (*R*_int_ = 0.0391, R_sigma_ = 0.0233) which were used in all calculations. The final *R*_1_ was 0.0440 (I > 2σ(I)) and *wR*_2_ was 0.1117 (all data).

**Refinement model description**

Number of restraints - 2, number of constraints - unknown.

Details:

1. Fixed Uiso
 At 1.2 times of:
 All C(H,H) groups, All N(H,H,H) groups
 At 1.5 times of:
 All C(H,H,H) groups
2. Shared sites
{S004, C004}
{C005, S005}
3. Restrained distances
 N1-C1
 1.48 with sigma of 0.001
 C2-C1
 1.54 with sigma of 0.001
4. Uiso/Uaniso restraints and constraints
Uanis(S004) = Uanis(C004)
Uanis(C005) = Uanis(S005)
Uanis(S004) = Uanis(C004) = Uanis(C005) = Uanis(S005)
5. Others
 Fixed Sof: S004(0.5) C005(0.5) H00C(0.5) H00D(0.5) H2B(0.5) H2A(0.5) H2C(0.5)
 C3(0.5) C6(0.5) C004(0.5) H00A(0.5) H00B(0.5) S005(0.5) N1(0.5) H1A(0.5)
 H1B(0.5) H1C(0.5) C1(0.5) H1D(0.5) H1E(0.5)
6.a Secondary CH2 refined with riding coordinates:
 C005(H00C,H00D), C004(H00A,H00B), C1(H1D,H1E)
6.b Idealised Me refined as rotating group:
 C2(H2B,H2A,H2C), N1(H1A,H1B,H1C)

This report has been created with Olex2, compiled on 2024.02.16 svn.r378c4104 for OlexSys. Please [let us know](mailto:support@olex2.org?subject=Olex2%20Report) if there are any errors or if you would like to have additional features.
