# Supplementary material for: Temperature-Dependent Structural and Optoelectronic Properties of the Layered Perovskite 2-Thiophenemethylammonium Lead Iodide
Source: J Phys Chem C Nanomater Interfaces. 2024 Jul 25;128(31):13108–20. doi: 10.1021/acs.jpcc.4c03221 (PMC11317984; doi:10.1021/acs.jpcc.4c03221)
Supplement: Supplementary file 1 — jp4c03221_si_001.zip [file jp4c03221_si_001.zip › ThMA2PbI4_Temp-depSCXRD/datareport_200k.docx]

**ThMA2PbI4_5_200**

| **Table 1 Crystal data and structure refinement for ThMA2PbI4_5_200.** | |
| --- | --- |
| Identification code | ThMA2PbI4_5_200 |
| Empirical formula | C_20_H_64_I_8_N_4_Pb_2_S_4_ |
| Formula weight | 1918.57 |
| Temperature/K | 200.00(10) |
| Crystal system | orthorhombic |
| Space group | Cmce |
| a/Å | 29.1176(9) |
| b/Å | 8.7065(2) |
| c/Å | 8.7173(2) |
| α/° | 90 |
| β/° | 90 |
| γ/° | 90 |
| Volume/Å^3^ | 2209.94(10) |
| Z | 2 |
| ρ_calc_g/cm^3^ | 2.883 |
| μ/mm^‑1^ | 13.405 |
| F(000) | 1728.0 |
| Crystal size/mm^3^ | 1.0 × 0.08 × 0.02 |
| Radiation | Mo Kα (λ = 0.71073) |
| 2Θ range for data collection/° | 5.596 to 54.968 |
| Index ranges | -37 ≤ h ≤ 37, -11 ≤ k ≤ 11, -11 ≤ l ≤ 11 |
| Reflections collected | 9235 |
| Independent reflections | 1291 [R_int_ = 0.0400, R_sigma_ = 0.0235] |
| Data/restraints/parameters | 1291/324/133 |
| Goodness-of-fit on F^2^ | 1.265 |
| Final R indexes [I>=2σ (I)] | R_1_ = 0.0370, wR_2_ = 0.0798 |
| Final R indexes [all data] | R_1_ = 0.0400, wR_2_ = 0.0807 |
| Largest diff. peak/hole / e Å^-3^ | 1.71/-1.69 |

| **Table 2 Fractional Atomic Coordinates (×10^4^) and Equivalent Isotropic Displacement Parameters (Å^2^×10^3^) for ThMA2PbI4_5_200. U_eq_ is defined as 1/3 of the trace of the orthogonalised U_IJ_ tensor.** | | | | |
| --- | --- | --- | --- | --- |
| **Atom** | ***x*** | ***y*** | ***z*** | **U(eq)** |
| Pb01 | 5000 | 5000 | -5000 | 25.97(17) |
| I002 | 5000 | 6843.5(9) | -1846.0(9) | 33.2(2) |
| I003 | 6098.2(3) | 5000 | -5000 | 47.3(3) |
| N7 | 5900(20) | 5810(80) | 700(70) | 42(6) |
| C5 | 6662(9) | 5010(30) | -210(50) | 55(6) |
| C2 | 7486(9) | 4350(40) | -10(70) | 48(8) |
| C6A | 6187(11) | 5080(80) | -390(50) | 51(5) |
| C4 | 6938(10) | 6230(50) | -440(70) | 60(8) |
| C3 | 7383(11) | 5840(40) | -260(70) | 57(8) |
| S1 | 6975(4) | 3382(16) | 190(30) | 68(5) |
| C5A | 6691(9) | 5090(50) | 100(30) | 56(5) |
| C2A | 7475(9) | 5260(60) | -630(40) | 51(9) |
| C4A | 6961(10) | 5000(70) | 1360(50) | 60(8) |
| C3A | 7402(11) | 5080(60) | 890(40) | 52(8) |
| S1A | 6966(5) | 5350(20) | -1623(18) | 71(5) |
| C6 | 6151(11) | 4610(50) | -170(80) | 51(5) |
| N7A | 5940(20) | 5800(90) | 910(70) | 42(6) |

| **Table 3 Anisotropic Displacement Parameters (Å^2^×10^3^) for ThMA2PbI4_5_200. The Anisotropic displacement factor exponent takes the form: -2π^2^[h^2^a*^2^U_11_+2hka*b*U_12_+…].** | | | | | | |
| --- | --- | --- | --- | --- | --- | --- |
| **Atom** | **U_11_** | **U_22_** | **U_33_** | **U_23_** | **U_13_** | **U_12_** |
| Pb01 | 31.7(3) | 21.8(3) | 24.5(3) | 0.0(2) | 0 | 0 |
| I002 | 44.4(5) | 26.1(4) | 29.0(4) | -8.0(3) | 0 | 0 |
| I003 | 31.4(5) | 54.1(6) | 56.6(6) | 0.0(5) | 0 | 0 |
| N7 | 31(11) | 49(9) | 46(13) | 4(10) | -7(11) | -8(7) |
| C5 | 35(7) | 70(10) | 60(12) | 3(10) | -7(10) | 2(9) |
| C2 | 22(10) | 65(14) | 60(20) | 4(14) | -1(15) | -9(11) |
| C6A | 38(6) | 61(10) | 53(10) | 0(9) | -5(7) | -1(8) |
| C4 | 39(10) | 71(13) | 69(17) | -1(14) | 0(13) | 1(10) |
| C3 | 38(10) | 68(14) | 66(19) | 9(14) | 3(14) | -3(11) |
| S1 | 41(6) | 50(7) | 112(12) | 0(7) | 5(7) | 0(5) |
| C5A | 36(7) | 70(11) | 61(11) | 5(10) | -2(9) | 1(10) |
| C2A | 29(11) | 70(20) | 57(15) | 1(14) | -10(11) | -9(15) |
| C4A | 39(11) | 78(19) | 64(13) | 5(15) | -5(10) | -4(14) |
| C3A | 39(10) | 60(20) | 55(14) | -3(14) | -9(11) | -6(14) |
| S1A | 49(7) | 108(13) | 57(8) | 9(8) | 0(6) | 1(7) |
| C6 | 38(6) | 61(10) | 53(10) | 0(9) | -5(7) | -1(8) |
| N7A | 31(11) | 49(9) | 46(13) | 4(10) | -7(11) | -8(7) |

| **Table 4 Bond Lengths for ThMA2PbI4_5_200.** | | | | | | |
| --- | --- | --- | --- | --- | --- | --- |
| **Atom** | **Atom** | **Length/Å** |  | **Atom** | **Atom** | **Length/Å** |
| Pb01 | I002^1^ | 3.1836(8) |  | C2 | C3 | 1.349(19) |
| Pb01 | I002 | 3.1836(8) |  | C2 | S1 | 1.72(2) |
| Pb01 | I002^2^ | 3.1847(8) |  | C6A | C5A | 1.530(15) |
| Pb01 | I002^3^ | 3.1847(8) |  | C6A | N7A | 1.474(16) |
| Pb01 | I003^1^ | 3.1976(9) |  | C4 | C3 | 1.349(19) |
| Pb01 | I003 | 3.1977(9) |  | C5A | C4A | 1.349(19) |
| N7 | C6 | 1.474(16) |  | C5A | S1A | 1.72(2) |
| C5 | C4 | 1.349(19) |  | C2A | C3A | 1.349(19) |
| C5 | S1 | 1.72(2) |  | C2A | S1A | 1.72(2) |
| C5 | C6 | 1.529(15) |  | C4A | C3A | 1.349(19) |

^1^1-X,1-Y,-1-Z; ^2^+X,-1/2+Y,-1/2-Z; ^3^1-X,3/2-Y,-1/2+Z

| **Table 5 Bond Angles for ThMA2PbI4_5_200.** | | | | | | | | |
| --- | --- | --- | --- | --- | --- | --- | --- | --- |
| **Atom** | **Atom** | **Atom** | **Angle/˚** |  | **Atom** | **Atom** | **Atom** | **Angle/˚** |
| I002^1^ | Pb01 | I002 | 180.0 |  | C4 | C5 | S1 | 111(2) |
| I002 | Pb01 | I002^2^ | 89.924(7) |  | C4 | C5 | C6 | 140(3) |
| I002^1^ | Pb01 | I002^2^ | 90.076(7) |  | C6 | C5 | S1 | 109(3) |
| I002 | Pb01 | I002^3^ | 90.076(7) |  | C3 | C2 | S1 | 107(3) |
| I002^1^ | Pb01 | I002^3^ | 89.924(7) |  | N7A | C6A | C5A | 104(4) |
| I002^2^ | Pb01 | I002^3^ | 180.000(14) |  | C5 | C4 | C3 | 111(4) |
| I002^1^ | Pb01 | I003^1^ | 90.0 |  | C2 | C3 | C4 | 119(4) |
| I002^2^ | Pb01 | I003^1^ | 90.0 |  | C2 | S1 | C5 | 92.1(18) |
| I002 | Pb01 | I003 | 90.0 |  | C6A | C5A | S1A | 102(3) |
| I002^3^ | Pb01 | I003^1^ | 90.0 |  | C4A | C5A | C6A | 142(3) |
| I002^3^ | Pb01 | I003 | 90.0 |  | C4A | C5A | S1A | 117(2) |
| I002 | Pb01 | I003^1^ | 90.0 |  | C3A | C2A | S1A | 111(3) |
| I002^2^ | Pb01 | I003 | 90.0 |  | C3A | C4A | C5A | 108(4) |
| I002^1^ | Pb01 | I003 | 90.0 |  | C4A | C3A | C2A | 117(4) |
| I003^1^ | Pb01 | I003 | 180.0 |  | C5A | S1A | C2A | 87.3(18) |
| Pb01 | I002 | Pb01^4^ | 150.63(3) |  | N7 | C6 | C5 | 109(4) |

^1^1-X,1-Y,-1-Z; ^2^+X,-1/2+Y,-1/2-Z; ^3^1-X,3/2-Y,-1/2+Z; ^4^1-X,3/2-Y,1/2+Z

| **Table 6 Torsion Angles for ThMA2PbI4_5_200.** | | | | | | | | | | |
| --- | --- | --- | --- | --- | --- | --- | --- | --- | --- | --- |
| **A** | **B** | **C** | **D** | **Angle/˚** |  | **A** | **B** | **C** | **D** | **Angle/˚** |
| C5 | C4 | C3 | C2 | 5(5) |  | C5A | C4A | C3A | C2A | 2(4) |
| C6A | C5A | C4A | C3A | 179.4(17) |  | C4A | C5A | S1A | C2A | 2(2) |
| C6A | C5A | S1A | C2A | -178.9(12) |  | C3A | C2A | S1A | C5A | -1(2) |
| C4 | C5 | S1 | C2 | -1(3) |  | S1A | C5A | C4A | C3A | -3(3) |
| C4 | C5 | C6 | N7 | -45(5) |  | S1A | C2A | C3A | C4A | 0(4) |
| C3 | C2 | S1 | C5 | 4(3) |  | C6 | C5 | C4 | C3 | 178(3) |
| S1 | C5 | C4 | C3 | -2(4) |  | C6 | C5 | S1 | C2 | 178.9(19) |
| S1 | C5 | C6 | N7 | 135(5) |  | N7A | C6A | C5A | C4A | 32(5) |
| S1 | C2 | C3 | C4 | -6(5) |  | N7A | C6A | C5A | S1A | -146(5) |

| **Table 7 Hydrogen Atom Coordinates (Å×10^4^) and Isotropic Displacement Parameters (Å^2^×10^3^) for ThMA2PbI4_5_200.** | | | | |
| --- | --- | --- | --- | --- |
| **Atom** | ***x*** | ***y*** | ***z*** | **U(eq)** |
| H7A | 6111.52 | 6420.08 | 1180.12 | 51 |
| H7B | 5717.89 | 5356.68 | 1401.91 | 51 |
| H7C | 5732.89 | 6379.11 | 37.86 | 51 |
| H2 | 7785.43 | 3915.2 | 44.47 | 58 |
| H6AA | 6142.16 | 5681.85 | -1338.66 | 61 |
| H6AB | 6076.99 | 4019.62 | -555.11 | 61 |
| H4 | 6833.33 | 7230.73 | -702.63 | 71 |
| H3 | 7617.69 | 6592.66 | -307.45 | 69 |
| H2A | 7770.06 | 5330.1 | -1090.31 | 62 |
| H4A | 6859.47 | 4898.82 | 2389.44 | 73 |
| H3A | 7649.89 | 5013.08 | 1593.02 | 62 |
| H6A | 6029.09 | 4546.43 | -1230.52 | 61 |
| H6B | 6105.71 | 3594.8 | 325.86 | 61 |
| H7AA | 6034.91 | 5350.73 | 1804.84 | 51 |
| H7AB | 5636.11 | 5678.44 | 792.71 | 51 |
| H7AC | 6012.27 | 6821.96 | 943.99 | 51 |

| **Table 8 Atomic Occupancy for ThMA2PbI4_5_200.** | | | | | | | |
| --- | --- | --- | --- | --- | --- | --- | --- |
| **Atom** | ***Occupancy*** |  | **Atom** | ***Occupancy*** |  | **Atom** | ***Occupancy*** |
| N7 | 0.252(6) |  | H7A | 0.504(12) |  | H7B | 0.504(12) |
| H7C | 0.504(12) |  | C5 | 0.252(6) |  | C2 | 0.252(6) |
| H2 | 0.504(12) |  | C6A | 0.248(6) |  | H6AA | 0.496(12) |
| H6AB | 0.496(12) |  | C4 | 0.252(6) |  | H4 | 0.504(12) |
| C3 | 0.252(6) |  | H3 | 0.504(12) |  | S1 | 0.252(6) |
| C5A | 0.248(6) |  | C2A | 0.248(6) |  | H2A | 0.496(12) |
| C4A | 0.248(6) |  | H4A | 0.496(12) |  | C3A | 0.248(6) |
| H3A | 0.496(12) |  | S1A | 0.248(6) |  | C6 | 0.252(6) |
| H6A | 0.504(12) |  | H6B | 0.504(12) |  | N7A | 0.248(6) |
| H7AA | 0.496(12) |  | H7AB | 0.496(12) |  | H7AC | 0.496(12) |

**Experimental**

Single crystals of C_20_H_64_I_8_N_4_Pb_2_S_4_ **[ThMA2PbI4_5_200]** were **[]**. A suitable crystal was selected and **[]** on a **XtaLAB Synergy, Dualflex, HyPix-Arc 100** diffractometer. The crystal was kept at 200.00(10) K during data collection. Using Olex2 [1], the structure was solved with the SHELXT [2] structure solution program using Intrinsic Phasing and refined with the SHELXL [3] refinement package using Least Squares minimisation.

1. Dolomanov, O.V., Bourhis, L.J., Gildea, R.J, Howard, J.A.K. & Puschmann, H. (2009), J. Appl. Cryst. 42, 339-341.
2. Sheldrick, G.M. (2015). Acta Cryst. A71, 3-8.
3. Sheldrick, G.M. (2015). Acta Cryst. C71, 3-8.

**Crystal structure determination of [ThMA2PbI4_5_200]**

**Crystal Data** for C_20_H_64_I_8_N_4_Pb_2_S_4_ (*M*=1918.57 g/mol): orthorhombic, space group Cmce (no. 64), *a* = 29.1176(9) Å, *b* = 8.7065(2) Å, *c* = 8.7173(2) Å, *V*= 2209.94(10) Å^3^, *Z* = 2, *T* = 200.00(10) K, μ(Mo Kα) = 13.405 mm^-1^, *Dcalc* = 2.883 g/cm^3^, 9235 reflections measured (5.596° ≤ 2Θ ≤ 54.968°), 1291 unique (*R*_int_ = 0.0400, R_sigma_ = 0.0235) which were used in all calculations. The final *R*_1_ was 0.0370 (I > 2σ(I)) and *wR*_2_ was 0.0807 (all data).

**Refinement model description**

Number of restraints - 324, number of constraints - unknown.

Details:

1. Fixed Uiso
 At 1.2 times of:
 All C(H) groups, All C(H,H) groups, All N(H,H,H) groups
2. Restrained distances
 C6A-C5A = C6-C5
 1.54 with sigma of 0.02
 N7-C6 = N7A-C6A
 1.48 with sigma of 0.02
 N7A-C6A ≈ N7-C6
 with sigma of 0.02
 C6A-C5A ≈ C6-C5
 with sigma of 0.02
 S1-C2 ≈ S1-C5 ≈ S1A-C2A ≈ S1A-C5A
 with sigma of 0.002
 C5A-C4A ≈ C4A-C3A ≈ C3A-C2A ≈ C3-C2 ≈ C4-C3 ≈ C5-C4
 with sigma of 0.002
3. Restrained planarity
 C6A, C5A, S1A, C2A, C3A, C4A
 with sigma of 0.1
 C6, C5, C4, C3, C2, S1
 with sigma of 0.1
4. Uiso/Uaniso restraints and constraints
All non-hydrogen atoms have similar U: within 2A with sigma of 0.04 and sigma
for terminal atoms of 0.08 within 2A
C6A ≈ C6 ≈ C5A ≈ C5: within 2A with sigma of 0.02 and sigma for
terminal atoms of 0.04 within 2A
N7 ≈ N7A ≈ C5A ≈ C5 ≈ C6A ≈ C6: within 2A with sigma of
0.02 and sigma for terminal atoms of 0.04 within 2A
Uanis(C6A) = Uanis(C6)
Uanis(N7A) = Uanis(N7)
5. Rigid body (RIGU) restrains
 All non-hydrogen atoms
 with sigma for 1-2 distances of 0.004 and sigma for 1-3 distances of 0.004
6. Others
 Sof(H6AA)=Sof(H6AB)=Sof(H2A)=Sof(H4A)=Sof(H3A)=Sof(H7AA)=Sof(H7AB)=Sof(H7AC)=
 1-FVAR(1)
 Sof(C6A)=Sof(C5A)=Sof(C2A)=Sof(C4A)=Sof(C3A)=Sof(S1A)=Sof(N7A)=0.5*(1-FVAR(2))
 Sof(N7)=Sof(C5)=Sof(C2)=Sof(C4)=Sof(C3)=Sof(S1)=Sof(C6)=0.5*FVAR(2)
 Sof(H7A)=Sof(H7B)=Sof(H7C)=Sof(H2)=Sof(H4)=Sof(H3)=Sof(H6A)=Sof(H6B)=FVAR(1)
7.a Secondary CH2 refined with riding coordinates:
 C6A(H6AA,H6AB), C6(H6A,H6B)
7.b Aromatic/amide H refined with riding coordinates:
 C2(H2), C4(H4), C3(H3), C2A(H2A), C4A(H4A), C3A(H3A)
7.c Idealised Me refined as rotating group:
 N7(H7A,H7B,H7C), N7A(H7AA,H7AB,H7AC)

This report has been created with Olex2, compiled on 2024.02.16 svn.r378c4104 for OlexSys. Please [let us know](mailto:support@olex2.org?subject=Olex2%20Report) if there are any errors or if you would like to have additional features.
