# Supplementary material for: Temperature-Dependent Structural and Optoelectronic Properties of the Layered Perovskite 2-Thiophenemethylammonium Lead Iodide
Source: J Phys Chem C Nanomater Interfaces. 2024 Jul 25;128(31):13108–20. doi: 10.1021/acs.jpcc.4c03221 (PMC11317984; doi:10.1021/acs.jpcc.4c03221)
Supplement: Supplementary file 1 — jp4c03221_si_001.zip [file jp4c03221_si_001.zip › ThMA2PbI4_Temp-depSCXRD/datareport_225k.docx]

**ThMA2PbI4_6_225**

| **Table 1 Crystal data and structure refinement for ThMA2PbI4_6_225.** | |
| --- | --- |
| Identification code | ThMA2PbI4_6_225 |
| Empirical formula | C_20_H_64_I_8_N_4_Pb_2_S_4_ |
| Formula weight | 1918.57 |
| Temperature/K | 225.00(10) |
| Crystal system | orthorhombic |
| Space group | Cmce |
| a/Å | 29.1372(10) |
| b/Å | 8.7145(2) |
| c/Å | 8.7249(3) |
| α/° | 90 |
| β/° | 90 |
| γ/° | 90 |
| Volume/Å^3^ | 2215.39(12) |
| Z | 2 |
| ρ_calc_g/cm^3^ | 2.876 |
| μ/mm^‑1^ | 13.372 |
| F(000) | 1728.0 |
| Crystal size/mm^3^ | 1.0 × 0.08 × 0.02 |
| Radiation | Mo Kα (λ = 0.71073) |
| 2Θ range for data collection/° | 5.592 to 54.942 |
| Index ranges | -37 ≤ h ≤ 37, -11 ≤ k ≤ 11, -11 ≤ l ≤ 11 |
| Reflections collected | 9299 |
| Independent reflections | 1293 [R_int_ = 0.0398, R_sigma_ = 0.0231] |
| Data/restraints/parameters | 1293/324/133 |
| Goodness-of-fit on F^2^ | 1.218 |
| Final R indexes [I>=2σ (I)] | R_1_ = 0.0369, wR_2_ = 0.0819 |
| Final R indexes [all data] | R_1_ = 0.0402, wR_2_ = 0.0830 |
| Largest diff. peak/hole / e Å^-3^ | 1.24/-1.47 |

| **Table 2 Fractional Atomic Coordinates (×10^4^) and Equivalent Isotropic Displacement Parameters (Å^2^×10^3^) for ThMA2PbI4_6_225. U_eq_ is defined as 1/3 of the trace of the orthogonalised U_IJ_ tensor.** | | | | |
| --- | --- | --- | --- | --- |
| **Atom** | ***x*** | ***y*** | ***z*** | **U(eq)** |
| Pb01 | 5000 | 5000 | -5000 | 29.44(17) |
| I002 | 5000 | 6848.4(9) | -1851.6(9) | 38.1(2) |
| I003 | 6097.2(3) | 5000 | -5000 | 52.9(3) |
| N7 | 5920(30) | 5820(90) | 690(70) | 45(5) |
| C5 | 6680(9) | 5070(30) | -280(40) | 57(6) |
| C2 | 7479(9) | 4350(40) | -70(60) | 56(9) |
| C6A | 6165(11) | 5160(70) | -440(50) | 53(5) |
| C4 | 6953(10) | 6280(50) | -580(70) | 65(8) |
| C3 | 7389(12) | 5830(40) | -360(60) | 67(8) |
| S1 | 6967(5) | 3410(18) | 140(20) | 75(5) |
| C5A | 6670(9) | 5150(40) | 60(30) | 56(6) |
| C2A | 7466(9) | 5280(60) | -600(40) | 60(9) |
| C4A | 6959(10) | 5060(60) | 1250(50) | 63(8) |
| C3A | 7408(11) | 5100(60) | 910(40) | 63(9) |
| S1A | 6965(5) | 5370(20) | -1627(17) | 76(5) |
| C6 | 6172(11) | 4640(50) | -200(70) | 53(5) |
| N7A | 5920(30) | 5800(100) | 900(70) | 45(5) |

| **Table 3 Anisotropic Displacement Parameters (Å^2^×10^3^) for ThMA2PbI4_6_225. The Anisotropic displacement factor exponent takes the form: -2π^2^[h^2^a*^2^U_11_+2hka*b*U_12_+…].** | | | | | | |
| --- | --- | --- | --- | --- | --- | --- |
| **Atom** | **U_11_** | **U_22_** | **U_33_** | **U_23_** | **U_13_** | **U_12_** |
| Pb01 | 35.7(3) | 26.2(3) | 26.4(3) | -0.1(2) | 0 | 0 |
| I002 | 50.6(5) | 31.4(4) | 32.2(4) | -10.0(3) | 0 | 0 |
| I003 | 34.8(5) | 61.6(6) | 62.2(6) | -0.7(5) | 0 | 0 |
| N7 | 36(7) | 54(9) | 47(13) | 12(9) | -1(10) | -1(7) |
| C5 | 40(6) | 76(11) | 55(13) | 10(10) | 1(10) | 9(9) |
| C2 | 24(10) | 80(15) | 60(20) | 10(15) | 1(15) | -6(12) |
| C6A | 43(6) | 66(10) | 51(11) | 8(9) | 0(7) | 6(8) |
| C4 | 45(10) | 82(14) | 67(18) | 7(14) | 2(13) | 3(10) |
| C3 | 45(10) | 84(15) | 70(20) | 14(15) | 2(14) | 0(11) |
| S1 | 46(6) | 70(8) | 108(13) | 1(8) | 6(7) | -3(6) |
| C5A | 40(6) | 73(12) | 54(12) | 12(10) | 3(9) | 8(10) |
| C2A | 32(11) | 90(20) | 64(16) | 10(15) | -6(11) | -5(15) |
| C4A | 44(10) | 83(19) | 62(14) | 15(15) | -2(9) | 4(14) |
| C3A | 43(10) | 80(20) | 63(16) | 5(16) | -6(11) | 2(15) |
| S1A | 55(7) | 120(13) | 53(8) | 13(7) | -2(6) | 1(7) |
| C6 | 43(6) | 66(10) | 51(11) | 8(9) | 0(7) | 6(8) |
| N7A | 36(7) | 54(9) | 47(13) | 12(9) | -1(10) | -1(7) |

| **Table 4 Bond Lengths for ThMA2PbI4_6_225.** | | | | | | |
| --- | --- | --- | --- | --- | --- | --- |
| **Atom** | **Atom** | **Length/Å** |  | **Atom** | **Atom** | **Length/Å** |
| Pb01 | I002 | 3.1844(8) |  | C2 | C3 | 1.342(19) |
| Pb01 | I002^1^ | 3.1844(8) |  | C2 | S1 | 1.71(2) |
| Pb01 | I002^2^ | 3.1864(8) |  | C6A | C5A | 1.533(16) |
| Pb01 | I002^3^ | 3.1864(8) |  | C6A | N7A | 1.477(16) |
| Pb01 | I003^1^ | 3.1968(9) |  | C4 | C3 | 1.342(19) |
| Pb01 | I003 | 3.1968(9) |  | C5A | C4A | 1.342(19) |
| N7 | C6 | 1.476(16) |  | C5A | S1A | 1.71(2) |
| C5 | C4 | 1.342(19) |  | C2A | C3A | 1.342(19) |
| C5 | S1 | 1.71(2) |  | C2A | S1A | 1.71(2) |
| C5 | C6 | 1.530(16) |  | C4A | C3A | 1.342(19) |

^1^1-X,1-Y,-1-Z; ^2^+X,-1/2+Y,-1/2-Z; ^3^1-X,3/2-Y,-1/2+Z

| **Table 5 Bond Angles for ThMA2PbI4_6_225.** | | | | | | | | |
| --- | --- | --- | --- | --- | --- | --- | --- | --- |
| **Atom** | **Atom** | **Atom** | **Angle/˚** |  | **Atom** | **Atom** | **Atom** | **Angle/˚** |
| I002 | Pb01 | I002^1^ | 180.0 |  | C4 | C5 | S1 | 114(2) |
| I002^1^ | Pb01 | I002^2^ | 90.078(7) |  | C4 | C5 | C6 | 141(3) |
| I002 | Pb01 | I002^2^ | 89.923(7) |  | C6 | C5 | S1 | 105(3) |
| I002^1^ | Pb01 | I002^3^ | 89.923(7) |  | C3 | C2 | S1 | 108(3) |
| I002 | Pb01 | I002^3^ | 90.077(7) |  | N7A | C6A | C5A | 104(4) |
| I002^2^ | Pb01 | I002^3^ | 180.000(14) |  | C5 | C4 | C3 | 108(4) |
| I002 | Pb01 | I003^1^ | 90.0 |  | C4 | C3 | C2 | 119(4) |
| I002^2^ | Pb01 | I003^1^ | 90.0 |  | C5 | S1 | C2 | 89.8(18) |
| I002^1^ | Pb01 | I003 | 90.0 |  | C6A | C5A | S1A | 104(3) |
| I002^3^ | Pb01 | I003^1^ | 90.0 |  | C4A | C5A | C6A | 145(3) |
| I002^3^ | Pb01 | I003 | 90.0 |  | C4A | C5A | S1A | 111(2) |
| I002^1^ | Pb01 | I003^1^ | 90.0 |  | C3A | C2A | S1A | 114(3) |
| I002^2^ | Pb01 | I003 | 90.0 |  | C5A | C4A | C3A | 116(4) |
| I002 | Pb01 | I003 | 90.0 |  | C2A | C3A | C4A | 110(4) |
| I003^1^ | Pb01 | I003 | 180.0 |  | C2A | S1A | C5A | 88.6(18) |
| Pb01 | I002 | Pb01^4^ | 150.85(3) |  | N7 | C6 | C5 | 109(4) |

^1^1-X,1-Y,-1-Z; ^2^+X,-1/2+Y,-1/2-Z; ^3^1-X,3/2-Y,-1/2+Z; ^4^1-X,3/2-Y,1/2+Z

| **Table 6 Torsion Angles for ThMA2PbI4_6_225.** | | | | | | | | | | |
| --- | --- | --- | --- | --- | --- | --- | --- | --- | --- | --- |
| **A** | **B** | **C** | **D** | **Angle/˚** |  | **A** | **B** | **C** | **D** | **Angle/˚** |
| C5 | C4 | C3 | C2 | 7(5) |  | C5A | C4A | C3A | C2A | 2(4) |
| C6A | C5A | C4A | C3A | 179.3(18) |  | C4A | C5A | S1A | C2A | 1(2) |
| C6A | C5A | S1A | C2A | -179.5(12) |  | C3A | C2A | S1A | C5A | 0(3) |
| C4 | C5 | S1 | C2 | 1(3) |  | S1A | C5A | C4A | C3A | -2(4) |
| C4 | C5 | C6 | N7 | -47(5) |  | S1A | C2A | C3A | C4A | -1(4) |
| C3 | C2 | S1 | C5 | 3(3) |  | C6 | C5 | C4 | C3 | 177(3) |
| S1 | C5 | C4 | C3 | -4(4) |  | C6 | C5 | S1 | C2 | 179.9(19) |
| S1 | C5 | C6 | N7 | 134(5) |  | N7A | C6A | C5A | C4A | 28(6) |
| S1 | C2 | C3 | C4 | -7(5) |  | N7A | C6A | C5A | S1A | -150(5) |

| **Table 7 Hydrogen Atom Coordinates (Å×10^4^) and Isotropic Displacement Parameters (Å^2^×10^3^) for ThMA2PbI4_6_225.** | | | | |
| --- | --- | --- | --- | --- |
| **Atom** | ***x*** | ***y*** | ***z*** | **U(eq)** |
| H7A | 6027.57 | 5823.4 | 1668.72 | 54 |
| H7B | 5617.44 | 5600.18 | 681.16 | 54 |
| H7C | 5970.62 | 6753.59 | 253.96 | 54 |
| H2 | 7775.64 | 3903.48 | 9.43 | 67 |
| H6AA | 6120.5 | 5806.53 | -1354.6 | 64 |
| H6AB | 6057.14 | 4103.2 | -667.31 | 64 |
| H4 | 6855.52 | 7265.83 | -900.14 | 78 |
| H3 | 7631.24 | 6562.18 | -400.45 | 80 |
| H2A | 7760.18 | 5350.62 | -1066.97 | 72 |
| H4A | 6851.88 | 4966.75 | 2277.27 | 76 |
| H3A | 7649.06 | 5021.5 | 1639.86 | 76 |
| H6A | 6042.47 | 4565.43 | -1246.71 | 64 |
| H6B | 6137.49 | 3624.09 | 302.09 | 64 |
| H7AA | 5938.36 | 5128.16 | 1697.1 | 54 |
| H7AB | 5620.53 | 5953.9 | 651.11 | 54 |
| H7AC | 6051.03 | 6705.64 | 1170.98 | 54 |

| **Table 8 Atomic Occupancy for ThMA2PbI4_6_225.** | | | | | | | |
| --- | --- | --- | --- | --- | --- | --- | --- |
| **Atom** | ***Occupancy*** |  | **Atom** | ***Occupancy*** |  | **Atom** | ***Occupancy*** |
| N7 | 0.254(6) |  | H7A | 0.508(13) |  | H7B | 0.508(13) |
| H7C | 0.508(13) |  | C5 | 0.254(6) |  | C2 | 0.254(6) |
| H2 | 0.508(13) |  | C6A | 0.246(6) |  | H6AA | 0.492(13) |
| H6AB | 0.492(13) |  | C4 | 0.254(6) |  | H4 | 0.508(13) |
| C3 | 0.254(6) |  | H3 | 0.508(13) |  | S1 | 0.254(6) |
| C5A | 0.246(6) |  | C2A | 0.246(6) |  | H2A | 0.492(13) |
| C4A | 0.246(6) |  | H4A | 0.492(13) |  | C3A | 0.246(6) |
| H3A | 0.492(13) |  | S1A | 0.246(6) |  | C6 | 0.254(6) |
| H6A | 0.508(13) |  | H6B | 0.508(13) |  | N7A | 0.246(6) |
| H7AA | 0.492(13) |  | H7AB | 0.492(13) |  | H7AC | 0.492(13) |

**Experimental**

Single crystals of C_20_H_64_I_8_N_4_Pb_2_S_4_ **[ThMA2PbI4_6_225]** were **[]**. A suitable crystal was selected and **[]** on a **XtaLAB Synergy, Dualflex, HyPix-Arc 100** diffractometer. The crystal was kept at 225.00(10) K during data collection. Using Olex2 [1], the structure was solved with the SHELXT [2] structure solution program using Intrinsic Phasing and refined with the SHELXL [3] refinement package using Least Squares minimisation.

1. Dolomanov, O.V., Bourhis, L.J., Gildea, R.J, Howard, J.A.K. & Puschmann, H. (2009), J. Appl. Cryst. 42, 339-341.
2. Sheldrick, G.M. (2015). Acta Cryst. A71, 3-8.
3. Sheldrick, G.M. (2015). Acta Cryst. C71, 3-8.

**Crystal structure determination of [ThMA2PbI4_6_225]**

**Crystal Data** for C_20_H_64_I_8_N_4_Pb_2_S_4_ (*M*=1918.57 g/mol): orthorhombic, space group Cmce (no. 64), *a* = 29.1372(10) Å, *b* = 8.7145(2) Å, *c* = 8.7249(3) Å, *V*= 2215.39(12) Å^3^, *Z* = 2, *T* = 225.00(10) K, μ(Mo Kα) = 13.372 mm^-1^, *Dcalc* = 2.876 g/cm^3^, 9299 reflections measured (5.592° ≤ 2Θ ≤ 54.942°), 1293 unique (*R*_int_ = 0.0398, R_sigma_ = 0.0231) which were used in all calculations. The final *R*_1_ was 0.0369 (I > 2σ(I)) and *wR*_2_ was 0.0830 (all data).

**Refinement model description**

Number of restraints - 324, number of constraints - unknown.

Details:

1. Fixed Uiso
 At 1.2 times of:
 All C(H) groups, All C(H,H) groups, All N(H,H,H) groups
2. Restrained distances
 C6A-C5A = C6-C5
 1.54 with sigma of 0.02
 N7-C6 = N7A-C6A
 1.48 with sigma of 0.02
 N7A-C6A ≈ N7-C6
 with sigma of 0.02
 C6A-C5A ≈ C6-C5
 with sigma of 0.02
 S1-C2 ≈ S1-C5 ≈ S1A-C2A ≈ S1A-C5A
 with sigma of 0.002
 C5A-C4A ≈ C4A-C3A ≈ C3A-C2A ≈ C3-C2 ≈ C4-C3 ≈ C5-C4
 with sigma of 0.002
3. Restrained planarity
 C6A, C5A, S1A, C2A, C3A, C4A
 with sigma of 0.1
 C6, C5, C4, C3, C2, S1
 with sigma of 0.1
4. Uiso/Uaniso restraints and constraints
All non-hydrogen atoms have similar U: within 2A with sigma of 0.04 and sigma
for terminal atoms of 0.08 within 2A
C6A ≈ C6 ≈ C5A ≈ C5: within 2A with sigma of 0.02 and sigma for
terminal atoms of 0.04 within 2A
N7 ≈ N7A ≈ C5A ≈ C5 ≈ C6A ≈ C6: within 2A with sigma of
0.02 and sigma for terminal atoms of 0.04 within 2A
Uanis(C6A) = Uanis(C6)
Uanis(N7A) = Uanis(N7)
5. Rigid body (RIGU) restrains
 All non-hydrogen atoms
 with sigma for 1-2 distances of 0.004 and sigma for 1-3 distances of 0.004
6. Others
 Sof(H6AA)=Sof(H6AB)=Sof(H2A)=Sof(H4A)=Sof(H3A)=Sof(H7AA)=Sof(H7AB)=Sof(H7AC)=
 1-FVAR(1)
 Sof(C6A)=Sof(C5A)=Sof(C2A)=Sof(C4A)=Sof(C3A)=Sof(S1A)=Sof(N7A)=0.5*(1-FVAR(2))
 Sof(N7)=Sof(C5)=Sof(C2)=Sof(C4)=Sof(C3)=Sof(S1)=Sof(C6)=0.5*FVAR(2)
 Sof(H7A)=Sof(H7B)=Sof(H7C)=Sof(H2)=Sof(H4)=Sof(H3)=Sof(H6A)=Sof(H6B)=FVAR(1)
7.a Secondary CH2 refined with riding coordinates:
 C6A(H6AA,H6AB), C6(H6A,H6B)
7.b Aromatic/amide H refined with riding coordinates:
 C2(H2), C4(H4), C3(H3), C2A(H2A), C4A(H4A), C3A(H3A)
7.c Idealised Me refined as rotating group:
 N7(H7A,H7B,H7C), N7A(H7AA,H7AB,H7AC)

This report has been created with Olex2, compiled on 2024.02.16 svn.r378c4104 for OlexSys. Please [let us know](mailto:support@olex2.org?subject=Olex2%20Report) if there are any errors or if you would like to have additional features.
