# Supplementary material for: Temperature-Dependent Structural and Optoelectronic Properties of the Layered Perovskite 2-Thiophenemethylammonium Lead Iodide
Source: J Phys Chem C Nanomater Interfaces. 2024 Jul 25;128(31):13108–20. doi: 10.1021/acs.jpcc.4c03221 (PMC11317984; doi:10.1021/acs.jpcc.4c03221)
Supplement: Supplementary file 1 — jp4c03221_si_001.zip [file jp4c03221_si_001.zip › ThMA2PbI4_Temp-depSCXRD/datareport_275k.docx]

**ThMA2PbI4_8_275**

| **Table 1 Crystal data and structure refinement for ThMA2PbI4_8_275.** | |
| --- | --- |
| Identification code | ThMA2PbI4_8_275 |
| Empirical formula | C_20_H_32_I_8_N_4_Pb_2_S_4_ |
| Formula weight | 1886.31 |
| Temperature/K | 275.00(10) |
| Crystal system | orthorhombic |
| Space group | Pbca |
| a/Å | 8.8341(3) |
| b/Å | 8.6898(4) |
| c/Å | 29.0358(13) |
| α/° | 90 |
| β/° | 90 |
| γ/° | 90 |
| Volume/Å^3^ | 2228.98(16) |
| Z | 2 |
| ρ_calc_g/cm^3^ | 2.811 |
| μ/mm^‑1^ | 13.288 |
| F(000) | 1664.0 |
| Crystal size/mm^3^ | 1 × 0.08 × 0.02 |
| Radiation | Mo Kα (λ = 0.71073) |
| 2Θ range for data collection/° | 5.398 to 54.97 |
| Index ranges | -11 ≤ h ≤ 11, -11 ≤ k ≤ 11, -37 ≤ l ≤ 37 |
| Reflections collected | 18515 |
| Independent reflections | 2553 [R_int_ = 0.0537, R_sigma_ = 0.0348] |
| Data/restraints/parameters | 2553/103/135 |
| Goodness-of-fit on F^2^ | 1.028 |
| Final R indexes [I>=2σ (I)] | R_1_ = 0.0384, wR_2_ = 0.0817 |
| Final R indexes [all data] | R_1_ = 0.0580, wR_2_ = 0.0892 |
| Largest diff. peak/hole / e Å^-3^ | 0.95/-0.68 |

| **Table 2 Fractional Atomic Coordinates (×10^4^) and Equivalent Isotropic Displacement Parameters (Å^2^×10^3^) for ThMA2PbI4_8_275. U_eq_ is defined as 1/3 of the trace of the orthogonalised U_IJ_ tensor.** | | | | |
| --- | --- | --- | --- | --- |
| **Atom** | ***x*** | ***y*** | ***z*** | **U(eq)** |
| Pb1 | 10000 | 5000 | 5000 | 38.15(7) |
| I1 | 8081.7(4) | 1908.5(5) | 5026.4(2) | 50.87(11) |
| I2 | 9673.7(5) | 5156.9(5) | 3897.7(2) | 55.97(12) |
| N1 | 5701(7) | 4300(7) | 4076.5(19) | 66.2(15) |
| C2 | 4527(9) | 5343(9) | 3892(3) | 78.4(19) |
| C3 | 4350(11) | 5159(11) | 3377(3) | 76(3) |
| S4 | 5429(7) | 6163(7) | 3020.7(14) | 157(2) |
| C7 | 3234(11) | 4166(14) | 3148(4) | 93(3) |
| C6 | 3451(16) | 4383(17) | 2676(4) | 117(3) |
| C5 | 4514(15) | 5420(16) | 2549(4) | 113(3) |
| C3A | 4448(12) | 5277(13) | 3368(4) | 65(4) |
| S4A | 3500(14) | 3912(12) | 3088(3) | 177(5) |
| C7A | 5178(16) | 6418(15) | 3065(4) | 50(3) |
| C6A | 4848(18) | 5959(18) | 2618(5) | 101(4) |
| C5A | 3968(19) | 4711(17) | 2564(5) | 94(4) |

| **Table 3 Anisotropic Displacement Parameters (Å^2^×10^3^) for ThMA2PbI4_8_275. The Anisotropic displacement factor exponent takes the form: -2π^2^[h^2^a*^2^U_11_+2hka*b*U_12_+…].** | | | | | | |
| --- | --- | --- | --- | --- | --- | --- |
| **Atom** | **U_11_** | **U_22_** | **U_33_** | **U_23_** | **U_13_** | **U_12_** |
| Pb1 | 32.79(13) | 31.66(14) | 50.00(17) | -0.70(14) | 1.23(12) | -0.23(11) |
| I1 | 43.35(19) | 40.79(19) | 68.5(3) | 3.38(19) | -7.71(18) | -14.38(17) |
| I2 | 62.0(2) | 59.1(3) | 46.9(2) | -4.2(2) | 5.71(19) | -1.5(2) |
| N1 | 62(3) | 69(3) | 67(3) | 7(3) | 5(2) | -13(3) |
| C2 | 88(4) | 74(4) | 73(2) | 16(3) | 6(3) | 3(3) |
| C3 | 67(5) | 86(6) | 75(3) | 1(4) | 4(3) | 21(4) |
| S4 | 211(5) | 191(5) | 68(2) | 14(3) | 15(3) | -65(5) |
| C7 | 48(5) | 127(7) | 103(3) | -25(4) | 6(3) | 23(4) |
| C6 | 111(7) | 143(7) | 97(3) | -31(5) | -7(4) | 8(4) |
| C5 | 128(7) | 144(7) | 68(4) | -23(5) | -8(5) | 3(4) |
| C3A | 81(9) | 40(6) | 73(3) | -3(3) | -6(4) | -6(5) |
| S4A | 243(11) | 163(9) | 124(7) | 5(6) | -28(7) | -77(9) |
| C7A | 54(7) | 35(5) | 60(3) | 5(3) | -11(4) | 20(4) |
| C6A | 134(9) | 104(5) | 66(3) | -5(5) | -11(5) | -48(4) |
| C5A | 114(9) | 96(5) | 70(6) | 0(6) | -18(7) | -35(4) |

| **Table 4 Bond Lengths for ThMA2PbI4_8_275.** | | | | | | |
| --- | --- | --- | --- | --- | --- | --- |
| **Atom** | **Atom** | **Length/Å** |  | **Atom** | **Atom** | **Length/Å** |
| Pb1 | I1 | 3.1772(4) |  | C3 | C7 | 1.469(14) |
| Pb1 | I1^1^ | 3.1772(4) |  | S4 | C5 | 1.717(12) |
| Pb1 | I1^2^ | 3.1887(4) |  | C7 | C6 | 1.397(15) |
| Pb1 | I1^3^ | 3.1887(4) |  | C6 | C5 | 1.352(17) |
| Pb1 | I2 | 3.2163(5) |  | C3A | S4A | 1.665(14) |
| Pb1 | I2^1^ | 3.2163(5) |  | C3A | C7A | 1.476(16) |
| N1 | C2 | 1.478(10) |  | S4A | C5A | 1.721(15) |
| C2 | C3 | 1.512(11) |  | C7A | C6A | 1.388(17) |
| C2 | C3A | 1.522(13) |  | C6A | C5A | 1.343(18) |
| C3 | S4 | 1.655(11) |  |  |  |  |

^1^2-X,1-Y,1-Z; ^2^1/2+X,1/2-Y,1-Z; ^3^3/2-X,1/2+Y,+Z

| **Table 5 Bond Angles for ThMA2PbI4_8_275.** | | | | | | | | |
| --- | --- | --- | --- | --- | --- | --- | --- | --- |
| **Atom** | **Atom** | **Atom** | **Angle/˚** |  | **Atom** | **Atom** | **Atom** | **Angle/˚** |
| I1 | Pb1 | I1^1^ | 180.0 |  | N1 | C2 | C3 | 111.5(6) |
| I1^1^ | Pb1 | I1^2^ | 90.928(5) |  | N1 | C2 | C3A | 111.8(6) |
| I1 | Pb1 | I1^2^ | 89.072(5) |  | C2 | C3 | S4 | 120.1(7) |
| I1^1^ | Pb1 | I1^3^ | 89.072(5) |  | C7 | C3 | C2 | 125.4(9) |
| I1 | Pb1 | I1^3^ | 90.928(5) |  | C7 | C3 | S4 | 114.4(7) |
| I1^2^ | Pb1 | I1^3^ | 180.0 |  | C3 | S4 | C5 | 91.6(6) |
| I1 | Pb1 | I2^1^ | 89.308(11) |  | C6 | C7 | C3 | 105.8(10) |
| I1^2^ | Pb1 | I2 | 85.722(11) |  | C5 | C6 | C7 | 116.9(12) |
| I1^1^ | Pb1 | I2^1^ | 90.691(11) |  | C6 | C5 | S4 | 111.2(9) |
| I1 | Pb1 | I2 | 90.693(11) |  | C2 | C3A | S4A | 122.6(9) |
| I1^2^ | Pb1 | I2^1^ | 94.278(11) |  | C7A | C3A | C2 | 123.4(10) |
| I1^3^ | Pb1 | I2^1^ | 85.722(11) |  | C7A | C3A | S4A | 114.0(8) |
| I1^1^ | Pb1 | I2 | 89.309(11) |  | C3A | S4A | C5A | 91.4(8) |
| I1^3^ | Pb1 | I2 | 94.278(11) |  | C6A | C7A | C3A | 105.8(11) |
| I2^1^ | Pb1 | I2 | 180.0 |  | C5A | C6A | C7A | 117.6(14) |
| Pb1 | I1 | Pb1^4^ | 153.451(15) |  | C6A | C5A | S4A | 111.2(11) |

^1^2-X,1-Y,1-Z; ^2^3/2-X,1/2+Y,+Z; ^3^1/2+X,1/2-Y,1-Z; ^4^-1/2+X,1/2-Y,1-Z

| **Table 6 Torsion Angles for ThMA2PbI4_8_275.** | | | | | | | | | | |
| --- | --- | --- | --- | --- | --- | --- | --- | --- | --- | --- |
| **A** | **B** | **C** | **D** | **Angle/˚** |  | **A** | **B** | **C** | **D** | **Angle/˚** |
| N1 | C2 | C3 | S4 | -87.3(8) |  | C3 | C7 | C6 | C5 | -2.3(15) |
| N1 | C2 | C3 | C7 | 94.7(9) |  | S4 | C3 | C7 | C6 | 0.4(10) |
| N1 | C2 | C3A | S4A | 82.2(10) |  | C7 | C3 | S4 | C5 | 1.1(7) |
| N1 | C2 | C3A | C7A | -98.7(10) |  | C7 | C6 | C5 | S4 | 3.2(17) |
| C2 | C3 | S4 | C5 | -177.1(8) |  | C3A | S4A | C5A | C6A | 1.3(11) |
| C2 | C3 | C7 | C6 | 178.5(10) |  | C3A | C7A | C6A | C5A | 2.0(16) |
| C2 | C3A | S4A | C5A | 179.0(9) |  | S4A | C3A | C7A | C6A | -0.9(13) |
| C2 | C3A | C7A | C6A | 179.9(9) |  | C7A | C3A | S4A | C5A | -0.2(10) |
| C3 | S4 | C5 | C6 | -2.4(12) |  | C7A | C6A | C5A | S4A | -2.2(17) |

| **Table 7 Hydrogen Atom Coordinates (Å×10^4^) and Isotropic Displacement Parameters (Å^2^×10^3^) for ThMA2PbI4_8_275.** | | | | |
| --- | --- | --- | --- | --- |
| **Atom** | ***x*** | ***y*** | ***z*** | **U(eq)** |
| H1A | 5535.55 | 3349.11 | 3974.21 | 79 |
| H1B | 5664.01 | 4306.13 | 4382.85 | 79 |
| H1C | 6608.98 | 4616.15 | 3983.76 | 79 |
| H2AA | 4799.32 | 6399.47 | 3961.09 | 94 |
| H2AB | 3567.93 | 5125.76 | 4041.11 | 94 |
| H2BC | 4747.61 | 6389.1 | 3987.28 | 94 |
| H2BD | 3550.44 | 5059.89 | 4019.09 | 94 |
| H7 | 2528.12 | 3525.39 | 3288.29 | 111 |
| H6 | 2890.23 | 3840.51 | 2458.49 | 140 |
| H5 | 4721.85 | 5694.63 | 2245.99 | 136 |
| H7A | 5744.06 | 7269.97 | 3154.42 | 60 |
| H6A | 5221.62 | 6495.79 | 2365.59 | 122 |
| H5A | 3652.93 | 4328.88 | 2280.79 | 112 |

| **Table 8 Atomic Occupancy for ThMA2PbI4_8_275.** | | | | | | | |
| --- | --- | --- | --- | --- | --- | --- | --- |
| **Atom** | ***Occupancy*** |  | **Atom** | ***Occupancy*** |  | **Atom** | ***Occupancy*** |
| H2AA | 0.651(6) |  | H2AB | 0.651(6) |  | H2BC | 0.349(6) |
| H2BD | 0.349(6) |  | C3 | 0.651(6) |  | S4 | 0.651(6) |
| C7 | 0.651(6) |  | H7 | 0.651(6) |  | C6 | 0.651(6) |
| H6 | 0.651(6) |  | C5 | 0.651(6) |  | H5 | 0.651(6) |
| C3A | 0.349(6) |  | S4A | 0.349(6) |  | C7A | 0.349(6) |
| H7A | 0.349(6) |  | C6A | 0.349(6) |  | H6A | 0.349(6) |
| C5A | 0.349(6) |  | H5A | 0.349(6) |  |  |  |

**Experimental**

Single crystals of C_20_H_32_I_8_N_4_Pb_2_S_4_ **[ThMA2PbI4_8_275]** were **[]**. A suitable crystal was selected and **[]** on a **XtaLAB Synergy, Dualflex, HyPix-Arc 100** diffractometer. The crystal was kept at 275.00(10) K during data collection. Using Olex2 [1], the structure was solved with the SHELXT [2] structure solution program using Intrinsic Phasing and refined with the SHELXL [3] refinement package using Least Squares minimisation.

1. Dolomanov, O.V., Bourhis, L.J., Gildea, R.J, Howard, J.A.K. & Puschmann, H. (2009), J. Appl. Cryst. 42, 339-341.
2. Sheldrick, G.M. (2015). Acta Cryst. A71, 3-8.
3. Sheldrick, G.M. (2015). Acta Cryst. C71, 3-8.

**Crystal structure determination of [ThMA2PbI4_8_275]**

**Crystal Data** for C_20_H_32_I_8_N_4_Pb_2_S_4_ (*M*=1886.31 g/mol): orthorhombic, space group Pbca (no. 61), *a* = 8.8341(3) Å, *b* = 8.6898(4) Å, *c* = 29.0358(13) Å, *V*= 2228.98(16) Å^3^, *Z* = 2, *T* = 275.00(10) K, μ(Mo Kα) = 13.288 mm^-1^, *Dcalc* = 2.811 g/cm^3^, 18515 reflections measured (5.398° ≤ 2Θ ≤ 54.97°), 2553 unique (*R*_int_ = 0.0537, R_sigma_ = 0.0348) which were used in all calculations. The final *R*_1_ was 0.0384 (I > 2σ(I)) and *wR*_2_ was 0.0892 (all data).

**Refinement model description**

Number of restraints - 103, number of constraints - unknown.

Details:

1. Fixed Uiso
 At 1.2 times of:
 All C(H) groups, All C(H,H,H,H) groups, All N(H,H,H) groups
2. Restrained distances
 C2-C3 ≈ C2-C3A
 with sigma of 0.02
 C3-S4 ≈ C3A-S4A
 with sigma of 0.02
 C3-C7 ≈ C3A-C7A
 with sigma of 0.02
 S4-C5 ≈ S4A-C5A
 with sigma of 0.02
 C7-C6 ≈ C7A-C6A
 with sigma of 0.02
 C6-C5 ≈ C6A-C5A
 with sigma of 0.02
 C2-S4 ≈ C2-S4A
 with sigma of 0.04
 C2-C7 ≈ C2-C7A
 with sigma of 0.04
 C3-C6 ≈ C3A-C6A
 with sigma of 0.04
 C3-C5 ≈ C3A-C5A
 with sigma of 0.04
 S4-C7 ≈ S4A-C7A
 with sigma of 0.04
 S4-C6 ≈ S4A-C6A
 with sigma of 0.04
 C7-C5 ≈ C7A-C5A
 with sigma of 0.04
3. Restrained planarity
 C3, S4, C7, C6, C5, C2
 with sigma of 0.1
 C3A, C6A, S4A, C5A, C7A, C2
 with sigma of 0.1
4. Rigid body (RIGU) restrains
 All non-hydrogen atoms
 with sigma for 1-2 distances of 0.004 and sigma for 1-3 distances of 0.004
 All non-hydrogen atoms
 with sigma for 1-2 distances of 0.002 and sigma for 1-3 distances of 0.002
5. Others
 Sof(H2BC)=Sof(H2BD)=Sof(C3A)=Sof(S4A)=Sof(C7A)=Sof(H7A)=Sof(C6A)=Sof(H6A)=
 Sof(C5A)=Sof(H5A)=1-FVAR(1)
 Sof(H2AA)=Sof(H2AB)=Sof(C3)=Sof(S4)=Sof(C7)=Sof(H7)=Sof(C6)=Sof(H6)=Sof(C5)=
 Sof(H5)=FVAR(1)
6.a Secondary CH2 refined with riding coordinates:
 C2(H2AA,H2AB), C2(H2BC,H2BD)
6.b Aromatic/amide H refined with riding coordinates:
 C7(H7), C6(H6), C5(H5), C7A(H7A), C6A(H6A), C5A(H5A)
6.c Idealised Me refined as rotating group:
 N1(H1A,H1B,H1C)

This report has been created with Olex2, compiled on 2024.02.16 svn.r378c4104 for OlexSys. Please [let us know](mailto:support@olex2.org?subject=Olex2%20Report) if there are any errors or if you would like to have additional features.
