# Supplementary material for: Temperature-Dependent Structural and Optoelectronic Properties of the Layered Perovskite 2-Thiophenemethylammonium Lead Iodide
Source: J Phys Chem C Nanomater Interfaces. 2024 Jul 25;128(31):13108–20. doi: 10.1021/acs.jpcc.4c03221 (PMC11317984; doi:10.1021/acs.jpcc.4c03221)
Supplement: Supplementary file 1 — jp4c03221_si_001.zip [file jp4c03221_si_001.zip › ThMA2PbI4_Temp-depSCXRD/datreport_175k.docx]

**ThMA2PbI4_4_175**

| **Table 1 Crystal data and structure refinement for ThMA2PbI4_4_175.** | |
| --- | --- |
| Identification code | ThMA2PbI4_4_175 |
| Empirical formula | C_20_H_64_I_8_N_4_Pb_2_S_4_ |
| Formula weight | 1918.57 |
| Temperature/K | 175.00(10) |
| Crystal system | orthorhombic |
| Space group | Cmce |
| a/Å | 29.0854(9) |
| b/Å | 8.6956(2) |
| c/Å | 8.7051(2) |
| α/° | 90 |
| β/° | 90 |
| γ/° | 90 |
| Volume/Å^3^ | 2201.65(10) |
| Z | 2 |
| ρ_calc_g/cm^3^ | 2.894 |
| μ/mm^‑1^ | 13.455 |
| F(000) | 1728.0 |
| Crystal size/mm^3^ | 1.0 × 0.08 × 0.02 |
| Radiation | Mo Kα (λ = 0.71073) |
| 2Θ range for data collection/° | 5.602 to 54.968 |
| Index ranges | -37 ≤ h ≤ 37, -11 ≤ k ≤ 11, -11 ≤ l ≤ 11 |
| Reflections collected | 9265 |
| Independent reflections | 1289 [R_int_ = 0.0375, R_sigma_ = 0.0227] |
| Data/restraints/parameters | 1289/342/134 |
| Goodness-of-fit on F^2^ | 1.309 |
| Final R indexes [I>=2σ (I)] | R_1_ = 0.0358, wR_2_ = 0.0786 |
| Final R indexes [all data] | R_1_ = 0.0374, wR_2_ = 0.0790 |
| Largest diff. peak/hole / e Å^-3^ | 1.76/-1.87 |

| **Table 2 Fractional Atomic Coordinates (×10^4^) and Equivalent Isotropic Displacement Parameters (Å^2^×10^3^) for ThMA2PbI4_4_175. U_eq_ is defined as 1/3 of the trace of the orthogonalised U_IJ_ tensor.** | | | | |
| --- | --- | --- | --- | --- |
| **Atom** | ***x*** | ***y*** | ***z*** | **U(eq)** |
| Pb01 | 5000 | 5000 | -5000 | 22.95(18) |
| I002 | 5000 | 6839.6(9) | -1841.9(9) | 29.3(2) |
| I003 | 6098.9(3) | 5000 | -5000 | 42.4(3) |
| N7 | 5940(30) | 5790(90) | 820(90) | 39(4) |
| C5 | 6668(9) | 5020(30) | -160(50) | 51(5) |
| C2 | 7485(9) | 4350(40) | -10(80) | 43(7) |
| C6A | 6172(11) | 5070(80) | -420(50) | 47(5) |
| C4 | 6955(9) | 6220(50) | -350(70) | 50(7) |
| C3 | 7402(11) | 5860(40) | -200(70) | 46(7) |
| S1 | 6975(4) | 3377(15) | 200(30) | 61(4) |
| C5A | 6678(9) | 5090(50) | 80(30) | 51(5) |
| C2A | 7473(9) | 5270(60) | -640(40) | 47(8) |
| C4A | 6974(9) | 5010(60) | 1260(50) | 53(7) |
| C3A | 7422(11) | 5080(60) | 880(40) | 43(7) |
| S1A | 6962(4) | 5350(20) | -1627(16) | 62(4) |
| C6 | 6158(11) | 4590(50) | -130(90) | 47(5) |
| N7A | 5910(30) | 5840(90) | 820(90) | 39(4) |

| **Table 3 Anisotropic Displacement Parameters (Å^2^×10^3^) for ThMA2PbI4_4_175. The Anisotropic displacement factor exponent takes the form: -2π^2^[h^2^a*^2^U_11_+2hka*b*U_12_+…].** | | | | | | |
| --- | --- | --- | --- | --- | --- | --- |
| **Atom** | **U_11_** | **U_22_** | **U_33_** | **U_23_** | **U_13_** | **U_12_** |
| Pb01 | 27.1(3) | 20.8(3) | 20.9(3) | 0.0(2) | 0 | 0 |
| I002 | 38.7(4) | 24.2(4) | 24.9(4) | -7.4(3) | 0 | 0 |
| I003 | 27.8(5) | 49.5(6) | 50.0(6) | 0.4(4) | 0 | 0 |
| N7 | 26(8) | 43(9) | 49(9) | 5(7) | 0(7) | -11(7) |
| C5 | 32(6) | 64(10) | 58(12) | 7(9) | 1(10) | 0(9) |
| C2 | 26(10) | 53(13) | 50(18) | 1(12) | 4(14) | -4(11) |
| C6A | 34(6) | 55(9) | 52(10) | 1(8) | 1(7) | -4(8) |
| C4 | 31(9) | 62(12) | 58(16) | 0(13) | 6(12) | 2(9) |
| C3 | 31(9) | 55(12) | 52(17) | 4(12) | 7(12) | -1(10) |
| S1 | 38(6) | 41(6) | 105(12) | 2(7) | 5(7) | -1(5) |
| C5A | 32(6) | 64(11) | 57(11) | 7(9) | 3(9) | -1(10) |
| C2A | 28(11) | 60(20) | 50(14) | -1(14) | -2(11) | -4(14) |
| C4A | 34(9) | 66(16) | 57(12) | 7(13) | 2(9) | -4(12) |
| C3A | 33(9) | 49(18) | 48(13) | -5(13) | -2(10) | -2(13) |
| S1A | 37(6) | 104(12) | 45(7) | 9(7) | -3(5) | 1(6) |
| C6 | 34(6) | 55(9) | 52(10) | 1(8) | 1(7) | -4(8) |
| N7A | 26(8) | 43(9) | 49(9) | 5(7) | 0(7) | -11(7) |

| **Table 4 Bond Lengths for ThMA2PbI4_4_175.** | | | | | | |
| --- | --- | --- | --- | --- | --- | --- |
| **Atom** | **Atom** | **Length/Å** |  | **Atom** | **Atom** | **Length/Å** |
| Pb01 | I002 | 3.1807(8) |  | C2 | C3 | 1.344(18) |
| Pb01 | I002^1^ | 3.1807(8) |  | C2 | S1 | 1.72(2) |
| Pb01 | I002^2^ | 3.1817(8) |  | C6A | C5A | 1.534(15) |
| Pb01 | I002^3^ | 3.1817(8) |  | C6A | N7A | 1.477(16) |
| Pb01 | I003 | 3.1963(9) |  | C4 | C3 | 1.344(18) |
| Pb01 | I003^1^ | 3.1964(9) |  | C5A | C4A | 1.344(18) |
| N7 | C6 | 1.477(16) |  | C5A | S1A | 1.72(2) |
| C5 | C4 | 1.344(18) |  | C2A | C3A | 1.344(18) |
| C5 | S1 | 1.72(2) |  | C2A | S1A | 1.72(2) |
| C5 | C6 | 1.531(15) |  | C4A | C3A | 1.344(18) |

^1^1-X,1-Y,-1-Z; ^2^+X,-1/2+Y,-1/2-Z; ^3^1-X,3/2-Y,-1/2+Z

| **Table 5 Bond Angles for ThMA2PbI4_4_175.** | | | | | | | | |
| --- | --- | --- | --- | --- | --- | --- | --- | --- |
| **Atom** | **Atom** | **Atom** | **Angle/˚** |  | **Atom** | **Atom** | **Atom** | **Angle/˚** |
| I002 | Pb01 | I002^1^ | 180.00(3) |  | C4 | C5 | S1 | 110(2) |
| I002^1^ | Pb01 | I002^2^ | 90.067(7) |  | C4 | C5 | C6 | 143(3) |
| I002 | Pb01 | I002^2^ | 89.933(7) |  | C6 | C5 | S1 | 107(3) |
| I002^1^ | Pb01 | I002^3^ | 89.933(7) |  | C3 | C2 | S1 | 110(3) |
| I002 | Pb01 | I002^3^ | 90.067(7) |  | N7A | C6A | C5A | 106(4) |
| I002^2^ | Pb01 | I002^3^ | 180.000(14) |  | C5 | C4 | C3 | 114(4) |
| I002 | Pb01 | I003 | 90.0 |  | C2 | C3 | C4 | 114(4) |
| I002^2^ | Pb01 | I003 | 90.0 |  | C5 | S1 | C2 | 91.1(18) |
| I002^1^ | Pb01 | I003^1^ | 90.0 |  | C6A | C5A | S1A | 102(3) |
| I002^3^ | Pb01 | I003 | 90.0 |  | C4A | C5A | C6A | 146(3) |
| I002^3^ | Pb01 | I003^1^ | 90.0 |  | C4A | C5A | S1A | 111(2) |
| I002^1^ | Pb01 | I003 | 90.0 |  | C3A | C2A | S1A | 114(3) |
| I002^2^ | Pb01 | I003^1^ | 90.0 |  | C5A | C4A | C3A | 115(4) |
| I002 | Pb01 | I003^1^ | 90.0 |  | C2A | C3A | C4A | 111(4) |
| I003 | Pb01 | I003^1^ | 180.0 |  | C5A | S1A | C2A | 88.8(18) |
| Pb01 | I002 | Pb01^4^ | 150.45(3) |  | N7 | C6 | C5 | 105(4) |

^1^1-X,1-Y,-1-Z; ^2^+X,-1/2+Y,-1/2-Z; ^3^1-X,3/2-Y,-1/2+Z; ^4^1-X,3/2-Y,1/2+Z

| **Table 6 Torsion Angles for ThMA2PbI4_4_175.** | | | | | | | | | | |
| --- | --- | --- | --- | --- | --- | --- | --- | --- | --- | --- |
| **A** | **B** | **C** | **D** | **Angle/˚** |  | **A** | **B** | **C** | **D** | **Angle/˚** |
| C5 | C4 | C3 | C2 | 5(5) |  | C5A | C4A | C3A | C2A | 2(4) |
| C6A | C5A | C4A | C3A | 179.3(18) |  | C4A | C5A | S1A | C2A | 2(2) |
| C6A | C5A | S1A | C2A | -179.2(12) |  | C3A | C2A | S1A | C5A | -1(3) |
| C4 | C5 | S1 | C2 | -3(3) |  | S1A | C5A | C4A | C3A | -3(3) |
| C4 | C5 | C6 | N7 | -45(5) |  | S1A | C2A | C3A | C4A | 0(4) |
| C3 | C2 | S1 | C5 | 5(3) |  | C6 | C5 | C4 | C3 | 178(3) |
| S1 | C5 | C4 | C3 | -1(4) |  | C6 | C5 | S1 | C2 | 178(2) |
| S1 | C5 | C6 | N7 | 134(5) |  | N7A | C6A | C5A | C4A | 34(6) |
| S1 | C2 | C3 | C4 | -7(4) |  | N7A | C6A | C5A | S1A | -144(5) |

| **Table 7 Hydrogen Atom Coordinates (Å×10^4^) and Isotropic Displacement Parameters (Å^2^×10^3^) for ThMA2PbI4_4_175.** | | | | |
| --- | --- | --- | --- | --- |
| **Atom** | ***x*** | ***y*** | ***z*** | **U(eq)** |
| H7A | 5965.91 | 6715.84 | 335.83 | 47 |
| H7B | 6079.39 | 5837.35 | 1750.14 | 47 |
| H7C | 5635.83 | 5564.33 | 948 | 47 |
| H2 | 7780.91 | 3887.84 | 3.9 | 52 |
| H6AA | 6133.74 | 5633.07 | -1403.94 | 57 |
| H6AB | 6062.22 | 4004.57 | -559.93 | 57 |
| H4 | 6851.18 | 7232.28 | -573.49 | 61 |
| H3 | 7639.94 | 6604.53 | -233 | 55 |
| H2A | 7764.14 | 5358.58 | -1126.76 | 56 |
| H4A | 6874.43 | 4903.05 | 2296.39 | 63 |
| H3A | 7668.38 | 5006.46 | 1592.13 | 52 |
| H6A | 6027.57 | 4573.89 | -1176.78 | 57 |
| H6B | 6114.74 | 3558.81 | 343.44 | 57 |
| H7AA | 6063.47 | 6718.06 | 1102.11 | 47 |
| H7AB | 5890.82 | 5202.68 | 1640.58 | 47 |
| H7AC | 5626.92 | 6088.28 | 476.95 | 47 |

| **Table 8 Atomic Occupancy for ThMA2PbI4_4_175.** | | | | | | | |
| --- | --- | --- | --- | --- | --- | --- | --- |
| **Atom** | ***Occupancy*** |  | **Atom** | ***Occupancy*** |  | **Atom** | ***Occupancy*** |
| N7 | 0.252(6) |  | H7A | 0.504(12) |  | H7B | 0.504(12) |
| H7C | 0.504(12) |  | C5 | 0.252(6) |  | C2 | 0.252(6) |
| H2 | 0.504(12) |  | C6A | 0.248(6) |  | H6AA | 0.496(12) |
| H6AB | 0.496(12) |  | C4 | 0.252(6) |  | H4 | 0.504(12) |
| C3 | 0.252(6) |  | H3 | 0.504(12) |  | S1 | 0.252(6) |
| C5A | 0.248(6) |  | C2A | 0.248(6) |  | H2A | 0.496(12) |
| C4A | 0.248(6) |  | H4A | 0.496(12) |  | C3A | 0.248(6) |
| H3A | 0.496(12) |  | S1A | 0.248(6) |  | C6 | 0.252(6) |
| H6A | 0.504(12) |  | H6B | 0.504(12) |  | N7A | 0.248(6) |
| H7AA | 0.496(12) |  | H7AB | 0.496(12) |  | H7AC | 0.496(12) |

**Experimental**

Single crystals of C_20_H_64_I_8_N_4_Pb_2_S_4_ **[ThMA2PbI4_4_175]** were **[]**. A suitable crystal was selected and **[]** on a **XtaLAB Synergy, Dualflex, HyPix-Arc 100** diffractometer. The crystal was kept at 175.00(10) K during data collection. Using Olex2 [1], the structure was solved with the SHELXT [2] structure solution program using Intrinsic Phasing and refined with the SHELXL [3] refinement package using Least Squares minimisation.

1. Dolomanov, O.V., Bourhis, L.J., Gildea, R.J, Howard, J.A.K. & Puschmann, H. (2009), J. Appl. Cryst. 42, 339-341.
2. Sheldrick, G.M. (2015). Acta Cryst. A71, 3-8.
3. Sheldrick, G.M. (2015). Acta Cryst. C71, 3-8.

**Crystal structure determination of [ThMA2PbI4_4_175]**

**Crystal Data** for C_20_H_64_I_8_N_4_Pb_2_S_4_ (*M*=1918.57 g/mol): orthorhombic, space group Cmce (no. 64), *a* = 29.0854(9) Å, *b* = 8.6956(2) Å, *c* = 8.7051(2) Å, *V*= 2201.65(10) Å^3^, *Z* = 2, *T* = 175.00(10) K, μ(Mo Kα) = 13.455 mm^-1^, *Dcalc* = 2.894 g/cm^3^, 9265 reflections measured (5.602° ≤ 2Θ ≤ 54.968°), 1289 unique (*R*_int_ = 0.0375, R_sigma_ = 0.0227) which were used in all calculations. The final *R*_1_ was 0.0358 (I > 2σ(I)) and *wR*_2_ was 0.0790 (all data).

**Refinement model description**

Number of restraints - 342, number of constraints - unknown.

Details:

1. Fixed Uiso
 At 1.2 times of:
 All C(H) groups, All C(H,H) groups, All N(H,H,H) groups
2. Restrained distances
 C6A-C5A = C6-C5
 1.54 with sigma of 0.02
 N7-C6 = N7A-C6A
 1.48 with sigma of 0.02
 N7A-C6A ≈ N7-C6
 with sigma of 0.02
 C6A-C5A ≈ C6-C5
 with sigma of 0.02
 S1-C2 ≈ S1-C5 ≈ S1A-C2A ≈ S1A-C5A
 with sigma of 0.002
 C5A-C4A ≈ C4A-C3A ≈ C3A-C2A ≈ C3-C2 ≈ C4-C3 ≈ C5-C4
 with sigma of 0.002
3. Restrained planarity
 C6A, C5A, S1A, C2A, C3A, C4A
 with sigma of 0.1
 C6, C5, C4, C3, C2, S1
 with sigma of 0.1
4. Uiso/Uaniso restraints and constraints
All non-hydrogen atoms have similar U: within 2A with sigma of 0.04 and sigma
for terminal atoms of 0.08 within 2A
C6A ≈ C6 ≈ C5A ≈ C5: within 2A with sigma of 0.02 and sigma for
terminal atoms of 0.04 within 2A
N7 ≈ N7A ≈ C5A ≈ C5 ≈ C6A ≈ C6: within 2A with sigma of
0.02 and sigma for terminal atoms of 0.04 within 2A
Uanis(C6A) = Uanis(C6)
Uanis(N7A) = Uanis(N7)
5. Rigid body (RIGU) restrains
 All non-hydrogen atoms
 with sigma for 1-2 distances of 0.004 and sigma for 1-3 distances of 0.004
6. Others
 Sof(H6AA)=Sof(H6AB)=Sof(H2A)=Sof(H4A)=Sof(H3A)=Sof(H7AA)=Sof(H7AB)=Sof(H7AC)=
 1-FVAR(1)
 Sof(C6A)=Sof(C5A)=Sof(C2A)=Sof(C4A)=Sof(C3A)=Sof(S1A)=Sof(N7A)=0.5*(1-FVAR(2))
 Sof(N7)=Sof(C5)=Sof(C2)=Sof(C4)=Sof(C3)=Sof(S1)=Sof(C6)=0.5*FVAR(2)
 Sof(H7A)=Sof(H7B)=Sof(H7C)=Sof(H2)=Sof(H4)=Sof(H3)=Sof(H6A)=Sof(H6B)=FVAR(1)
7.a Secondary CH2 refined with riding coordinates:
 C6A(H6AA,H6AB), C6(H6A,H6B)
7.b Aromatic/amide H refined with riding coordinates:
 C2(H2), C4(H4), C3(H3), C2A(H2A), C4A(H4A), C3A(H3A)
7.c Idealised Me refined as rotating group:
 N7(H7A,H7B,H7C), N7A(H7AA,H7AB,H7AC)

This report has been created with Olex2, compiled on 2024.02.16 svn.r378c4104 for OlexSys. Please [let us know](mailto:support@olex2.org?subject=Olex2%20Report) if there are any errors or if you would like to have additional features.
