# Supplementary material for: Temperature-Dependent Structural and Optoelectronic Properties of the Layered Perovskite 2-Thiophenemethylammonium Lead Iodide
Source: J Phys Chem C Nanomater Interfaces. 2024 Jul 25;128(31):13108–20. doi: 10.1021/acs.jpcc.4c03221 (PMC11317984; doi:10.1021/acs.jpcc.4c03221)
Supplement: Supplementary file 2 — jp4c03221_si_002.pdf [file jp4c03221_si_002.pdf]

Supporting Information for: Temperature-dependent Structural and Optoelectronic Properties of the Layered Perovskite 2-thiophenemethylammonium Lead Iodide

Justas Deveikis,† Marcin Giza,‡ David Walker,† Jie Liu, † Claire Wilson,‡ Nathaniel P. Gallop,† Pablo Docampo,‡ James Lloyd-Hughes,*,† and Rebecca L. Milot*,†

†Department of Physics, University of Warwick, Coventry, United Kingdom
‡School of Chemistry, University of Glasgow, Glasgow, United Kingdom

Contents

1. Single-crystal XRD data: organic cation....2
2. Differential scanning calorimetry measurement....3
3. Single-crystal XRD data: lattice parameters dependence on temperature....4
  3.1. $ThMA_2PbI_4$ crystal structure parameters comparison at different temperatures....6
  3.2. Enlarged $ThMA_2PbI_4$ crystal structures....8
4. Thin film XRD data....9
5. UV-Vis absorption....10
  5.1. UV-Vis absorption temperature-dependent data....10
  5.2. Elliott model description....11
  5.3. UV-Vis absorption data fitting to Elliott model....13
6. Power-dependent photoluminescence....14
References....15

1. Single-crystal XRD data: organic cation

Single-crystal XRD structures of $ThMA_2PbI_4$ acquired at temperatures in the range 100-300 K are provided in .cif files. Selected views of the structures at 100 K and 300 K are presented below.

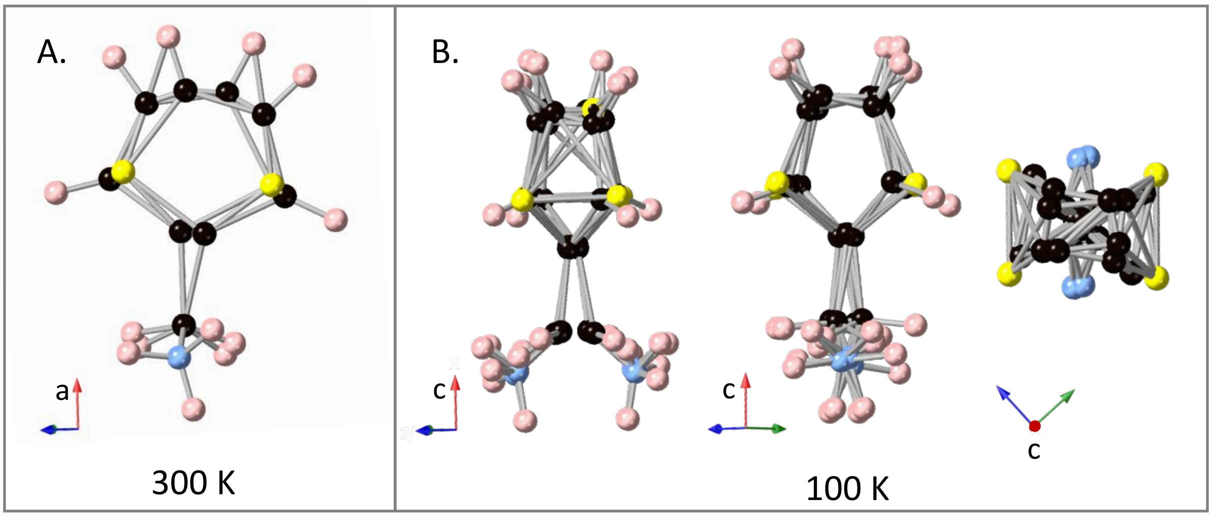

Figure S1. Disordered behaviours of the ThMA cations observed in the single-crystal structures at (A) 300 K and (B) 100 K.

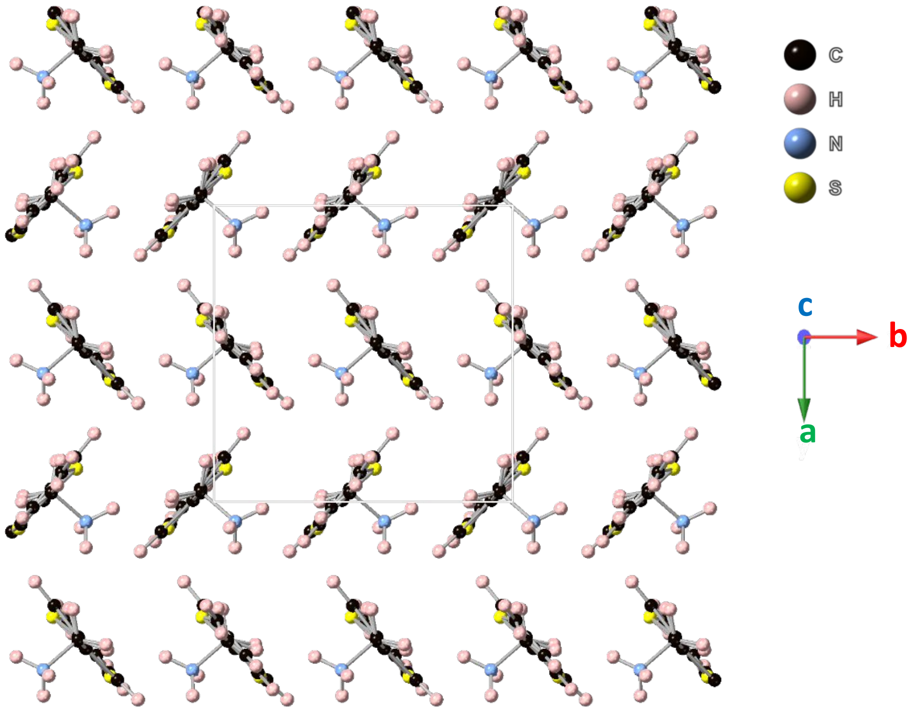

Figure S2. The ThMA bilayer viewed along the c-axis highlighting the stacking pattern of the organic cations. Crystal structure was obtained at 300 K.

2. Differential scanning calorimetry measurement

To investigate the structural phase transition in strain-free environment, we performed the differential scanning calorimetry (DSC) measurement using powder samples of $ThMA_2PbI_4$ on METTLER TOLEDO under nitrogen atmosphere. The specimens, of mass around 5 mg, were placed in aluminium pans and the software STAR^e *V*16.40 was used for the data analysis. The temperature range is from 100K to 300K, the cooling and heating rate is 10K/min for both cooling and heating process. The DSC curves are presented in Fig. S10. The peaks in the heating and cooling runs indicate a reversible phase transition, which occurred around the temperature 225.81 K (onset value of the peak during the heating process) and 223.43 K during the cooling process. The phase transition temperatures obtained from temperature-dependent thin film and single crystal measurements agree with the temperatures from the DSC experiment.

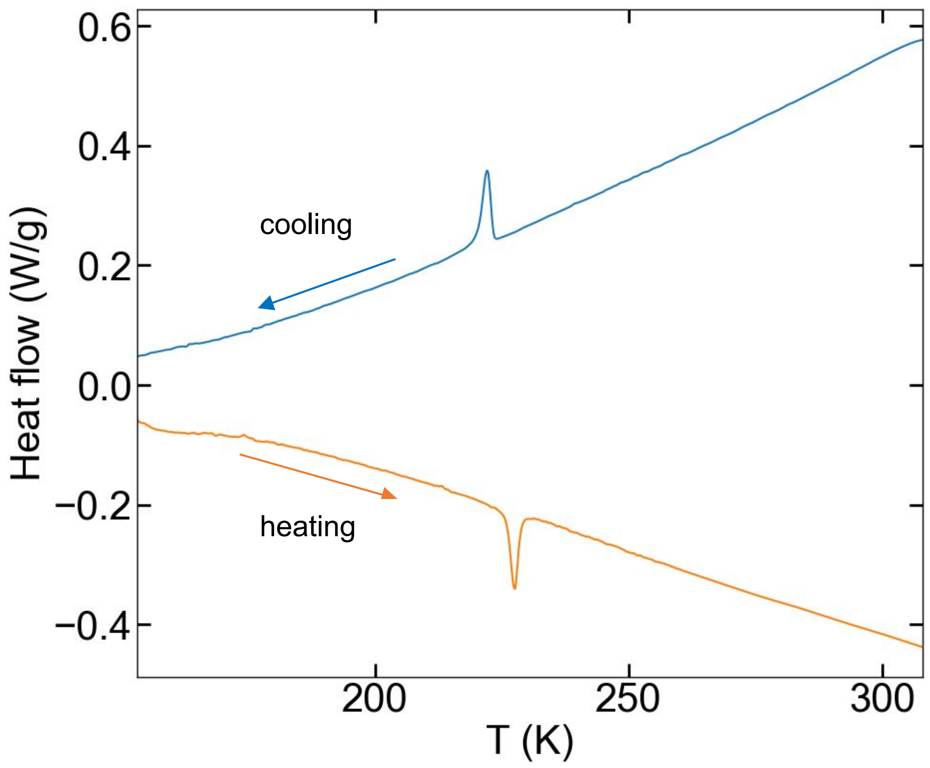

Figure S3. Differential scanning calorimetry (DSC) measurement on $ThMA_2PbI_4$ powder on heating and cooling cycle. Onset temperature on heating: 225.81 K; on cooling: 223.43 K.

3. Single-crystal XRD data: lattice parameters dependence on temperature

Single crystal data allowed us to analyse the temperature evolution of crystal lattice factors, such as volume, Pb-I bond length and *a*, *b*, *c* lattice parameters, which are presented in Fig. S3. We selected to present the temperature-dependent lattice parameters of two different space groups (*C*mce and *P*bca) to match the physical features of the crystal lattice of $ThMA_2PbI_4$, e.g. the lattice parameter *a* of *C*mce and *c* of *P*bca space groups both correspond to the spacing between the inorganic layers (Fig. S4 (C)).

We also attempted to quantify the linear thermal expansion coefficients along the crystal axes *a*, *b* and *c* in both phases by using linear fits, which are represented by solid lines in Fig. S4 (C) and (D). As we had only three points available in the high-temperature phase, we acknowledge that linear thermal expansion coefficients are not accurate, and we do not put much emphasis on these values in the paper.

A.

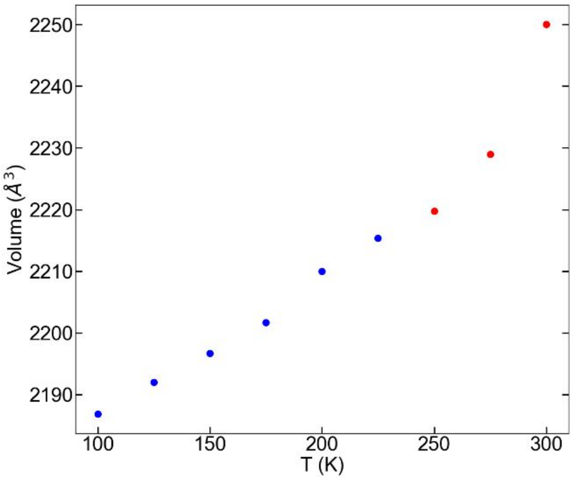

B.

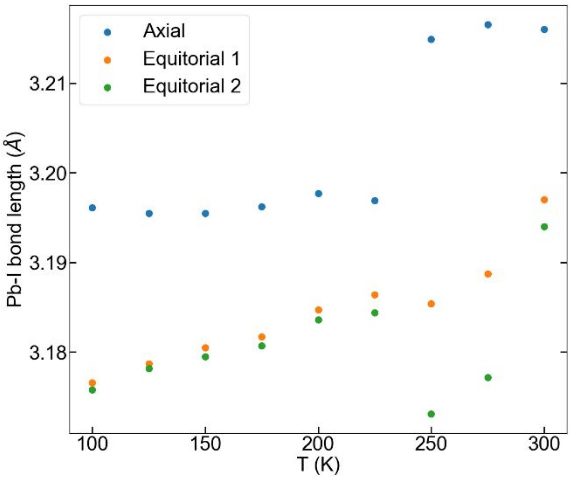

C.

D.

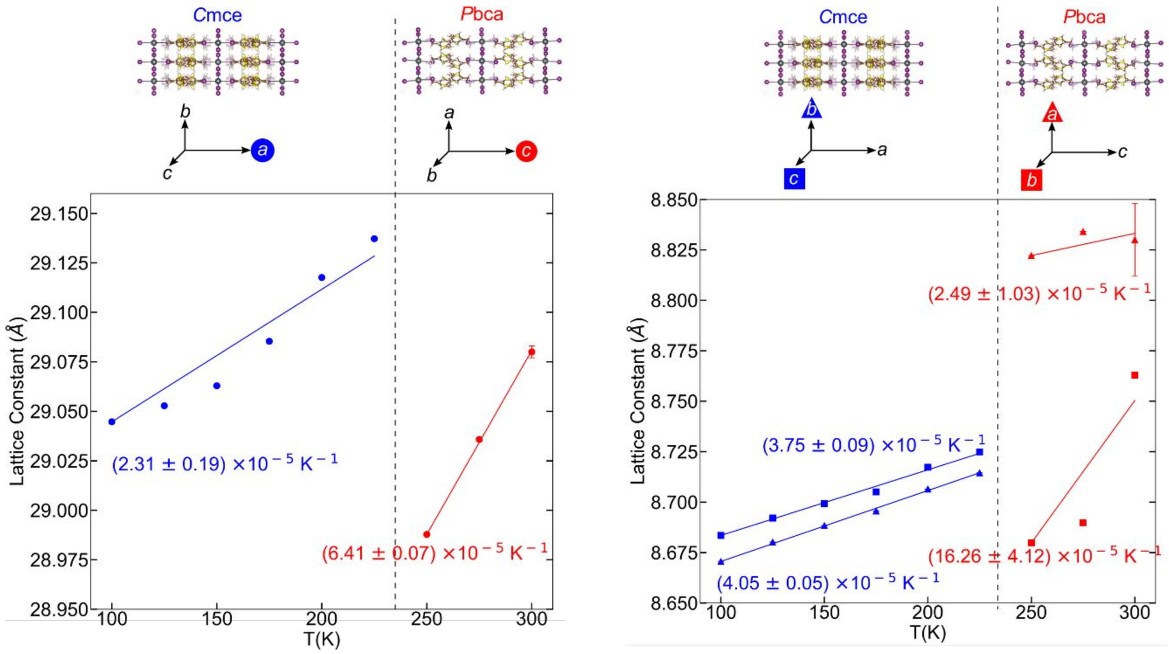

Figure S4. Temperature dependence of the (A) total unit cell volume, (B) Pb-I bond lengths (C) and(D) lattice parameters of $ThMA_2PbI_4$ as determined from single-crystal XRD measurements. The thermal expansion coefficients determined for both the low and high temperature phases from fits to Eq. 1 are indicated on the graphs.

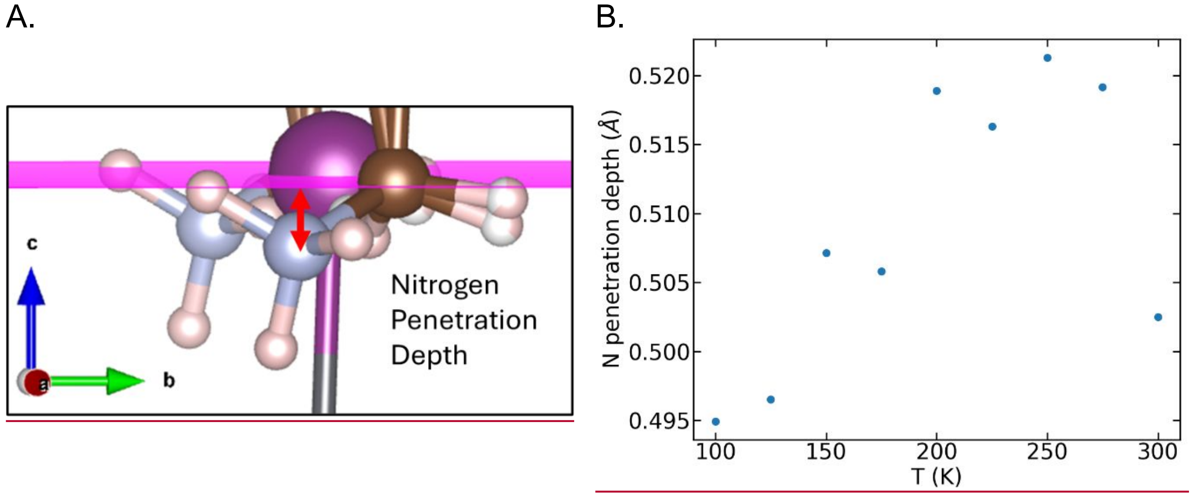

Figure S5. Determination of the nitrogen penetration depth. (A) Diagram illustrating the penetration depth, which is defined as the orthogonal distance between the nitrogen and iodine plane. (B) Temperature dependence of the nitrogen penetration depth measured from temperature-dependent XRD measurements of $ThMA_2PbI_4$.

3.1. $ThMA_2PbI_4$ crystal structure parameters comparison at different temperatures

The list of structural properties $ThMA_2PbI_4$ of single crystal at temperature range 100-300 K investigated is provided in the Table S1.

Table S1. Bond lengths of Pb-I, bond distortion indexes, angles of Pb-I-Pb and nitrogen penetration depths of $ThMA_2PbI_4$ single crystal at temperature range 100-300K.

**Bond Lengths**	**100K (Cmce)**	**125K (Cmce)**	**150K (Cmce)**	**175K (Cmce)**	**200K (Cmce)**	**225K (Cmce)**	**250K (Pbca)**	**275K (Pbca)**	**300K (Pbca)**
**Axial Pb-I (Å)**	3.1961	3.1955	3.1955	3.1962	3.1977	3.1969	3.2149	3.2165	3.216
	3.1961	3.1955	3.1955	3.1962	3.1977	3.1969	3.2149	3.2165	3.216
**Equitorial Pb-I (Å)**	3.1758	3.1782	3.1795	3.1807	3.1836	3.1844	3.1731	3.1772	3.194
	3.1766	3.1787	3.1805	3.1817	3.1847	3.1864	3.1854	3.1887	3.197
	3.1758	3.1782	3.1795	3.1807	3.1836	3.1844	3.1731	3.1772	3.194
	3.1766	3.1787	3.1805	3.1817	3.1847	3.1864	3.1854	3.1887	3.197
**Average Pb-I (Å)**	3.18283	3.18413	3.18517	3.18620	3.18867	3.18923	3.19113	3.19413	3.20233
**Bond Distortion Index**	**0.002778796**	**0.002379856**	**0.002162803**	**0.002092357**	**0.001888633**	**0.001602614**	**0.004965146**	**0.004668281**	**0.002845148**
**Angles**	**100K (Cmce)**	**125K (Cmce)**	**150K (Cmce)**	**175K (Cmce)**	**200K (Cmce)**	**225K (Cmce)**	**250K (Pbca)**	**275K (Pbca)**	**300K (Pbca)**
**Pb-I-Pb (°)**	149.98	150.12	150.3	150.45	150.63	150.85	153.415	153.452	153.428
**I-Pb-I (Axial) (°)**	90	90	90	90	90	90	89.312	89.31	89.271
	90	90	90	90	90	90	90.688	90.69	90.729
	90	90	90	90	90	90	85.612	85.719	85.972
	90	90	90	90	90	90	94.388	94.281	94.028
	90	90	90	90	90	90	89.312	89.31	89.271
	90	90	90	90	90	90	90.688	90.69	90.729
	90	90	90	90	90	90	85.612	85.719	85.972
	90	90	90	90	90	90	94.388	94.281	94.028
**I-Pb-I (Equitorial) (°)**	89.911	89.919	89.923	89.933	89.924	89.922	89.09	89.072	89.52

	90.089	90.081	90.077	90.067	90.076	90.078	90.91	90.928	90.48
	89.911	89.919	89.923	89.933	89.924	89.922	89.09	89.072	89.52
	90.089	90.081	90.077	90.067	90.076	90.078	90.91	90.928	90.48
**Bond Angle Variance**	**0.002880364**	**0.002385818**	**0.002156**	**0.001632364**	**0.002100364**	**0.002212364**	**7.474904727**	**7.150634545**	**6.176954545**

**Nitrogen Penetration**	**100K (Cmce)**	**125K (Cmce)**	**150K (Cmce)**	**175K (Cmce)**	**200K (Cmce)**	**225K (Cmce)**	**250K (Pbca)**	**275K (Pbca)**	**300K (Pbca)**
**N Atom Position (Å)**	2.67211<br>2.7302	2.67286<br>2.72515	2.67379<br>2.70285	2.64677<br>2.73403	2.62058<br>2.73705	2.68062<br>2.68062	2.6776	2.68146	2.69862
**Av N Atom Position (Å)**	2.701155	2.699005	2.68832	2.6904	2.678815	2.68062	2.6776	2.68146	2.69862
**Iodine Atom Position (Å)**	3.19608	3.19552	3.19547	3.19619	3.1977	3.19693	3.1989	3.20062	3.20113
**Penetration Depth(Å)**	**0.494925**	**0.496515**	**0.50715**	**0.50579**	**0.518885**	**0.51631**	**0.5213**	**0.51916**	**0.50251**

3.2. Enlarged $ThMA_2PbI_4$ crystal structures

We provide enlarged crystal structures obtained from single-crystal XRD measurement at 100 and 300 K in Fig. S6. They are also presented in Fig. 1 in the main text.

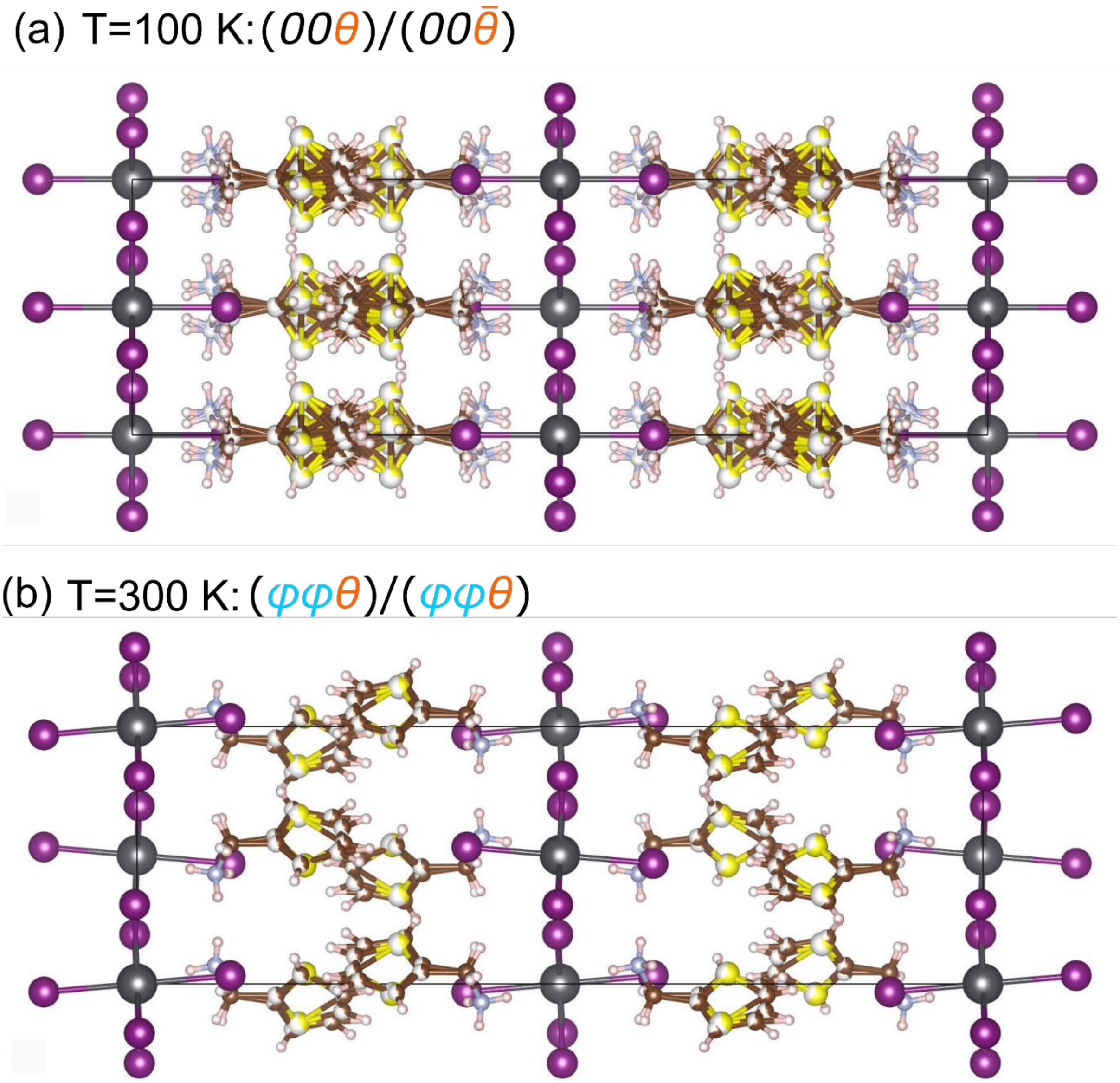

Fig. S6. $ThMA_2PbI_4$ crystal structures acquired using single crystal XRD at 100 and 300 K respectively.

4. Thin film XRD data

Thin-film XRD diffractograms in temperature range 80-300 K are given in Fig. S7.

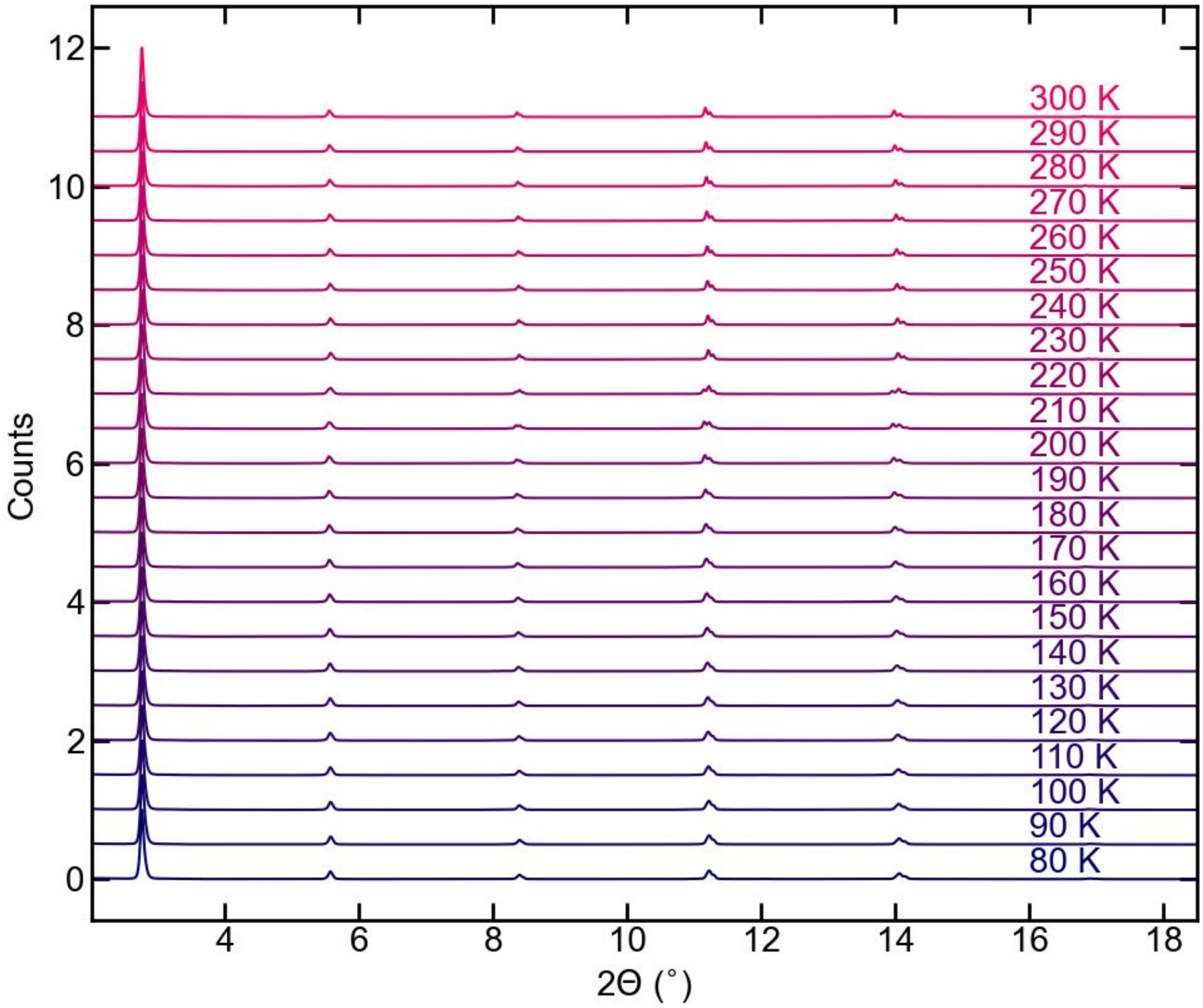

Figure S7. XRD diffractograms of $ThMA_2PbI_4$ thin film in temperature range 260-300 K. The diffractograms are shifted vertically for clarity.

5. UV-Vis absorption

5.1. UV-Vis absorption temperature-dependent data

UV-Vis absorbance data in temperature range 80-300 K is given in Fig. S8.

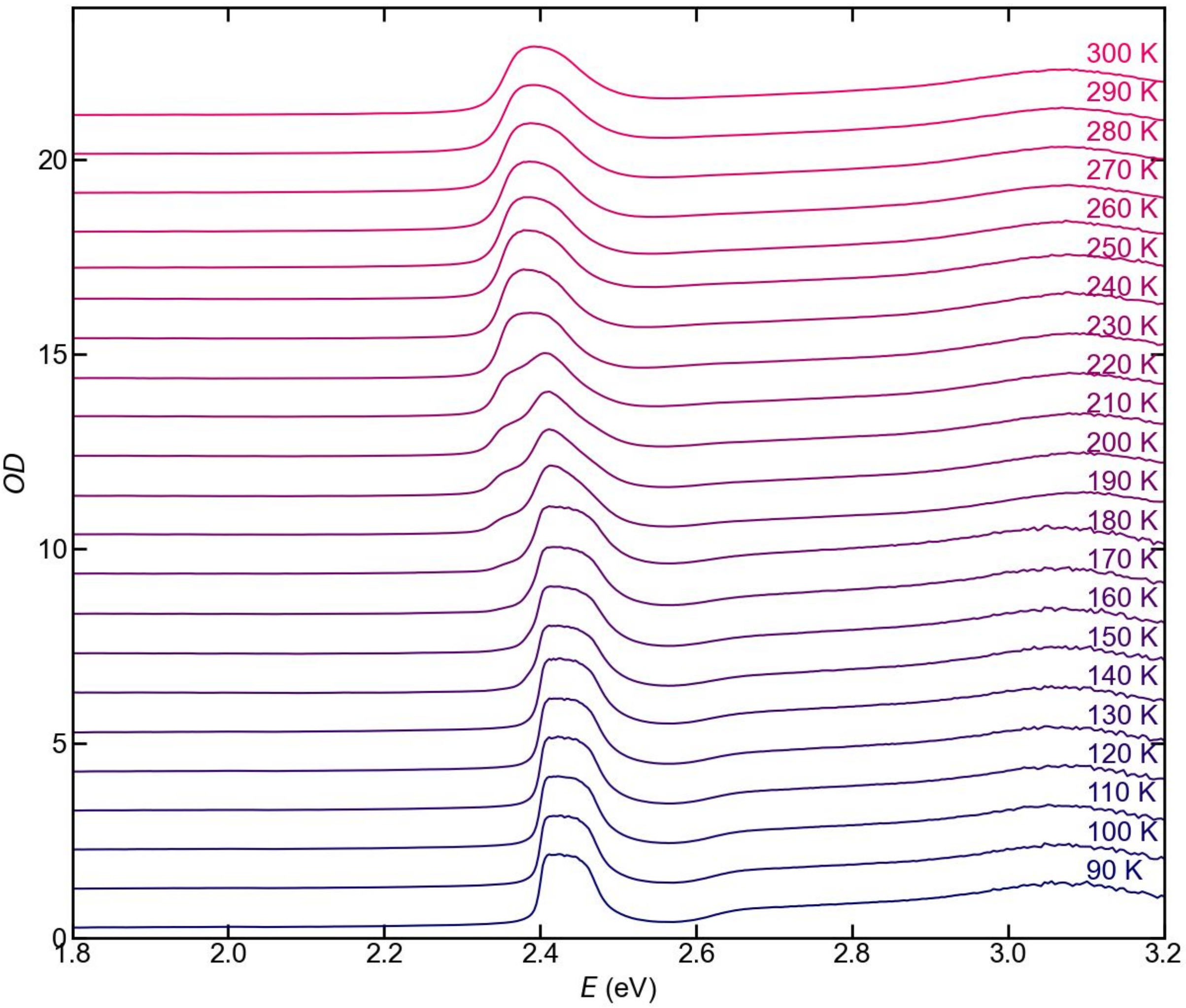

Figure S8. UV-Vis absorption spectra of $ThMA_2PbI_4$ thin film in temperature range 250-300 K. The absorption spectra are shifted vertically for clarity.

5.2. Elliott model description

To calculate the absorption coefficient from our experimental transmittance data, including the influence of thin-film interference effects, we used the Elliott model to define the complex refractive index of the perovskite layer, and the thin film transmission of the perovskite, with known thickness. For clarity, this procedure is now described in detail.

The Elliott model describes the absorption coefficient $\alpha(E)$ as a function of photon energy E of an excitonic system via [2], [3], [4].

$$\alpha(E) = \alpha_X(E) + \alpha_{continuum}(E) = A_0\left(\sum_{n=1}^{\infty} \frac{4E_b}{n^3}\delta\left(E - E_g + \frac{E_b}{n^2}\right) + \theta\left(E - E_g\right)\frac{\pi e^{\pi x}}{\sinh(\pi x)}\right), \quad \text{(S1)}$$

where A_0 is a constant proportional to the transition matrix element; n is the exciton's quantum number; E_g and E_b are the single-particle bandgap energy and exciton binding energy, respectively; θ is a step function, used to model interband absorption; δ describes the lineshape of an absorption peak of an individual excitonic state; and $x = \sqrt{E_b\left(E - E_g\right)}$.

For our model, we selected the Lorentzian function $\delta(E) = \frac{A\Gamma^2}{(E-E_0)^2+\Gamma^2}$ to model the absorption peak of excitons, where A is the relative amplitude of the Lorentzian, Γ is the broadening parameter and E_0 is the central energy of the excitonic state transition. For the continuum absorption we used a logistic function $\theta(E) = \frac{A_c}{1+e^{-k(E-E_g)}}$ where A_c is the relative amplitude of the continuum's contribution, k is the broadening parameter and E_g is the centre of the step function, in this case the bandgap energy. Exciton states from n=1 to n=5 were included in modelling, as higher excitonic states did not show a significant contribution.

The transmittance was measured experimentally, and is defined as $T = \frac{I(E)}{I_0(E)}$, where $I(E)$ and $I_0(E)$ are the transmitted and incident intensities. The power transmittance was obtained from the Fresnel transmission coefficient t via $T = |t|^2$:

$$t = \frac{\tilde{E}_{sample}}{\tilde{E}_{ref}} = \frac{t_{ij}t_{jk}e^{i\frac{\omega\tilde{n}_j d}{c}}FP_{ijk}}{t_{ik}e^{i\frac{\omega\tilde{n}_i d}{c}}}, \quad \text{(S2)}$$

where $\tilde{E}_{sample}$, $\tilde{E}_{ref}$ are the complex electric fields transmitted through the sample and reference respectively; d is the thickness of the sample; Fresnel transmission coefficient $t_{ij} = \frac{2\tilde{n}_j}{\tilde{n}_i+\tilde{n}_j}$; Fabry-Perot term $FP_{ijk} = \sum_{p=0}^{P} r_{jk}r_{ji}e^{i\frac{2\tilde{n}_j E d}{\hbar c}}$ with p being the number of total reflections; Fresnel reflection coefficient $r_{ij} = \frac{\tilde{n}_i-\tilde{n}_j}{\tilde{n}_i+\tilde{n}_j}$; $\tilde{n}_i$ is complex refractive index of the i-th layer.

The assumption that the incident beam was normal to the surface of the film was used. Here, the index i denotes medium of incident beam (air), j denotes the perovskite layer, and k denotes the substrate.

To construct the complex refractive index of the perovskite layer, $\tilde{n}_j = n + i\kappa$, we first used the absorption coefficient α parameterised by the Elliott formula to find the imaginary part via $\kappa = \frac{\alpha\hbar c}{2E}$. We then used the Kramers-Kronig relation to obtain the real part:

$$n(\omega) = 1 + \frac{2}{\pi} P \int_0^\infty \frac{\omega' \kappa(\omega')}{\omega'^2 - \omega^2} d\omega', \tag{S3}$$

where P denotes the principal part of integral; ω is the angular frequency of a photon ($\omega = \frac{E}{\hbar}$).

The Kramers-Kronig formally requires an integral from E=0 to E=∞, although experimentally we have information on κ in the measured photon energy range only (from E_{min}=2.2 eV to E_{max}=2.8 eV). To account for the contribution to E from the integral over other energy ranges (from 0 to E_{min} and $E_{min}$to ∞), we therefore added a real constant to n in Eq. S3.

By constructing this model, we were able to calculate the transmittance expected for the Elliot model and fit the UV-visible transmission spectroscopy data to obtain the exciton binding energy, bandgap energy and other parameters.

5.3. UV-Vis absorption data fitting to Elliott model

The Elliott fits of UV-Vis transmission data at 100 K and 300 K temperature are given in Fig. S9.

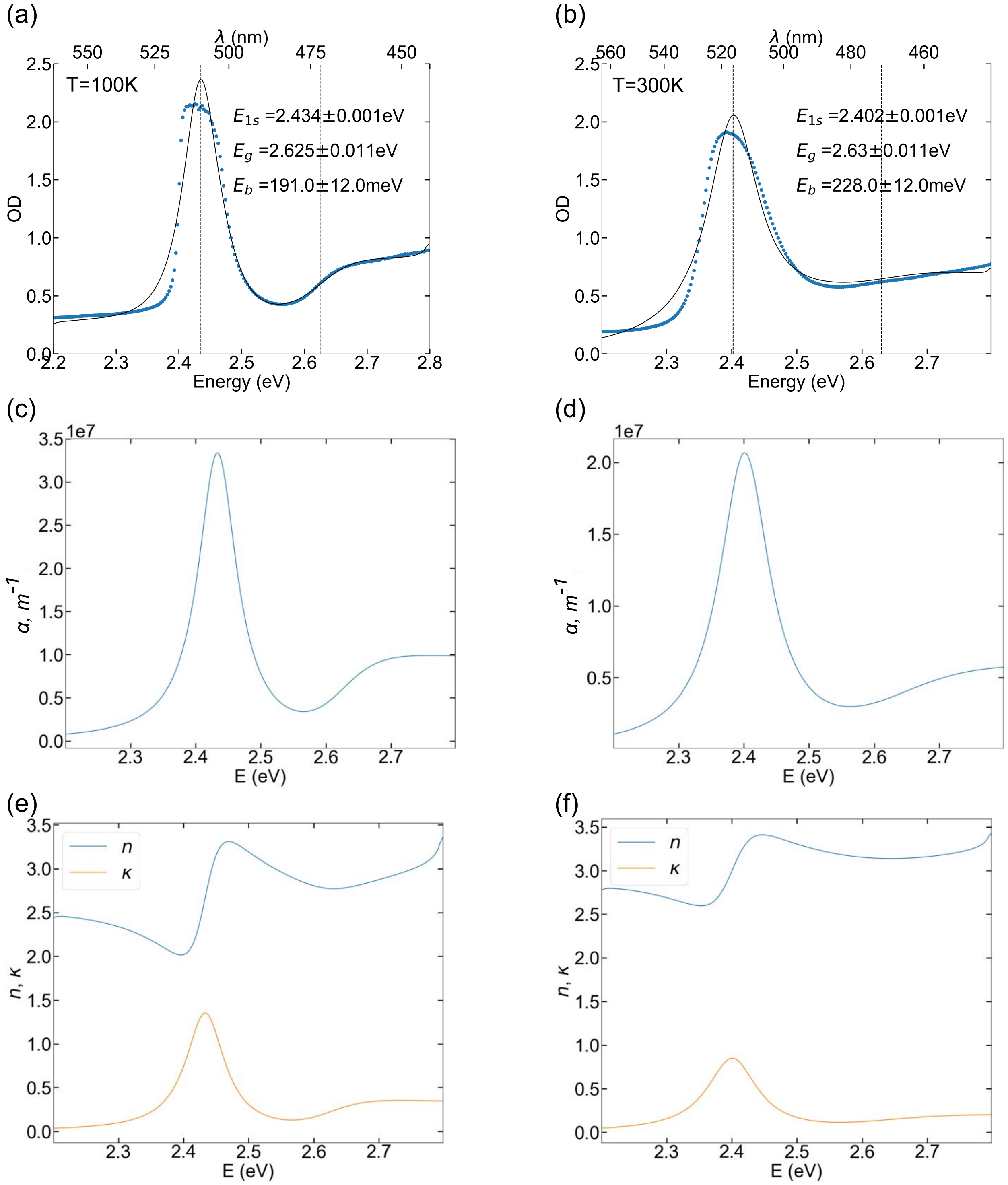

Figure S9. (a), (b) Transmittance spectra; (c), (d) absorption coefficient and (e), (f) real and imaginary parts of refractive index at 100 K and 300 K respectively.

6. Power-dependent photoluminescence

To elucidate the origin of low energy (LE) peak, we recorded the power-dependent PL experiment. We measured PL counts at two spectral ranges (2.2-2.25 eV for LE and 2.3-2.5 eV for X) at different excitation powers (Fig. S10). We demonstrated that both PL peaks have similar scaling, which confirms that both share the same origin. The analysis agrees that the LE peak is caused by the radiative recombination of defect-bound exciton.

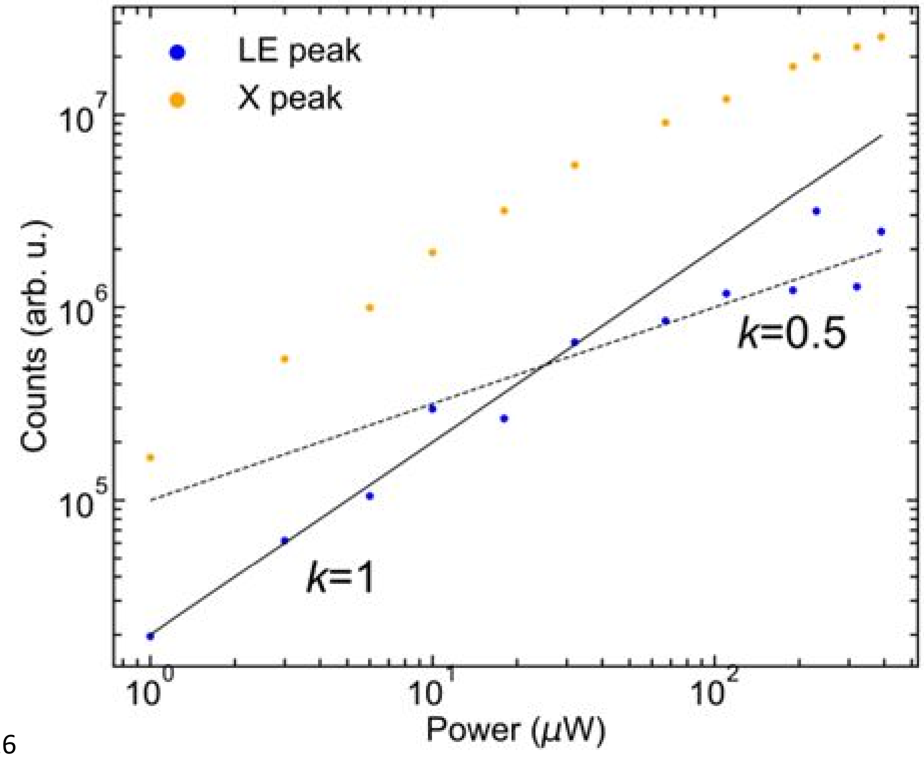

Figure S10. Excitation power-dependent PL at 100K of LE and X peak. The integration range of PL counts was 2.2-2.25 eV and 2.3-2.5 eV for LE and X peaks respectively. *k* is the exponent of the power law: $y=Ax^k$.

References

[1] M. H. Jung, "Highly Stable Thiophene Perovskite Enabled by an Oxygen-Containing Moiety for Efficient Photovoltaics," *Journal of Physical Chemistry C*, vol. 125, no. 46, pp. 25430–25445, Nov. 2021.

[2] R. J. Elliott, "Intensity of Optical Absorption by Excitons," *Physical Review*, vol. 108, no. 6, p. 1384, Dec. 1957.

[3] M. Baranowski and P. Plochocka, "Excitons in Metal-Halide Perovskites," *Adv Energy Mater*, vol. 10, no. 26, p. 1903659, Jul. 2020.

[4] Y. Yang *et al.*, "Low surface recombination velocity in solution-grown $CH_3NH_3PbBr_3$ perovskite single crystal," *Nature Communications 2015 6:1*, vol. 6, no. 1, pp. 1–6, Aug. 2015.
